# AEGIS: an operational infrastructure for post-market governance of adaptive medical AI under US and EU regulatory frameworks

**DOI:** 10.1038/s41746-026-03290-1

**Published:** 2026-10-13

**Authors:** Fardin Afdideh, Mehdi Astaraki, Fernando Seoane, Farhad Abtahi

**Affiliations:** 1 Department of Clinical Science, Intervention and Technology, Karolinska Institutet, Stockholm, Sweden; 2 Department of Medical Radiation Physics, Stockholm University, Solna, Sweden; 3 Department of Oncology-Pathology, Karolinska Institutet, Solna, Sweden; 4 Department of Clinical Physiology, Karolinska University Hospital, Stockholm, Sweden; 5 Department of Textile Technology, University of Borås, Borås, Sweden; 6 Department of Medical Technologies, Karolinska University Hospital, Huddinge, Sweden; 7 Department of Biomedical Engineering and Health System, KTH Royal Institute of Technology, Huddinge, Sweden

**Keywords:** Computational biology and bioinformatics, Engineering, Health care, Mathematics and computing, Medical research

## Abstract

Medical AI requires governance preserving safety during continuous improvement. FDA Predetermined Change Control Plans (PCCP) and EU post-market surveillance address iterative updates but lack operational tooling. We present AEGIS, an internal governance and post-market-monitoring infrastructure designed to support the documentation of predetermined governance pathways for PCCP and Medical Devices Regulation alignment. AEGIS comprises dataset assimilation and retraining, model monitoring, and conditional decision-making modules. It utilizes a four-category taxonomy (APPROVE, CONDITIONAL APPROVAL, CLINICAL REVIEW, REJECT) and an independent ALARM signal. Instantiating AEGIS for sepsis prediction (PhysioNet) and brain-tumor segmentation (BraTS) exercised all decision categories, successfully routing failures to the critical REJECT + ALARM state (no deployable model; released model at risk). On real cross-site data, drift detection ROC-AUC rose from 0.82 to 1.00, reliably triggering near 500-patient batches with zero false alarms. This proof-of-concept utilizes illustrative clinical models; drift scoring quantifies covariate shift only, leaving concept drift for future work.

## Introduction

Machine Learning (ML) models in healthcare differ fundamentally from traditional medical devices: they have the *capacity* to learn and adapt over time. In current practice, however, marketed AI/ML-enabled Medical Devices (MLMDs) are generally locked after authorization or certification, and any subsequent modification—even within a pre-authorized scope—requires controlled validation and regulatory assessment. Governing such controlled change is the regulatory challenge this work addresses. When an algorithm’s performance drifts, current frameworks provide no systematic response mechanism. The Food and Drug Administration (FDA)’s adverse event database cannot capture gradual degradation because it does not constitute a reportable event^1^.

As of June 2026, over 1500 MLMDs have received FDA clearance or authorization since the first approval in 1995^2^. These systems increasingly support clinical decision-making in diverse settings, from imaging diagnostics to acute risk estimation. Yet the regulatory infrastructure designed for static devices struggles to accommodate algorithms that improve through real-world use.

The FDA recognized this mismatch and initiated a policy shift. Beginning with a 2019 discussion paper^3^ and culminating in landmark guidance in August 2025^4^, the agency established the Predetermined Change Control Plan (PCCP) framework. Under a PCCP, manufacturers prespecify acceptable modifications and the criteria for implementing them. Once approved, changes within these bounds require no additional submissions^4^. This applies across major pathways: 510(k)^5^, De Novo^6^, and Pre-Market Approval^7^.

In the European Union (EU), the primary legal framework governing MLMDs is the Medical Devices Regulation (MDR) 2017/745, which sets the requirements for conformity assessment, clinical evaluation, post-market surveillance, vigilance, and placing the device on the market. The EU Artificial Intelligence (AI) Act adds horizontal requirements for high-risk AI systems but does not replace the MDR; manufacturers must satisfy both. We also note an instrument-hierarchy distinction used throughout this paper: the MDR and the AI Act are binding EU law, whereas the FDA PCCP guidance is a non-binding guidance document issued by a competent authority. The EU AI Act contains related provisions. Article 43(4) permits “predetermined changes” that avoid triggering new Conformity Assessments (CAs) when documented in technical documentation^8^. However, ambiguity persists around what constitutes a “substantial modification” requiring reassessment, and no EU instrument currently establishes a PCCP-equivalent pathway. The Medical Device Coordination Group and AI Board have begun issuing joint guidance to clarify implementation^9^. Both regulatory frameworks share a critical implementation gap: they specify *what* manufacturers must do without defining *how* to do it operationally. Four questions remain unanswered:

1. What specific metrics should trigger a model update or rollback?
2. What quantitative thresholds define acceptable versus unacceptable performance?
3. How should drift detection be operationalized with statistical rigor?
4. What audit trail structure ensures regulatory defensibility?

Translating these regulatory concepts into executable governance procedures remains an unsolved challenge.

Babic et al.^1^ quantified this gap through a systematic analysis of the FDA’s MAUDE database. Their review of 943 adverse events across 823 AI/ML devices (2010–2023) found the surveillance infrastructure “insufficient for properly assessing the safety and effectiveness of AI/ML devices.” Critically, concept drift and covariate shift cannot be reported because they “do not constitute an adverse event.” Aggregate performance deterioration remains invisible to case-based reporting.

While regulatory guidance establishes the need for continuous Post-Market Surveillance (PMS) of adaptive medical AI^10^, the operational decision logic required for change control remains unspecified, a gap echoed in recent governance frameworks^11^. This paper addresses these gaps by presenting AI/ML Evaluation and Governance Infrastructure for Safety (AEGIS), a technical infrastructure designed to support the operationalization of PCCP requirements through executable specifications. AEGIS provides three core components: a Dataset Assimilation and Retraining Module (DARM) for data governance, a Model Monitoring Module (MMM) for continuous surveillance, and a Conditional Decision Module (CDM) for automated governance decisions.

AEGIS parallels the FDA’s concept of Substantial Equivalence (SE) used in 510(k) submissions^5^. In the 510(k) process, manufacturers demonstrate that a new device is as safe and effective as a predicate device. AEGIS implements an analogous “fixed performance reference comparison mechanism” for model updates. New iterations must perform within defined bounds of the fixed performance reference metrics or demonstrate improvement.

This paper makes the following contributions:

1. A four-category deployment decision taxonomy (APPROVE, CONDITIONAL APPROVAL, CLINICAL REVIEW, REJECT) combined with an independent PMS ALARM signal, aligned with US and EU regulatory guidance. The deployment decision taxonomy governs change-control determinations documentable in PCCP submissions; the ALARM signal addresses PMS obligations under EU MDR Article 83 and FDA PMS expectations. The composite output captures the critical REJECT + ALARM state, where no deployable new model is available and the released model is simultaneously assessed as at risk in the field, a governance condition unrepresentable in prior frameworks.
2. A domain-agnostic governance architecture comprising three modules (DARM, MMM, CDM) that are designed to support the operationalization of FDA PCCP requirements and EU AI Act Article 43(4) provisions. The infrastructure specifies monitoring requirements and decision logic; clinical context determines thresholds and metrics through configuration.
3. A fixed performance reference comparison mechanism implementing regulatory SE. New model iterations must satisfy acceptance criteria relative to fixed performance reference metrics. The infrastructure supports both deterministic thresholds and Confidence Interval (CI)-based equivalence testing.
4. Integration of the ML Cumulative Performance Score (MLcps)^12^ into governance workflows. This composite metric enables domain-specific weighting for unified performance assessment.
5. Demonstration across two heterogeneous domains: sepsis prediction (tabular time-series, Random Forest (RF)) and brain tumor segmentation (volumetric imaging, convolutional networks). The same modules and priority-ordered decision hierarchy accommodate both domains; the active decision categories and the specific Boolean conditions are configured per domain (Section “AEGIS instantiation”).

The clinical applications serve as demonstration vehicles. The underlying models are intentionally conventional; the contribution lies in the governance infrastructure. Scope of this work: the AEGIS drift score detects covariate (input-distribution) shift only; concept drift—changes in the feature–outcome relationship—is out of scope here and identified as a future extension (Section “Discussion”). This validation is a proof-of-concept that exercises the governance mechanism; it is not a clinical evaluation. Section “Results” presents results from both instantiations; Section “Discussion” discusses regulatory implications, limitations, and future directions; Section “Methods” details the AEGIS architecture and experimental setup. The clinical models are deliberately conventional and serve only to exercise the governance layer. The empirical validation of this framework is presented in the following section, while the complete technical architecture, decision logic, datasets, and statistical procedures are detailed in the Materials and Methods (Section “Methods”).

## Results

To evaluate the operational feasibility of the AEGIS framework, we instantiated the architecture in two distinct clinical modalities: tabular early-warning sepsis prediction and high-dimensional volumetric brain tumor segmentation. As detailed in Section “Methods”, for each retraining iteration, AEGIS automatically ingests new data, evaluates performance and drift, and issues a deterministic, four-category deployment decision for the new candidate model (APPROVE, CONDITIONAL APPROVAL, CLINICAL REVIEW, or REJECT). Crucially, this is coupled with a parallel, independent Post-Market Surveillance (PMS ALARM) signal evaluating the safety of the previously released model. The sepsis experiment (Section “Sepsis prediction”) is designed to demonstrate decision-space coverage through calibrated perturbations; it characterizes governance mechanism behavior, not real-world drift prevalence or frequency. We emphasize that confirming an engineered perturbation produces its intended decision verifies the correctness of the decision logic; it does not, on its own, establish detection performance on real, unplanned drift. The brain-tumor case study complements this by exercising the same modules and the same priority-ordered hierarchy, under a domain-specific decision configuration, on genuine distributional shift across imaging cohorts. The brain tumor case study (Section “Brain tumor segmentation”) derives governance decisions from genuine distributional shifts across imaging cohorts, providing a complementary naturalistic trajectory. Together, the two instantiations establish that AEGIS produces the correct decision under controlled conditions (sepsis) and under real distributional change (brain tumor), but neither constitutes epidemiological evidence of any drift type’s clinical prevalence.

### Sepsis prediction

AEGIS validation used the PhysioNet Sepsis Challenge 2019 data. The underlying clinical model governed in this instantiation is an RF classifier; the full experimental setup and hyperparameter configurations are detailed in Section “AEGIS instantiation”. Results demonstrate governance behavior, not clinical suitability. The Golden dataset (the static, held-out evaluation benchmark defined in Section “AEGIS architecture”) comprised 4067 samples from 4067 patients.

**Fig. 1:**
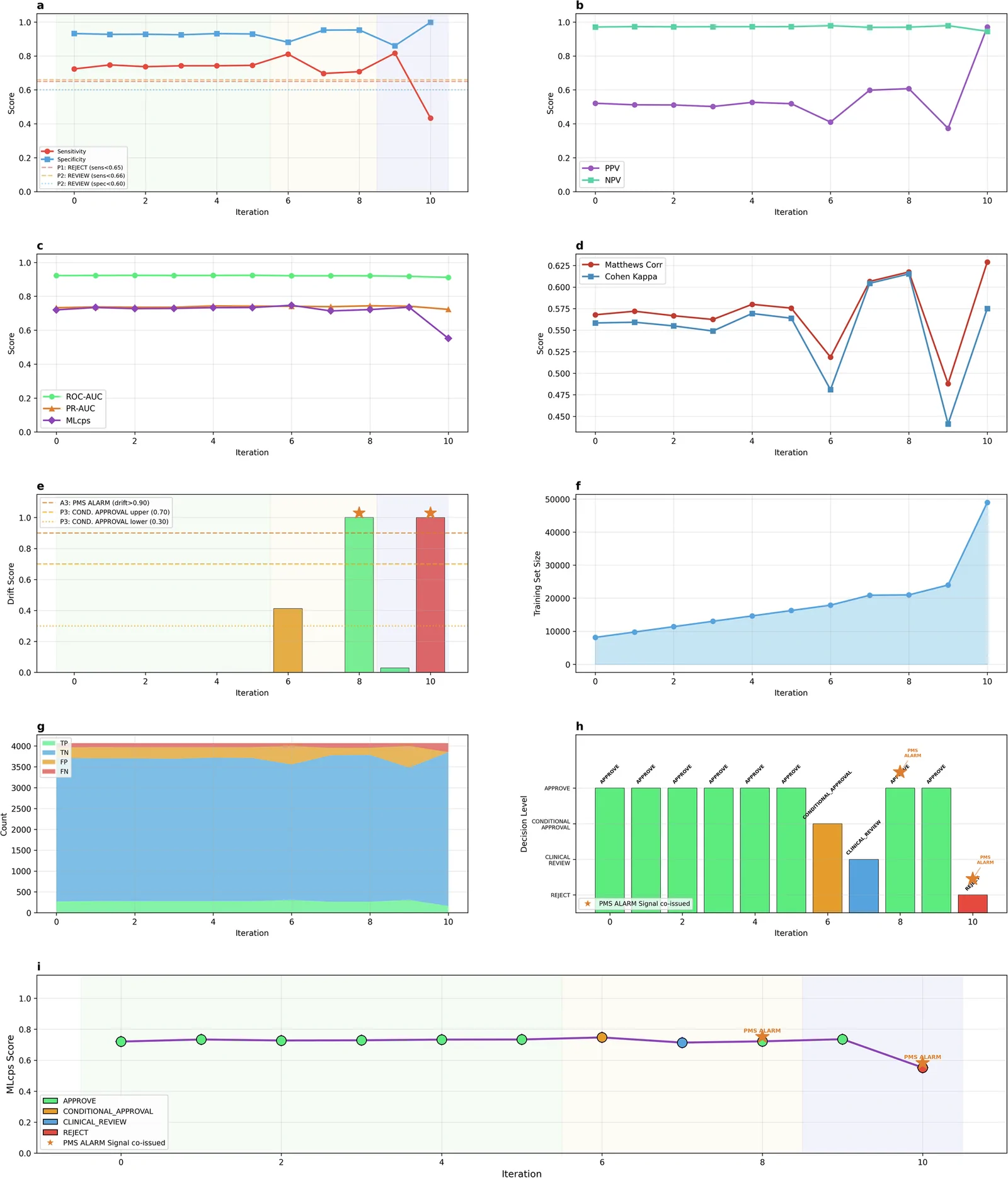
AEGIS comprehensive iterative change-control results for the sepsis-prediction instantiation, across iterations 0–10. **a** Clinical performance on the Golden set: sensitivity (red circles) and specificity (blue squares), with horizontal dashed reference lines at the REJECT safety floor (sensitivity 0.65) and the CLINICAL REVIEW thresholds (sensitivity 0.66, specificity 0.60). **b** Predictive values: PPV (purple circles) and NPV (teal squares). **c** Overall performance: ROC-AUC (green circles), PR-AUC (orange triangles), and MLcps (purple diamonds). **d** Agreement metrics: Matthews correlation coefficient (dark-red circles) and Cohen’s kappa (blue squares). **e** Drift score per iteration (Bonferroni-corrected Kolmogorov–Smirnov), with dashed reference lines at the minor-drift band (0.30 and 0.70) and the major-drift/PMS-ALARM threshold (0.90); each bar is colored by that iteration’s deployment decision. **f** Training-set size across iterations (shaded area). **g** Confusion-matrix components as a stacked area: true positives (green), true negatives (blue), false positives (orange), false negatives (red). **h** Deployment decisions on a four-level scale, bars colored by decision (green, APPROVE; orange, CONDITIONAL APPROVAL; blue, CLINICAL REVIEW; red, REJECT); an orange star marks iterations with a co-issued PMS ALARM (iterations 8 and 10). **i** MLcps score across iterations, each point colored by its decision using the same scheme, with orange stars marking co-issued PMS ALARM. Light background shading in panels **a**, **e**, and **i** denotes operational phases (stationary, drift, recovery).

Figure 1 shows results across 11 assessments (iterations 0–10).

The initial model (iteration 0) achieved sensitivity 0.723, specificity 0.933, Area Under the ROC Curve (ROC-AUC) 0.922. Iterations 1–5 (real data from Hospital A) maintained stable metrics with small fluctuations from retraining on progressively larger training sets: sensitivity ranged 0.742–0.753, specificity 0.926–0.933. The training dataset grew from 8134 to 16,268 samples across these iterations. Drift scores remained at 0.000, confirming distributional stability within the same hospital population.

Synthetic scenarios (iterations 6–10) exercised all four deployment decision categories and both ALARM composite states. Iteration 6 (cross-site data) produced a drift score of 0.412, triggering CONDITIONAL APPROVAL. Iteration 7 (clean data, but cross-site contamination in ${{\mathcal{D}}}_{T,k}$) caused sensitivity to drop to 0.702—above the Priority 2 hard threshold (0.66) but 0.021 below the fixed performance reference (*P*^ref^), triggering CLINICAL REVIEW via the fixed performance reference comparison mechanism (see Section “AEGIS architecture” for mechanism details). Iteration 8 (extreme feature scaling, *n* = 100) produced drift score 1.000, the small batch left the accumulated training pool intact so the new model passed all P4 checks (sensitivity 0.697), yielding APPROVE, while the PMS ALARM fired because the released model failed on the same extreme batch (APPROVE + ALARM). Iteration 9 (clean recovery batch) restored all metrics, yielding APPROVE. Iteration 10 (catastrophic label and feature corruption, *n* = 25,000) collapsed sensitivity to 0.428, triggering REJECT; the released model simultaneously failed on the same corrupt batch, firing ALARM (REJECT + ALARM, i.e., the critical composite state).

CDM deployment decisions: APPROVE (iterations 0–5, 8, and 9), CONDITIONAL APPROVAL (iteration 6), CLINICAL REVIEW (iteration 7), REJECT (iteration 10). PMS ALARM signals were co-issued at iteration 8 (APPROVE + ALARM: new model passed all deployment checks, but the released model was assessed as at risk from the major distributional shift) and iteration 10 (REJECT + ALARM: new model unsafe and released model simultaneously failing, i.e., the critical composite state). MLcps (sensitivity-weighted, computed via the official MLcps package, Section “AEGIS instantiation”) ranged from 0.7207 (iteration 0) to 0.7456 (iteration 6, peak) and collapsed to 0.5485 at iteration 10. ROC-AUC remained stable throughout (0.912–0.924).

Table 1 provides comprehensive results. The “Routing Trace” column in Table 1 traces the exact priority-ordered evaluation for the deployment decision at each iteration, serving as a concrete instantiation of the CDM logic formalized in the Methods (Section “AEGIS architecture”).

**Table 1:** Iteration summary with trigger conditions

Iter.	$| {{\mathcal{D}}}_{T,k}|$	Sens.	Spec.	AUC	MLcps	${{\mathcal{S}}}_{D}$	Trigger	Routing Trace^*^	Deploy. Decision	PMS Signal
0	8134	0.723	0.933	0.922	0.721	0.000	All satisfied	P1*✓* P2*✓* P3*✓* → P4	APPR.	—
1	9760	0.745	0.926	0.922	0.731	0.000	All satisfied	P1*✓* P2*✓* P3*✓* → P4	APPR.	—
2	11,386	0.745	0.926	0.923	0.731	0.000	All satisfied	P1*✓* P2*✓* P3*✓* → P4	APPR.	—
3	13,012	0.742	0.926	0.923	0.729	0.000	All satisfied	P1*✓* P2*✓* P3*✓* → P4	APPR.	—
4	14,638	0.742	0.932	0.923	0.733	0.000	All satisfied	P1*✓* P2*✓* P3*✓* → P4	APPR.	—
5	16,268	0.753	0.929	0.924	0.739	0.000	All satisfied	P1*✓* P2*✓* P3*✓* → P4	APPR.	—
6	17,894	0.809	0.881	0.922	0.746	0.412	minor drift ∈ [0.30, 0.70]	P1*✓* P2*✓* → P3	COND. APPR.	—
7	20,894	0.702	0.950	0.922	0.716	0.000	regression from *P*^ref^ = −0.021	P1*✓* → P2	CLIN. REV.	—
8	20,994	0.697	0.956	0.920	0.716	1.000	$0.90 \, < \,{{\mathcal{S}}}_{D}$	P1*✓* P2*✓* P3*✓* → P4	APPR.	ALARM
9	23,994	0.824	0.856	0.919	0.739	0.029	All satisfied	P1*✓* P2*✓* P3*✓* → P4	APPR.	—
10	48,994	0.428	0.999	0.912	0.549	1.000	sens. < 0.65; $0.90 \, < \,{{\mathcal{S}}}_{D}$	→ P1	REJ.	ALARM

The Routing Trace column traces the exact priority-ordered evaluation for the new candidate model at each iteration. The PMS Signal column records ﻿ALARM co-issuances where the released model was assessed as at risk.

*Iter.* iteration, *Sens.* sensitivity, *Spec.* specificity, *Deploy.* Deployment, *APPR.* APPROVE, *COND.* *APPR.* CONDITIONAL APPROVAL, *CLIN. REV.* CLINICAL REVIEW, *REJ.* REJECT.

Notations: $\left\vert {{\mathcal{D}}}_{T,k}\right\vert$ = training set size. ${{\mathcal{S}}}_{D}$ = Bonferroni-corrected Kolmogorov–Smirnov (KS) ﻿drift score.

*Priority notation (see Methods): P1 = Absolute Safety Failure; P2 = Performance Regression; P3 = Data Drift; P4 = Performance Improvement. (*✓* = check passed;  → = selected priority).

Note: *All thresholds are illustrative; production values require ISO 14971:2019 hazard analysis with documented traceability and/or alignment with published clinical performance benchmarks (see Supplementary S2.3)*.

A key observation: at iteration 8, the new candidate model received an APPROVE deployment decision (sensitivity 0.697, above all thresholds; the small *n*=100 batch did not corrupt the accumulated training pool), while a PMS ALARM was simultaneously co-issued because the released model’s performance on the same extreme batch collapsed and the drift score (1.000) exceeded the A3 threshold. This APPROVE + ALARM composite demonstrates two properties of AEGIS: (1) drift detection continues to operate as a *leading indicator*, identifying distributional risk before aggregate performance degradation is reflected in the new model; and (2) deployment and PMS monitoring are decoupled, i.e., the presence of field risk accelerates the case for deploying the new model rather than blocking it. This proactive approach aligns with FDA guidance on monitoring “reasonably foreseeable” changes.

A second observation: iterations 0–5 show small metric fluctuations (sensitivity 0.723–0.753) despite retraining on progressively larger training sets (8134 to 16,268 samples). This reflects the stable underlying distribution: batches are drawn from the same hospital population. Drift scores remain at 0.000 during this period, confirming distributional stability. The non-zero variability in sensitivity (unlike the previous implementation’s identical metrics) arises because the revised implementation unconditionally accumulates data and retrains at every iteration, correctly implementing the DARM protocol where ${{\mathcal{D}}}_{T,k+1}={{\mathcal{D}}}_{T,k}\cup {{\mathcal{D}}}_{D,k}$.

A third observation: the fixed performance reference comparison mechanism (iteration 7) detected a subtle sensitivity regression of 0.021 from the fixed performance reference (*P*^ref^ = 0.723). Although sensitivity (0.702) remained above the Priority 2 hard threshold (0.66), the fixed performance reference tolerance (*τ*^buf^ = 0.015) caught this clinically meaningful decline, demonstrating the two-tier threshold architecture: hard floors for safety, and fixed performance reference comparison for quality assurance.

SHapley Additive exPlanations (SHAP) analysis indicated top features related to vital-sign variability and laboratory markers. Across iterations the leading SHAP features were consistently Intensive Care Unit (ICU) length of stay and its variability and the last recorded temperature; the top-5 importance concentration was 0.285–0.336 across the governance lifecycle and the feature-ranking stability (Spearman correlation with the iteration-0 model) was 0.90–0.95 across stable iterations, with iteration 9 showing a slight dip to 0.898, and falling to 0.59 only at the corrupted REJECT iteration (iteration 10). Subgroup analysis showed small disparities: the maximum sensitivity gap across age tertiles and between sexes was ≤0.12 and the maximum specificity gap was ≤0.03; no fairness threshold was breached and no deployment decision was changed by a fairness condition. Per-iteration SHAP and subgroup values are tabulated in Supplementary Tables S27 and S29.

**Cross-site drift-detector operating characteristic**. The iterations above are *decision-space coverage* tests: each was constructed to elicit a specific decision and therefore verifies that the CDM priority hierarchy and ALARM mechanism route correctly, rather than establishing detection performance on unplanned drift. To assess the latter without any engineering, we evaluated the Bonferroni-corrected Kolmogorov–Smirnov (KS) drift score as a binary detector of *real* cross-hospital shift. Using a random Hospital A reference, we drew batches from the held-out, disjoint remainder of Hospital A (true no-shift, label 0) and from Hospital B (real cross-site shift, label 1), computing the identical drift score used by the CDM (same 34 monitored features and Bonferroni-corrected KS procedure; the 0.30 and 0.90 thresholds are those calibrated on Hospital A and were not tuned on Hospital B). Sampling is patient-level (one row per patient); we report 100 resampled batches per arm with 95% percentile-bootstrap confidence intervals (Table 2). The drift score distinguished same-hospital from cross-hospital batches with ROC-AUC rising from 0.82 (*n* = 25) to 1.00 (*n* ≥ 100). At the pre-specified minor-drift threshold (0.30) no false alarms were observed across the same-hospital batches, while detection of cross-site shift increased with batch size, reaching 1.00 at *n* ≥ 500. The major-drift threshold (0.90) was not reached by cross-site shift alone, consistent with reserving the ALARM signal for extreme distributional change. Perfect rank discrimination at *n* ≥ 100 did not imply threshold-level triggering at the 0.30 operating point, which emerged only for larger batches (≈500+); this informs a minimum batch-size (or evidence-accumulation) requirement for post-market monitoring.

**Table 2:** Cross-site drift-detector operating characteristic on real Hospital A vs. Hospital B covariate shift. A → A = same-hospital (null condition); A → B = cross-site shift

Batch size	ROC-AUC (95% CI)	TPR @0.30	FPR @0.30	TPR @0.90	mean ${{\mathcal{S}}}_{D}$ (A → A/A → B)
25	0.82 [0.77, 0.87]	0.00	0.00	0.00	0.001/0.039
50	0.99 [0.97, 1.00]	0.00	0.00	0.00	0.000/0.083
100	1.00 [1.00, 1.00]	0.00	0.00	0.00	0.001/0.153
200	1.00 [1.00, 1.00]	0.11	0.00	0.00	0.001/0.266
500	1.00 [1.00, 1.00]	1.00	0.00	0.00	0.001/0.396
1000	1.00 [1.00, 1.00]	1.00	0.00	0.00	0.002/0.538

100 resampled patient-level batches per arm; 95% percentile-bootstrap CIs; thresholds calibrated on Hospital A only.

### Brain tumor segmentation

The model trained on the full cohort established the “fixed performance reference” (*P*^ref^ = 0.726). To define the safety boundaries for iterative updates, we established a non-inferiority margin (*τ*^safe^ = 0.05), resulting in a deterministic acceptance threshold of *P*^fail^ = 0.676 ( = *P*^ref^ − *τ*^safe^).

During the initial stages of the lifecycle, the segmentation performance for iterations 1 and 2 remained below this threshold, triggering an automatic REJECT status. In iteration 3, while the model’s performance on the Golden set recovered to surpass the safety threshold, its performance on the drifting dataset declined relative to the previous step. This discrepancy produced an APPROVE + ALARM composite output: the deployment decision was APPROVE (Golden set above the deployment safety performance floor *P*^fail^), while the PMS ALARM was co-issued because the drifting set decline signaled a potential distribution shift in the newly assimilated batch warranting human adjudication and data forensic investigation. The first APPROVE-only release, without any concurrent field alert, occurred at iteration 4; here, the Dice Similarity Coefficient (DSC) on the Golden set not only exceeded the threshold but showed improvement over all prior iterations, corroborated by a simultaneous performance gain on the drifting set. In iteration 5, another APPROVE with co-issued ALARM occurred, consistent with the iteration-3 pattern (deployed but under field monitoring). Iterations 6 and 7 subsequently achieved clean APPROVE releases, with all governance conditions satisfied and no concurrent PMS signal. Following the clean approvals in iterations 6 and 7, the system’s performance began to degrade. Iteration 8 triggered a REJECT decision because the Golden set performance (*P*_*G*,8_ = 0.723) fell below the most recently released performance reference (${P}_{G}^{{\rm{rel}}}=0.742$). Most critically, iteration 9 produced a REJECT + ALARM composite output: the candidate model was rejected due to continued non-regression failure, while the PMS ALARM was simultaneously co-issued because performance on the drifting set declined relative to iteration 8 (*P*_*D*,9_ < *P*_*D*,8_), signaling an emergent distributional shift alongside the safety block. Iterations 10 to 12 similarly resulted in REJECT decisions as they failed to recover to the ${P}_{G}^{{\rm{rel}}}$ benchmark. Notably, iteration 11 was classified as a REJECT despite showing marginal gains over iteration 10 and significant improvement on the drifting set. This decision was governed by the PCCP logic: the Golden set performance (*P*_*G*,11_ = 0.707) remained approximately 3.5 DSC percentage points below the previously released performance reference at iteration 7 (${P}_{G}^{{\rm{rel}}}=0.742$), failing the requirement for sustained or improved performance relative to the last released performance reference. Per-iteration DSC values for the Tumor Core (TC) and the resulting decisions are given in Table 3; Table 4 maps the abstract AEGIS parameters to this instantiation.

**Table 3:** DSC metric for TC segmentation with routing trace

Iter.	$| {{\mathcal{D}}}_{T,k}|$	*P*_*G*,*k*_	*P*_*D*,*k*_	Trigger	Routing Trace*	Deploy. Decision	PMS Signal
0	1150	0.726 ± 0.313	—	fixed performance reference	—	—	—
1	200	0.646 ± 0.337	0.694 ± 0.325	safety violation (*P*_*G*,1_ < 0.676)	→ *P*1	REJ.	—
2	400	0.673 ± 0.337	0.748 ± 0.289	safety violation (*P*_*G*,2_ < 0.676)	→ *P*1	REJ.	—
3	600	0.690 ± 0.326	0.745 ± 0.308	minor drift (${P}_{G}^{{\rm{rel}}}=0.690$; *P*_*D*,3_ < *P*_*D*,2_)	*P*1*✓* → *P*4	APPR.	ALARM
4	800	0.700 ± 0.321	0.758 ± 0.284	All satisfied (${P}_{G}^{{\rm{rel}}}=0.700$)	*P*1*✓* → *P*4	APPR.	—
5	1000	0.725 ± 0.325	0.735 ± 0.325	minor drift (${P}_{G}^{{\rm{rel}}}=0.725$; *P*_*D*,5_ < *P*_*D*,4_)	*P*1*✓* → *P*4	APPR.	ALARM
6	1150	0.726 ± 0.313	0.836 ± 0.237	All satisfied (${P}_{G}^{{\rm{rel}}}=0.726$)	*P*1*✓* → *P*4	APPR.	—
7	1350	0.742 ± 0.308	0.892 ± 0.200	All satisfied (${P}_{G}^{{\rm{rel}}}=0.742$)	*P*1*✓* → *P*4	APPR.	—
8	1550	0.723 ± 0.311	0.910 ± 0.159	${P}_{G,8} < {P}_{G}^{{\rm{rel}}}$	→ *P*1	REJ.	—
9	1750	0.714 ± 0.308	0.900 ± 0.180	${P}_{G,9} < {P}_{G}^{{\rm{rel}}}$; *P*_*D*,9_ < *P*_*D*,8_	→ *P*1	REJ.	ALARM
10	1950	0.706 ± 0.318	0.904 ± 0.174	${P}_{G,10} < {P}_{G}^{{\rm{rel}}}$	→ *P*1	REJ.	—
11	2150	0.707 ± 0.322	0.931 ± 0.109	${P}_{G,11} < {P}_{G}^{{\rm{rel}}}$	→ *P*1	REJ.	—
12	2401	0.698 ± 0.327	—	${P}_{G,12} < {P}_{G}^{{\rm{rel}}}$	→ *P*1	REJ.	—

*Iter.* iteration, *APPR.* APPROVE, *REJ.* REJECT, *Deploy.* Deployment.

Notations: $\left\vert {{\mathcal{D}}}_{T,k}\right\vert$ = training set size; *P*_*G*,*k*_/*P*_*D*,*k*_ = candidate model performance on Golden/Drifting dataset at iteration *k*.

*Priority notation (see Methods) (*✓* = check passed;  → = selected priority).

**Table 4:** Mapping of abstract AEGIS parameters to brain tumor segmentation parameters

AEGIS Parameter	Concrete Value	Clinical Justification	CDM Decision
*P*^ref^	DSC: 0.726	fixed performance reference. Performance of the model trained on the full cohort on the Golden set (TC region)	—
*τ*^safe^	0.05	Defines *P*^fail^ boundary. Clinically acceptable margin below fixed performance reference	—
*P*^fail^ = *P*^ref^ − *τ*^safe^	0.676	Minimum acceptable TC segmentation for treatment planning	—
*C*^R^	*P*_*G*,*k*_ < *P*^fail^	Performance falls below the minimum requirement for safe radiotherapy and surgical planning	REJECT
*C*^NR^	${P}_{G}^{{\rm{rel}}} < {P}_{G,k}$	Non-regression condition: must match or exceed released performance reference	APPROVE
*C*^R^	${P}_{G,k} < {P}_{G}^{{\rm{rel}}}$	Regression condition: must be less than released performance reference	REJECT
*C*^R^	*P*_*D*,*k*_ < *P*_*D*,*k*−1_	Early warning for data quality issues in incoming batch	ALARM

Notations: *P*^ref^ = fixed performance reference; *τ*^safe^ = threshold defining the critical safety floor *P*^fail^; *P*^fail^ = deployment safety performance floor; *P*_*G*,*k*_/*P*_*D*,*k*_ = candidate model performance on Golden/Drifting dataset at iteration *k*; ${P}_{G}^{{\rm{rel}}}$ = most recently fully approved released model performance on Golden set; *C*^NR^/*C*^R^ = non-regression/regression condition.

*All thresholds are illustrative; production values require ISO 14971:2019 hazard analysis with documented traceability and/or alignment with published clinical performance benchmarks (see Supplementary S2.3)*.

## Discussion

At its core, AEGIS answers two questions independently at every iteration: 1) “Can the new candidate model be deployed?” and 2) “Is the currently released model still safe in the field?” The first is addressed by the deployment decision (P1–P4); the second by the parallel PMS ALARM signal (A1–A3).

This work presents AEGIS, a governance infrastructure for continuously learning medical AI systems. The modular architecture (DARM, MMM, CDM) provides manufacturers a structured approach to implementing PCCPs as defined by FDA guidance^13^. Demonstration across the PhysioNet Sepsis Challenge dataset (20,336 patients, 11 iterative deployment cycles) and the Brain Tumor Segmentation (BraTS) Glioma segmentation lifecycle (2601 subjects across post-treatment and pre-operative Glioma (GLI) cohorts) jointly established cross-domain generalizability and practical feasibility.

The experimental results demonstrate five key AEGIS capabilities (noting that these reflect governance mechanism behavior, not clinical validity):

1. Maintenance of predetermined performance thresholds across normal operating conditions (iterations 0–5: sensitivity 0.723–0.753, specificity 0.926–0.933, ROC-AUC 0.922–0.924)
2. Co-issuance of the PMS ALARM signal at iteration 8, where the released model was assessed as at risk from major distributional shift (drift = 1.000) even though the new candidate model passed all deployment checks (sensitivity 0.697). This demonstrates that the PMS monitoring channel operates independently of the deployment decision channel, and that distributional risk to the released model can be flagged without blocking deployment of an acceptable new model.
3. Appropriate REJECT decision when the deployment safety performance floor was violated (iteration 10: sensitivity 0.428 < 0.65)
4. The AEGIS’s capacity to distinguish between data distribution changes (triggering the PMS ALARM signal for investigation) and actual safety violations (triggering REJECT for the new candidate model)
5. Exercise of all four deployment decision categories (APPROVE, CONDITIONAL APPROVAL, CLINICAL REVIEW, REJECT) and both ALARM states (ALARM co-issued at iterations 8 and 10), including the REJECT + ALARM composite at iteration 10—the critical safety escalation state where no deployable new model is available and the released model is simultaneously at risk. This composite state, which requires regulatory escalation beyond standard rollback, is unrepresentable in prior single-hierarchy frameworks.

The brain tumor segmentation instantiation critically validates AEGIS’s capacity to govern high-dimensional spatial data, a primary challenge in adaptive radiology AI. Unlike the tabular sepsis model, this validation exercised governance logic against complex distributional shifts inherent in multi-parametric Magnetic Resonance Imaging (MRI), specifically the structural and morphological differences between pre-operative and post-treatment cohorts, providing a naturalistic, clinically motivated cross-cohort shift. An important regulatory finding occurred in iteration 3, where the system triggered an ALARM despite satisfactory Golden Set performance, correctly flagging a divergence in the incoming data stream that warranted investigation. Furthermore, the REJECT decision in iteration 11, driven by a failure to match the previously released performance reference (${P}_{G}^{{\rm{rel}}}$) rather than the absolute deployment safety performance floor, demonstrates the rigorous enforcement of the PCCP non-regression criteria. This illustrates how AEGIS is designed to flag potential “silent degradation” in continuous learning loops, by requiring that new iterations remain non-inferior, within tolerance, to the released reference ${P}_{G}^{{\rm{rel}}}$ (most recently fully approved released model performance on Golden set) rather than merely clearing minimum safety baselines. Taken together, the two instantiations provide complementary coverage of the decision taxonomy: the sepsis case study exercises all four deployment categories plus both ALARM composite states through calibrated synthetic scenarios, while the brain tumor case study demonstrates that the same modules and the same priority-ordered hierarchy, configured for a reduced two-category decision set, naturally produces APPROVE, APPROVE + ALARM, REJECT, and REJECT + ALARM decisions from real distributional shifts across imaging cohorts. Importantly, full taxonomy coverage is a property of the two instantiations *jointly*, i.e., no single instantiation is required to exercise every category, because which categories are active is a deliberate configuration decision tied to the clinical context. The two case studies together confirm both that all categories are reachable through appropriate configuration and that selective activation is a legitimate design practice.

The sepsis validation exercised all four deployment decision categories in the taxonomy (Section “Methods”): APPROVE, CONDITIONAL APPROVAL, CLINICAL REVIEW, and REJECT, plus ALARM composite states. The intermediate categories (CONDITIONAL APPROVAL via cross-site drift in iteration 6; CLINICAL REVIEW via regression from the fixed performance reference, *P*^ref^, in iteration 7) demonstrate that the two-tier threshold architecture—hard deployment safety performance floor values combined with fixed performance reference quality checks—provides graduated governance responses between full approval and rejection. This graduated response pattern aligns with FDA’s emphasis on monitoring “reasonably foreseeable risks” in PCCP design. These operational capabilities address gaps identified in prior governance frameworks, as summarized in Table 5.

**Table 5:** Comparison with existing approaches

Dimension	ABCDS (Duke)	FDA AI Lifecycle	Babic et al.	AEGIS
Focus	Institutional governance	Conceptual lifecycle	Surveillance gaps	Operational implementation
Contribution	Committee structure	Phase-based model	Policy recommendations	Metrics-driven logic
Decision Mechanism	Committee review	Phase guidance	Quarterly updates	Threshold-based; four deployment decision categories + independent parallel PMS’s ALARM signal
Monitoring	Periodic human review	Conceptual	Recommended	Continuous with drift detection
Drift Detection	Not specified	Conceptual	Recommended	KS-test with threshold
Regulatory Mapping	Implicit	General	FDA MAUDE	Explicit PCCP/MDR/AI Act

Several governance frameworks address different layers. The Algorithm-Based Clinical Decision Support (ABCDS) framework from Duke Health provides institutional oversight through committee governance^14^. It emphasizes human decision-making at milestones but does not specify automated metrics. The FDA’s AI Lifecycle concept offers phase-based guidance without implementation specifications^15^. Babic et al. identified MAUDE gaps and recommended quarterly updates on drift and stability, but acknowledged undefined technical infrastructure^1^.

AEGIS provides what we term *operational implementation*: concrete specifications (metrics, thresholds, decision logic, data protocols) translating high-level policies into executable procedures. Table 5 summarizes differentiators.

Because Table 5 is a qualitative comparison, we additionally report a component ablation to show that each element of the decision logic earns its place rather than reproducing a simpler rule. On the sepsis iterations, we compare the four nested configurations defined in Section “Robustness, sensitivity, and confidence analyses” (A0 safety floor only; A1 + performance buffer and fixed-reference comparison; A1b + minor-drift band; A2 full system + parallel ALARM). Each added component changes at least one iteration decision that the simpler configuration would not produce: minor-drift detection adds the CONDITIONAL APPROVAL at iteration 6; the fixed-reference check converts iteration 7 from APPROVE to CLINICAL REVIEW; and the parallel ALARM channel (with gold-standard deferral) produces the APPROVE + ALARM at iteration 8 (which the floor-plus-reference configuration would instead route to CLINICAL REVIEW) and the REJECT + ALARM at iteration 10. The floor-only baseline correctly rejects only the catastrophic iteration 10, missing the cross-site drift, the subtle regression, and both field-risk signals.

The approaches are complementary. An organization might adopt ABCDS for strategic oversight while deploying AEGIS for operational monitoring.

Table 6 maps Babic et al. recommendations to AEGIS implementation.

**Table 6:** Mapping Babic et al. recommendations to AEGIS

Recommendation	AEGIS Implementation
Quarterly deployment updates	DARM iterative cycle; Table 1 shows 11 tracked iterations
Concept drift detection	Partially addressed via proxy check (P2/A2 performance regression); direct detection is future work
Covariate shift monitoring	Golden dataset (${{\mathcal{D}}}_{G}$) static as reference; MMM KS-test on Drifting dataset ${{\mathcal{D}}}_{D,k}$
Algorithmic stability	fixed performance reference comparison (*τ*^buf^ = 0.015 absolute sensitivity)
Subgroup tracking	MMM bias score across demographics

While primary governance decisions use clinical performance metrics (sensitivity, specificity, ROC-AUC), the AEGIS architecture accommodates the full spectrum of Trustworthy AI requirements increasingly mandated by regulatory bodies. The AEGIS implementation demonstrates this extensibility through concurrent monitoring of: (1) explainability metrics derived from SHAP analysis, quantifying how concentrated predictive influence is among top features; (2) fairness metrics assessing performance parity across demographic subgroups; (3) calibration quality via Brier scores and the relationship between Positive Predictive Value (PPV)/Negative Predictive Value (NPV) and clinical prevalence; and (4) statistical robustness indicators including Matthews Correlation Coefficient and Cohen’s Kappa that are less sensitive to class imbalance than accuracy-based metrics.

For applications subject to EU AI Act transparency requirements (Article 13) or FDA guidance on clinical decision support transparency, manufacturers could formalize these dimensions as additional PCCP boundaries. For example, triggering CONDITIONAL APPROVAL when explainability scores drop below threshold (indicating the model’s reasoning has shifted), or ALARM when fairness metrics reveal emerging disparities across protected groups. The NPV threshold (≥0.90 in this demonstration) exemplifies clinically-grounded governance: for sepsis screening, high NPV ensures that patients receiving negative predictions can be confidently triaged to lower-acuity pathways. This multi-dimensional approach addresses regulatory expectations extending beyond traditional performance metrics.

**Scope Disclaimer:** The sepsis prediction and brain tumor segmentation applications serve exclusively as proof-of-concept demonstrations of governance infrastructure functionality. *No clinical evaluation or validation has been performed*, and the underlying predictive models are *not intended for clinical use*. Performance metrics reflect AEGIS’s behavior under controlled conditions, not clinical efficacy or safety. Clinical deployment would require independent validation studies, Institutional Review Board (IRB) approval, and regulatory clearance commensurate with the system’s intended use and risk classification. Thresholds, weights, and decision boundaries are illustrative; production implementations must derive parameters through formal clinical risk assessment.

Several additional limitations warrant acknowledgment.

First, synthetic drift scenarios (iterations 6–10), while useful for demonstrating all four deployment decision categories and ALARM composite states, may not fully capture real-world complexity from demographic changes, equipment variations, or clinical practice evolution. The cross-site scenario (iteration 6) uses real Hospital B data from the PhysioNet Challenge, providing more realistic distributional shift than purely synthetic perturbations, but future validation should incorporate documented real-world drift events.

Second, AEGIS relies on aggregate performance metrics that may not capture shifts in model uncertainty distribution. A model can maintain identical aggregate performance while dramatically shifting where it is uncertain. For ensemble-based systems, disagreement patterns between component models could provide additional governance signals; this extension represents a direction for future work.

Third, the current fairness assessment (bias score as sensitivity/specificity difference between demographic groups) is simplified. Production implementations should incorporate fairness evaluation across subgroups, consistent with the bias-mitigation and representativeness principles outlined in the FDA’s Good Machine Learning Practice (GMLP) guidance^10^.

Fourth, operational deployment considerations (computational resources, storage requirements for versioned datasets, human oversight burden) were not quantitatively evaluated. These factors significantly impact implementation feasibility.

Fifth, validation models do not represent State-Of-The-Art (SOTA) performance. This is intentional: demonstrating governance with conventional models establishes minimum functionality. Since AEGIS interacts with models only through outputs and standard metrics, no architectural dependency prevents application to sophisticated models.

Sixth, aggregate Key Performance Indicator (KPI) monitoring may not detect adversarial data poisoning attacks that preserve overall performance while corrupting specific subpopulations or decision boundaries^16^. Disagreement-based monitoring using temporal ensembles, comparing current models against historically validated versions, offers a promising detection mechanism^17^, though this approach requires validation in adversarial settings. The demonstrated detection delays when relying solely on performance metrics motivate the development of complementary security signals.

Seventh, although supplementary analyses (Tables S34 and S35) demonstrate metric extensibility by evaluating alternative regions and criteria (such as the Non-Enhancing Tumor Core (NETC) subregion using Normalized Surface Distance (NSD)), the primary brain tumor case study focuses on a single performance metric (DSC on TC) without concurrent fairness, explainability, or composite MLcps tracking, in contrast to the sepsis instantiation’s comprehensive multi-dimensional monitoring. This asymmetry reflects the proof-of-concept scope: the brain tumor instantiation demonstrates the AEGIS’s applicability to high-dimensional imaging data and its capacity to detect distribution shift through dual-dataset monitoring (Golden vs. Drifting). Future work should extend the brain tumor instantiation to include demographic subgroup analysis (e.g., tumor grade, patient age), explainability metrics appropriate for segmentation (e.g., saliency maps, uncertainty heatmaps), and a domain-appropriate MLcps configuration to achieve monitoring parity with the sepsis case study.

Eighth, this study is retrospective and simulation-based; no prospective, real-world deployment data were used. In the sepsis instantiation, features are aggregated over each patient’s entire ICU stay, so the task is retrospective rather than a true forward-in-time prediction setup; clinical deployment would require specifying the prediction time relative to admission, the available measurement window, and the label definition relative to prediction time.

Ninth, the DARM assumes high-confidence expert labels for assimilated data. Real-world label noise and annotation disagreement could degrade governance decisions and should be modeled explicitly (e.g., label-confidence weighting or adjudication thresholds) in production deployments.

Tenth, the drift score is computed per feature using Bonferroni-corrected two-sample KS tests and therefore does not capture joint or correlation-structure (multivariate) shifts; multivariate drift detection is left to future work.

Eleventh, concept drift (changes in the feature–outcome relationship) is out of scope. The current drift score quantifies covariate (input-distribution) shift only; direct concept-drift modeling, including explicit modeling of the feature–outcome relationship over time, is identified as a future extension.

Twelfth, the cross-site operating-characteristic analysis (Table 2) shows that reliable triggering at the pre-specified minor-drift threshold requires adequately sized batches (≈500+ patients); smaller post-market batches would need a lower threshold or accumulation of evidence across batches. The reported false-alarm rate of zero is an observation over the resampled same-hospital batches, not a guarantee that the true false-alarm probability is exactly zero.

The MLcps metric^12^ provides a principled approach to composite performance assessment by projecting multiple evaluation metrics onto a two-dimensional polar coordinate system. However, as noted by the original authors, the reliability of MLcps depends on the quality of constituent metrics and the appropriateness of assigned weights. In clinical deployment, weight assignments should be validated through published clinical performance benchmarks and formal risk assessment per ISO 14971:2019 rather than heuristic selection. The sensitivity-weighted configuration used in this demonstration (raw weights—Sensitivity: 1.5, ROC-AUC: 1.3, Balanced Accuracy: 1.1, Specificity: 1.0—normalized internally by the official MLcps package) reflects clinical priorities for sepsis detection; alternative applications may require substantially different weighting schemes derived through stakeholder consensus.

AEGIS is structured to align with the FDA’s PCCP components as specified in the August 2025 final guidance^4,13^: Description of Modifications (DARM protocols, CDM categories), Modification Protocol (MMM procedures), and Impact Assessment (fixed performance reference comparison, audit trails). The four-category deployment taxonomy combined with the parallel PMS ALARM signal is designed to support documentation of predetermined governance pathways in 510(k) submissions, with the ALARM channel addressing separate MDR PMS obligations. Whether a specific implementation satisfies submission requirements depends on device classification, intended use, and regulatory counsel.

For EU manufacturers, predetermined changes documented in technical documentation are designed to support qualification for AI Act exemptions from “substantial modification” requirements^9^. We stress, however, that the MDR remains the primary legal framework governing the medical device and that no EU instrument currently establishes a PCCP-equivalent pathway: AEGIS should therefore be understood as an internal governance and quality-management aid that helps structure MDR post-market surveillance and AI Act technical documentation, not as a substitute for conformity assessment. Manufacturers must satisfy both the MDR and the AI Act. Manufacturers should engage early with regulators to align parameters with expectations for specific device classifications.

The choice of two maximally different clinical domains, structured tabular data for sepsis prediction versus high-dimensional imaging data for segmentation, was designed to stress-test AEGIS generalizability. That the problem-specific instantiations from abstract DARM/MMM/CDM architectures handle both domains suggests applicability to other supervised learning tasks in healthcare, including natural language processing applications, signal processing (ECG, EEG), and multimodal fusion systems. Some key parameters for domain-specific adaptation or instantiation include threshold calibration (derived from clinical risk assessment) and MLcps weight adjustment (reflecting clinical priorities), among others, as summarized in Section “AEGIS instantiation”. However, generalizability of the governance infrastructure does not imply clinical validity of any specific model deployed within it; each clinical application requires independent validation appropriate to its intended use.

The supplementary analyses in S4.2 provide three additional dimensions of generalizability evidence. First, the Framework Compatibility Simulation (Table S33) shows that the multi-tier decision logic, including the intermediate CLINICAL REVIEW category and the parallel ALARM channel, executes unchanged on volumetric imaging data, indicating that category deactivation in the primary brain tumor protocol is a configuration choice, not a structural constraint. Second, the MMM extensibility analyses (Tables S34–S35) show that, *within the imaging instantiation*, the monitored metric can be changed without altering the governance implementation: separate experiments substituting Whole Tumor (WT) DSC and NETC NSD monitoring (metrics with substantially different distributional properties from TC DSC) required no change to the MMM or CDM code. This is metric-level reconfigurability within a single domain, and should not be read as a claim that the decision logic is identical across domains. Furthermore, sensitivity analyses (Table S32), which map a precise deployment transition boundary at *τ* ^reg^ = 0.019 for the imaging cohort (per-iteration static margins are given in Section “Brain tumor segmentation instantiation”; the reported boundary reflects the dynamic ${P}_{G}^{{\rm{rel}}}$ update rule, under which ${P}_{G}^{{\rm{rel}}}$ advances on each APPROVE so that the flip cascades through iterations 8–12 once iteration 8 is approved), illustrate how the framework’s governance parameters can be systematically calibrated to the distinct statistical variance and risk profiles of different clinical domains.

The current AEGIS implementation addresses natural drift, that is, distributional changes arising from demographic shifts, equipment upgrades, or evolving clinical practice, but does not explicitly address *adversarial* threats. Data poisoning attacks, wherein malicious or corrupted samples are introduced into post-market data streams, represent a foreseeable risk that regulators increasingly expect manufacturers to anticipate^16^. Critically, poisoning attacks can be designed to preserve aggregate performance metrics while degrading model behavior on targeted subpopulations or specific decision boundaries. This evasion of standard KPI monitoring renders conventional PMS inadequate for security-critical applications.

We propose extending the AEGIS’s monitoring layer to incorporate *disagreement-based* signals adapted from ensemble learning approaches^17^. Rather than relying solely on aggregate metrics, the extended AEGIS infrastructure would maintain previously validated model versions in a “shadow” or “sentinel” mode, continuously comparing their predictions against the current deployed model. Sudden increases in inter-version disagreement, particularly when localized to specific patient subgroups or feature regions, could serve as early indicators of data integrity compromise, thereby triggering ALARM or REJECT decisions before poisoned data propagates into training sets. This temporal ensemble approach leverages the observation that poisoning attacks designed to evade aggregate metrics typically cannot simultaneously preserve agreement patterns across model generations.

Integration with the existing CDM decision logic is straightforward: disagreement metrics would augment the current drift score as an additional input to the four-category deployment decision taxonomy and parallel PMS ALARM signal. However, the signal-to-action mapping requires careful calibration; disagreement spikes without corroborating adverse events might warrant enhanced monitoring rather than immediate rollback. The threshold configuration for disagreement-triggered governance decisions, like performance thresholds, should be derived through published clinical performance benchmarks and formal risk assessment per ISO 14971:2019 and documented in the PCCP submission. Validation of this security-aware extension against realistic poisoning scenarios represents an important direction for future work.

Future work should address several practical extensions. Incorporation of uncertainty quantification methods would strengthen confidence estimation for release decisions by providing per-prediction reliability indicators alongside aggregate metrics. Extension to handle concept drift (changes in the relationship between features and outcomes) in addition to covariate drift would enhance robustness, as the current infrastructure primarily detects distributional changes in input features rather than shifts in outcome relationships^18^.

For security-aware governance, empirical characterization of reference disagreement rates between model versions would help distinguish normal model evolution from anomalous divergence patterns indicative of data integrity issues. Formal mapping of disagreement signals to CDM decision categories, specifying when elevated disagreement should trigger CLINICAL REVIEW versus ALARM versus REJECT, represents essential work for operationalizing the temporal ensemble approach.

Development of standardized benchmark datasets and drift scenarios specifically designed for PCCP framework evaluation would facilitate reproducible comparison of governance approaches across the research community.

The regression tolerance sweep (Supplementary Table S32) identifies a critical phase transition at *τ*^reg^ ≈ 0.019 for Glioma DSC monitoring (per-iteration static margins are given in Section “Brain tumor segmentation instantiation”; the reported boundary reflects the dynamic ${P}_{G}^{{\rm{rel}}}$ update rule, under which ${P}_{G}^{{\rm{rel}}}$ advances on each APPROVE so that the flip cascades through iterations 8–12 once iteration 8 is approved), below which the rejection sequence (iterations 8–12) is stable, and above which these models transition to APPROVE. This empirical boundary provides actionable guidance for future implementers: future work should formalize a standardized, risk-based method for deriving *τ*^reg^ from ISO 14971:2019 hazard severity analyses, thereby creating a robust bridge between observed model variance and the PCCP’s requirement for pre-specified, clinically justified governance parameters.

## Methods

This section presents AEGIS in two parts: (1) AEGIS Architecture, describing domain-agnostic governance components, and (2) AEGIS Instantiation, demonstrating configuration for specific clinical domains.

### Regulatory context and design rationale

Two regulatory mandates drive AEGIS design: auditable change control and rigorous PMS for continuously learning models.

The PCCP requires manufacturers to define, a priori, the criteria under which models may be modified and the acceptable limits of performance change. This necessitates a formal gate that objectively determines whether an update requires a new submission or falls within pre-authorized scope^13^.

The EU MDR 2017/745 demands comprehensive PMS and clinical follow-up. For adaptive models, this means continuously monitoring real-world performance^19^, demonstrating that new data does not introduce unacceptable bias^10^, and ensuring safety claims remain valid across releases.

The EU AI Act emphasizes quality management, data governance, and robustness for high-risk applications. It requires continuous monitoring and documented control of changes to system logic or data distributions^8^.

Two clarifications frame the scope of these claims. First, in the EU the MDR remains the primary legal framework for conformity assessment, clinical evaluation, post-market surveillance, and vigilance; the AI Act is an additional horizontal layer and does not replace it. ISO 14971:2019 is the risk-management standard that informs threshold derivation, not itself a PCCP instrument, and at present no EU instrument provides a PCCP-equivalent pathway. Second, AEGIS is an internal governance, quality-management, and post-market-monitoring infrastructure that can *support* the preparation and execution of a PCCP and of MDR/AI Act post-market obligations; the regulatory mappings in this paper are *operational analogies* intended to guide documentation structure, and are not claims of regulatory equivalence. Definitive regulatory classification depends on device-specific intended use and risk class and should be confirmed with regulatory counsel.

These frameworks differ in emphasis. The PCCP focuses on pre-defining acceptable changes; the MDR emphasizes ongoing risk assessment; the AI Act mandates extensive documentation. Table 7 summarizes the philosophical differences between FDA and EU approaches.

**Table 7:** Regulatory philosophy comparison: FDA vs. EU MDR

Dimension	FDA (510(k)/PCCP)	EU MDR
Core Philosophy	SE	Continuous risk management
Foundational Question	“Is this device as safe and effective as something already proven?”	“Does the ongoing risk-benefit analysis remain acceptable?”
Evidence Paradigm	Comparative (new vs. predicate)	Cumulative (ongoing real-world evidence)
Clinical Data Focus	Premarket; 510(k) may rely on bench testing	Continuous clinical evaluation (PMCF)
Change Control Logic	Modifications within PCCP bounds maintain equivalence	Changes assessed against evolving risk profile
Methodological Backbone	Predicate device comparison	ISO 14971:2019 risk management

*PMCF* Post-Market Clinical Follow-up.

The FDA grounds safety determinations in SE: devices gain clearance by demonstrating comparability to legally marketed predicates. The PCCP extends this philosophy to post-market modifications. In contrast, the MDR anchors to continuous risk management informed by ISO 14971:2019, asking whether ongoing risk-benefit analysis remains acceptable throughout the lifecycle. We emphasize that ISO 14971:2019 is a risk-management standard rather than a change-control or PCCP instrument; the MDR does not currently provide a PCCP-equivalent mechanism, so the feasibility of a formal PCCP for medical devices in the EU remains an open question.

AEGIS addresses both paradigms simultaneously. The fixed performance reference comparison mechanism is designed to support FDA equivalence requirements. The MMM’s continuous monitoring is structured to align with MDR surveillance mandates. The four-category deployment taxonomy combined with an independent PMS ALARM signal maps to both structures.

### AEGIS architecture

AEGIS defines governance architecture through three modules. This architecture specifies *what* must be monitored and *how* decisions must be made. Domain-specific adaptation requires only threshold calibration and metric weighting, not architectural changes.

AEGIS maps directly to the FDA PCCP components as defined in the August 2025 final guidance^4,13^. The *Description of Modifications* is addressed through DARM protocols and CDM decision categories. The *Modification Protocol* is realized through MMM metric procedures. The *Impact Assessment* is operationalized through fixed performance reference comparison.

#### Dataset Assimilation and Retraining Module (DARM)

In geosciences, data assimilation integrates new observations with numerical models to refine system estimates^20^. We adapt this concept for ML governance. The DARM manages controlled ingestion and structured integration of new observations, assigning definitive status to every data point. Before these newly ingested observations can be assigned to their respective dataset splits, they must first undergo rigorous quality control and high-confidence annotation to establish an accurate and auditable clinical ground truth. *Label Governance and Expert Review.* Label provenance and adjudication are governed within the quality management system. Incoming cases are labeled using pre-specified criteria. A subset undergoes expert adjudication with documented disagreement resolution. Cases flagged as uncertain or out-of-distribution are excluded until resolved. Each batch is versioned with provenance metadata (source, collection window, labeling method, reviewer identifiers, timestamps).

This mechanism ensures dataset integrity (strict separation between training, validation, and evaluation data) and transparency (auditable logs of collection timing and allocation). Three dataset splits are maintained at each iteration:

*Training Dataset*. The Training dataset updates model parameters at each iteration. This dynamic set comprises examples reviewed by expert clinicians with high label confidence. At iteration *k*, the model trains exclusively on ${{\mathcal{D}}}_{T,k}$.

*Golden Dataset*. The Golden dataset (${{\mathcal{D}}}_{G}$) is a static, held-out benchmark for evaluating generalization across all iterations. It is never used for training, validation, or tuning. This guarantees a consistent, unbiased comparison across iterations.

*Drifting Dataset*. The Drifting dataset (${{\mathcal{D}}}_{D,k}$) is dynamic and serves two functions at each iteration *k*:

- Drift Testing: ${{\mathcal{D}}}_{D,k}$ tests the current model internally. Performance on this set is monitored for distribution changes or feature decay.
- Subsequent Training Pool: Upon successful testing and expert review, ${{\mathcal{D}}}_{D,k}$ contents integrate into ${{\mathcal{D}}}_{T,k}$ for iteration *k* + 1, enabling continuous learning.

The DARM addresses EU AI Act data governance requirements, MDR evidence needs, and PCCP control over retraining data. By assigning status to every observation, it provides auditable logs preventing evaluation set contamination.

**Fig. 2:**
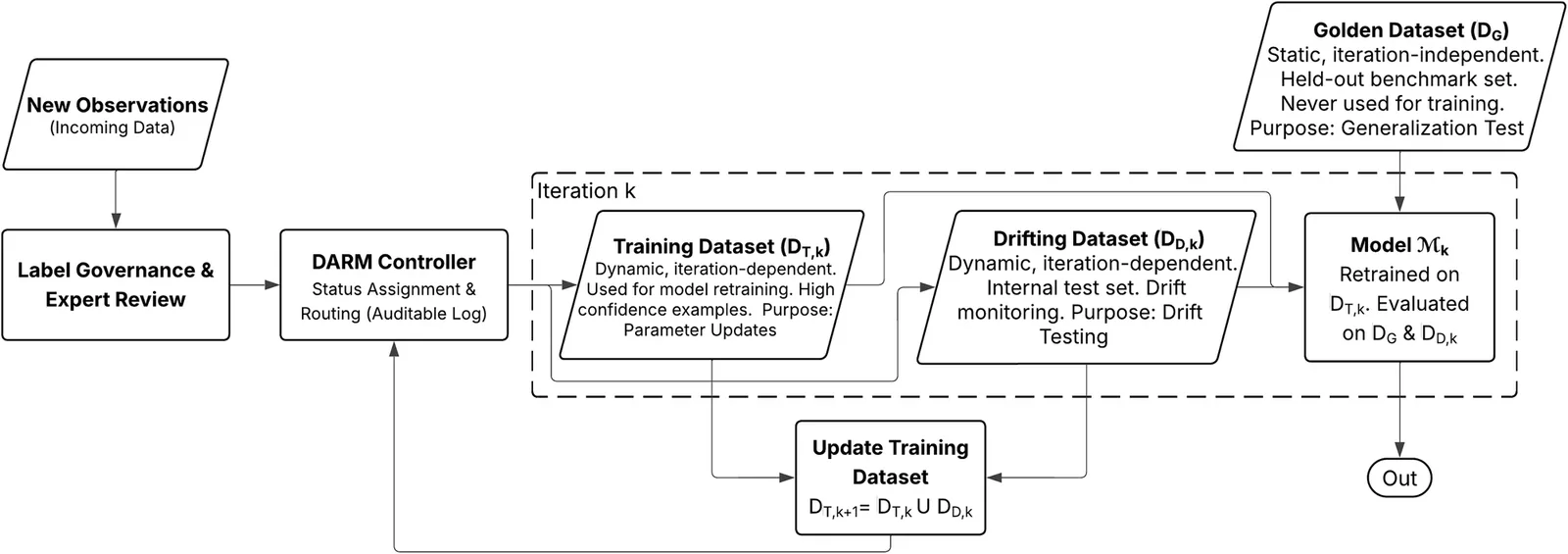
DARM architecture. The DARM manages the lifecycle and routing of three distinct dataset splits. The Training dataset (${{\mathcal{D}}}_{T,k}$) is used for dynamic parameter updates at each iteration. The Golden dataset (${{\mathcal{D}}}_{G}$) serves as a static, iteration-independent benchmark used exclusively for final generalization testing. The Drifting dataset (${{\mathcal{D}}}_{D,k}$) functions as a dynamic internal test set for real-time drift monitoring; upon successful verification and expert review, its contents are integrated into the training pool for the subsequent iteration.

Fig. 2 illustrates the DARM architecture, showing dataset interactions and the feedback loop where ${{\mathcal{D}}}_{D,k}$ integrates into ${{\mathcal{D}}}_{T,k+1}$.

#### Model monitoring module (MMM)

The MMM calculates metrics on dataset splits and maintains historical records of KPIs. Table 8 summarizes required parameter categories.

**Table 8:** Parameters

Parameter categories	Description
Tolerance	Acceptable deviation from target value
Range	Minimum and maximum bounds for acceptable values
Reference	Reference values against which comparisons are made
Distribution Shift Metrics	KL divergence, JS distance, mean drift
TAI Metrics	Demographic parity, equalized odds metrics
Model Performance Metrics	Accuracy, sensitivity, specificity, AUC, F1-score

*TAI* Trustworthy AI.

The constant values (tolerance, range, and reference) are derived during PCCP preparation from published clinical performance benchmarks and formal risk assessment per ISO 14971:2019.

**Fig. 3:**
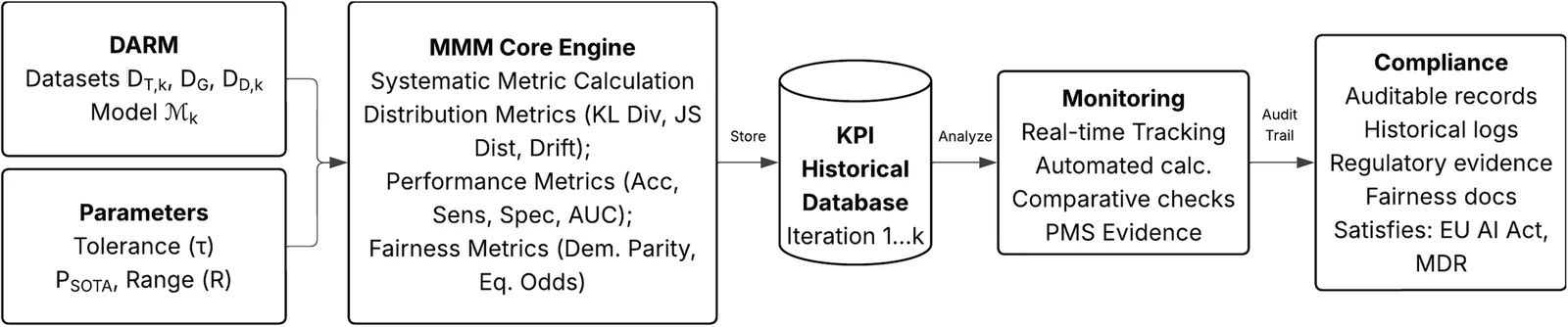
MMM architecture. The module systematically calculates and tracks metrics across three core categories: performance, fairness, and distribution. Historical KPIs are maintained in a central database to support real-time tracking, automated comparative checks, and PMS evidence. The infrastructure generates auditable records, logs, and compliance documentation, ensuring the system satisfies EU AI Act and MDR requirements.

The MMM provides unbiased evidence for continuous monitoring and drift detection by calculating performance metrics on both static and dynamic sets from the DARM. Figure 3 illustrates the architecture.

#### Conditional Decision Module (CDM)

The CDM produces a composite output at each iteration: (1) a deployment decision (APPROVE, CONDITIONAL APPROVAL, CLINICAL REVIEW, REJECT) governing the *new candidate model*, and (2) a PMS ALARM signal assessing the continued safety of the *currently released model already in use*. These two outputs are evaluated independently against separate metric sets and may be combined, which is why APPROVE + ALARM is not contradictory: it means the new model is ready to deploy while the old model is simultaneously showing signs of risk in the field.

To formalize the decision logic, we first define a set of evaluation conditions. Let *C*_*i*_ denote condition *i*, for *i* = 1, …, *N*, typically comparing a performance metric with a predefined threshold. The decision process can then be expressed using a ternary operator defined in Eq. (1):

1 $$D=f({C}_{1},{C}_{2},\ldots ,{C}_{N})?{D}_{1}:{D}_{2},$$

where *f* is an aggregated Boolean function. If *f* evaluates to *True*, decision *D*_1_ is selected; otherwise, *D*_2_. The formulation presented in Eq. (1) supports arbitrary logical combinations. For example, for *D*_1_ = “APPROVE” and *D*_2_ = “OTHER”, one may define *f* as (*C*_1_ ∧ *C*_2_ ∧ *C*_3_), where *C*_1_ = (*P*_*G*,*k*_≥*P*^ref^ − *τ*^safe^) tests proximity to optimal performance, *C*_2_ = (*P*_*G*,*k*_≥*P*_*G*,*k*−1_) tests non-regression, and *C*_3_ = (*P*_*G*,*k*_ ∈ *R*_*G*_) ∧ (*P*_*D*,*k*_ ∈ *R*_*D*_) tests cross-split stability. Complete specifications, including decision logic pseudo-code and threshold configuration templates, appear in Supplementary Sections S2.6 and S3.3.

Table 9 shows example decision configurations for the four deployment categories and the PMS ALARM signal. Each row in Table 9 expresses the aggregated Boolean condition that characterizes one decision category or signal.

**Table 9:** CDM decision categories

Decision	Question answered	Boolean Function f	Operator & Rationale
CHANGE-CONTROL DEPLOYMENT DECISIONS: new candidate model			
REJECT	Is new model safe?	(*P*_*G*,*k*_ < *P*^fail^) ∨ (any safety metric < its floor)	OR—any single safety failure blocks deployment
CLINICAL REVIEW	Does new model need human validation?	(*P*^fail^≤*P*_*G*,*k*_ < *P*^fail^ + *τ*^buf^) ∨ (*τ*^buf^ < *P*^ref^ − *P*_*G*,*k*_)	OR—any metric in buffer zone, or any regression from *P*^ref^ (fixed performance reference comparison fails).
CONDITIONAL APPROVAL	Does the new model need enhanced monitoring?	$({{\mathcal{S}}}_{D}\in {{\mathcal{S}}}_{D}^{{\rm{minor}}})\vee ({{\mathcal{S}}}_{T} < {{\mathcal{S}}}_{{\rm{TAI}}}^{{\rm{thr}}})$	OR—minor drift or any TAI concern each independently warrant enhanced monitoring
APPROVE	Does new model pass all checks?	$(({P}^{{\rm{ref}}}-{\tau }^{{\rm{buf}}})\le {P}_{G,k})\wedge ({P}_{G,k-1}\le {P}_{G,k})\wedge ({{\mathcal{S}}}_{D}\le {{\mathcal{S}}}_{D}^{{\rm{minor}}})\wedge ({{\mathcal{S}}}_{{\rm{TAI}}}^{{\rm{thr}}}\le {{\mathcal{S}}}_{T})$	AND—all checks must pass; any single failure routes to a lower category
PMS ALARM SIGNAL: currently released model			
ALARM	Is the released model still safe in the field?	$({P}_{D}^{{\rm{rel}}}\le {{P}^{{\rm{PMS}}}}^{* })\vee ({P}_{D}^{{\rm{rel}}}\le {P}_{G}^{{\rm{rel}}}-{\tau }^{{\rm{PMS}}})\vee ({{\mathcal{S}}}_{D}\in {{\mathcal{S}}}_{D}^{{\rm{major}}})$	OR—released model *P*^PMS^ (PMS safety performance floor) breached, or released model regressed from released performance reference, or major drift threatens released model validity.

Deployment decisions: Four decision categories for a new-model deployment. PMS ALARM signal: ALARM for the currently released model. The two parts are evaluated independently and produce a composite output.

Notations: *P*_*G*,*k*_ = candidate model performance on Golden dataset at iteration *k*; *P*^fail^/*P*^PMS^ = deployment/PMS safety performance floor below which REJECT/ALARM is triggered; *τ*^buf^ = threshold defining the performance buffer zone; *τ*^PMS^ = regression tolerance for the already released model; ${{\mathcal{S}}}_{D}$ = drift score; ${{\mathcal{S}}}_{D}^{{\rm{minor}}}/{{\mathcal{S}}}_{D}^{{\rm{major}}}$ = range for minor/major ${{\mathcal{S}}}_{D}$; ${\mathcal{S}}_{T}={TAI}$ composite score; ${{\mathcal{S}}}_{{\rm{TAI}}}^{{\rm{thr}}}$ = threshold for ${{\mathcal{S}}}_{T}$; *P*^ref^ = fixed performance reference; ${P}_{G}^{{\rm{rel}}}/{P}_{D}^{{\rm{rel}}}$ = fully approved released model performance on Golden/Drifting set.

^*^Production implementations may set *P*^PMS^ > *P*^fail^ to provide an early field warning before the deployment rejection threshold is reached; the two thresholds serve different governance functions and are evaluated on different models against different datasets.

All thresholds are illustrative; production values require ISO 14971:2019 hazard analysis with documented traceability and/or alignment with published clinical performance benchmarks (see Supplementary S2.3).

Escalation decisions (from REJECT to CONDITIONAL APPROVAL) use failure-trigger conditions that evaluate to *True* when a failure occurs; the natural aggregation operator is therefore logical disjunction (∨), ensuring a response is triggered by any single failing condition. For an APPROVE decision, by contrast, conditions evaluate to *True* when no failure is detected (i.e., pass-trigger conditions), making logical conjunction (∧) the appropriate operator: all checks must pass simultaneously to trigger approval.

When conditions for multiple categories are satisfied simultaneously, the priority-ordered hierarchy in Table 10 determines which decision is returned. The two tables are complementary: Table 9 defines the logical structure of each category; Table 10 defines the resolution order across categories.

**Table 10:** Decision hierarchy: priority-ordered evaluation

Priority	Check	Condition	Decision
New Model Deployment Hierarchy (evaluate in priority order on ${{\mathcal{D}}}_{G}$ and ${{\mathcal{D}}}_{D,k}$)			
P1	deployment safety performance floor	*P*_*G*,*k*_ < *P*^fail^	REJECT
P2	performance buffer/regression from fixed performance reference	(*P*_*G*,*k*_ ∈ *R*_*G*_) ∨ (*τ*^buf^ < *P*^ref^ − *P*_*G*,*k*_)	CLINICAL REVIEW
P3	minor drift/TAI	$({{\mathcal{S}}}_{D}\in {{\mathcal{S}}}_{D}^{{\rm{minor}}})\vee ({{\mathcal{S}}}_{T} < {{\mathcal{S}}}_{{\rm{TAI}}}^{{\rm{thr}}})$	CONDITIONAL APPROVAL
P4	all pass	all checks passed	APPROVE
PMS ALARM Signal (evaluate in parallel using released model on ${{\mathcal{D}}}_{D,k}$)			
A1	PMS safety performance floor	${P}_{D}^{{\rm{rel}}} < {P}^{{\rm{PMS}}}$	ALARM
A2	released model regression	${P}_{D}^{{\rm{rel}}}\le {P}_{G}^{{\rm{rel}}}-{\tau }^{{\rm{PMS}}}$	ALARM
A3	major distributional shift	${{\mathcal{S}}}_{D}\in {{\mathcal{S}}}_{D}^{{\rm{major}}}$	ALARM

Notations: ${{\mathcal{D}}}_{D,k}$ = Drifting dataset at iteration $k;{{\mathcal{D}}}_{G}$ = Golden dataset; ${P}_{D}^{{\rm{rel}}}$ = most recently fully approved released model performance on Drifting set; ${{\mathcal{S}}}_{D}$ = ﻿drift score; ${{\mathcal{S}}}_{D}^{{\rm{minor}}}/{{\mathcal{S}}}_{D}^{{\rm{major}}}$ = range for minor/major ${{\mathcal{S}}}_{D}$; ${{\mathcal{S}}}_{T}$ = TAI composite score; ${{\mathcal{S}}}_{{\rm{TAI}}}^{{\rm{thr}}}$ = threshold for ${{\mathcal{S}}}_{T}$.

Note I: The deployment decision and ALARM signal run in parallel; the composite output is (deployment decision) or (deployment decision + ALARM) when any of A1–A3 fires.

Note II: All threshold parameters (*P*^fail^, *P*^PMS^, *τ*^buf^, *τ*^PMS^, ${{\mathcal{S}}}_{D}^{{\rm{minor}}}$, ${{\mathcal{S}}}_{D}^{{\rm{major}}}$, ${{\mathcal{S}}}_{{\rm{TAI}}}^{{\rm{thr}}}$) are instantiation-defined PCCP parameters specified during deployment configuration per ISO 14971:2019 risk assessment. See Table 13 (sepsis) and Table 4 (BraTS) for domain-specific value examples.

Condition weighting is managed through threshold adjustment. Critical conditions (e.g., minimum sensitivity) require conservative thresholds reflecting low failure tolerance. Threshold specification must trace to ISO 14971:2019 risk management principles^21^.

${P}_{G}^{{\rm{rel}}}$ **update rule**. The most recently fully approved released model performance on Golden set (${P}_{G}^{{\rm{rel}}}$ in Table 9) is updated exclusively on APPROVE decisions. During periods of CONDITIONAL APPROVAL or CLINICAL REVIEW, ${P}_{G}^{{\rm{rel}}}$ remains anchored to the most recent full APPROVE, regardless of which model is currently deployed. This ensures that the regression check from ${P}_{G}^{{\rm{rel}}}$ is always evaluated against a fully validated reference, not against a tentative deployment.

${P}_{G}^{{\rm{rel}}}$ *vs* ${P}_{D}^{{\rm{rel}}}$ **distinction**. ${P}_{G}^{{\rm{rel}}}$ is the most recently fully approved released model performance on Golden set, while ${P}_{D}^{{\rm{rel}}}$ is the most recently fully approved released model performance on Drifting set. The PMS ALARM signal fires when the gap between these two quantities exceeds a tolerance *τ*^PMS^.

*P*^PMS^ *vs* *P*^fail^ **distinction**. *P*^PMS^ is the PMS safety performance floor below which ALARM is triggered, while *P*^fail^ is the deployment safety performance floor below which REJECT is triggered. *P*^fail^ governs the new candidate model evaluated on the Golden dataset ${{\mathcal{D}}}_{G}$; *P*^PMS^ governs the currently fully approved released model evaluated on the incoming drifting batch ${{\mathcal{D}}}_{D,k}$. Both are pre-specified in the PCCP, but they address different models, different datasets, and different governance questions. Setting *P*^fail^ < *P*^PMS^ in production creates a tiered safety architecture in which field risk is flagged before a formal deployment rejection threshold is reached.

**Decision confidence**. We define confidence as the bootstrap-estimated probability that an update satisfies acceptance criteria. For iteration *k*, we bootstrap evaluation sets *B* times, recompute metrics and conditions for each replicate, and define confidence as the fraction yielding the same outcome as the point estimate (the operational bootstrap procedure is detailed in Section “Robustness, sensitivity, and confidence analyses”). In this deterministic proof-of-concept, decision confidence is computed for completeness but is *not* used as a gating criterion for any deployment decision; production deployments with smaller post-market samples should adopt it (or the CI-based equivalence test) as an explicit acceptance gate. The bootstrap decision confidence (*B* = 1000) is high for the stable decisions (0.93–1.00 for APPROVE iterations 1–5, 8, and 9; 0.75 for iteration 0, the reference-establishment iteration; 1.00 for both the CONDITIONAL APPROVAL at iteration 6 and the REJECT at iteration 10) and is lowest (0.59) at iteration 7, the genuinely borderline CLINICAL REVIEW case, correctly reflecting its proximity to the fixed-reference tolerance.

**Fig. 4:**
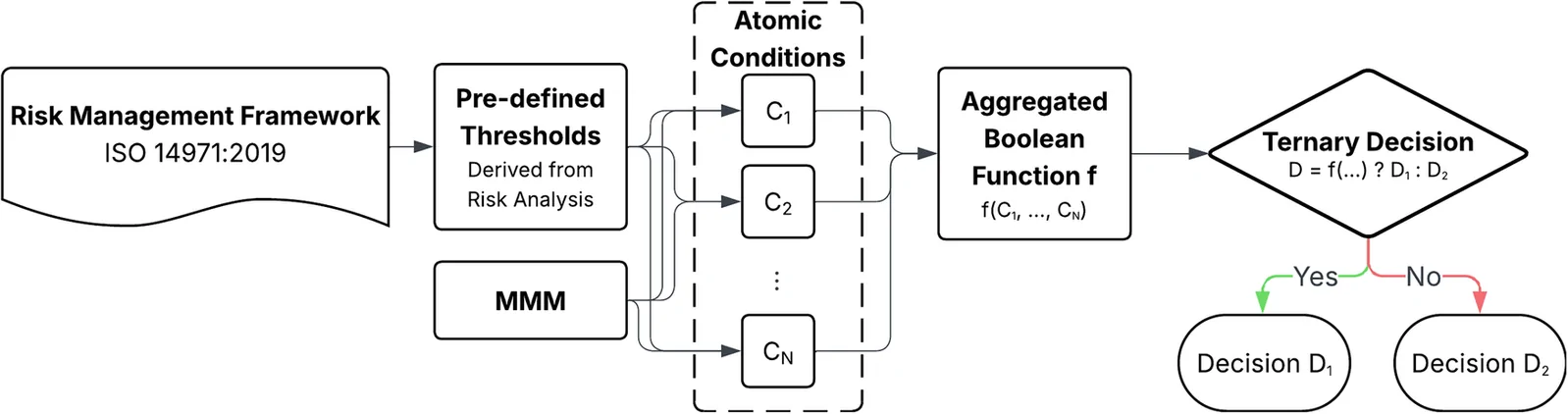
CDM architecture. Pre-defined thresholds derived from the risk-management framework (ISO 14971:2019) and the risk analysis, together with live metrics from the MMM, are evaluated as atomic conditions (*C*_1_,…,*C*_*N*_); a Boolean aggregation function combines them and a ternary rule selects the outcome. The branch labeled “Yes” (drawn in green) leads to decision outcome *D*_1_, and the branch labeled “No” (drawn in red) leads to decision outcome *D*_2_. Rectangular boxes denote inputs and processing steps, the dashed box groups the atomic conditions, and the diamond denotes the ternary decision.

Figure 4 illustrates the CDM architecture.

#### Fixed performance reference comparison mechanism

Threshold values (e.g., ${P}^{{\rm{fail}}},{\tau }^{{\rm{safe}}},{\tau }^{{\rm{buf}}},{\tau }^{\,{\rm{reg}}},{\tau }^{{\rm{PMS}}},{{\mathcal{S}}}_{{\rm{TAI}}}^{{\rm{thr}}}$) are derived during PCCP preparation from published clinical performance benchmarks and formal risk assessment per ISO 14971:2019. After the first APPROVE decision, AEGIS records the model’s measured performance on the Golden dataset as the fixed performance reference (*P*^ref^), which is an internally measured post-approval value; its numerical magnitude is informed by, but not identical to, any pre-specified external bound. *P*^ref^ serves as the invariant operational reference in subsequent CDM evaluations using acceptance criteria described below.

In a PCCP context, *P*^ref^ is derived from data available at the time of the device’s initial regulatory-authorized release. Alternatively, it may be drawn from externally established benchmarks such as validated clinical performance standards or published reference values, provided these are available and consistent with the clinical task and dataset characteristics. In simulation settings where real deployment data is unavailable, *P*^ref^ may be computed using the full available cohort, as is the case in the brain tumor segmentation instantiation presented in Section “AEGIS instantiation”.

#### Statistical acceptance criteria

AEGIS supports two approaches for evaluating acceptable performance.

*Deterministic threshold comparison.* Point estimates are compared directly against thresholds. For each metric *m* ∈ {sensitivity, specificity, ROC-AUC}, the update is accepted if *m*_*k*_≥*m*_threshold_. This approach suits large evaluation datasets where point estimates have low variance. For production deployments with smaller post-market samples, where point estimates carry greater variance, CI-based non-inferiority testing replaces the deterministic comparisons. The deterministic approach used throughout this proof-of-concept is appropriate given the large evaluation dataset; CI-based testing is recommended when post-market sample size falls below approximately 500, where bootstrap resampling provides materially stronger guarantees against type I acceptance errors.

*CI-based equivalence testing (recommended for production).* For smaller post-market samples, CI testing provides stronger guarantees. Let Δ*m*_*k*_ = *m*_*k*_ − *m*_0_ denote the difference between current and reference performance. Using bootstrap resampling, we compute a (1 − *α*) CI. The update is **non-inferior** if ${{\rm{CI}}}_{\Delta {m}_{k}}^{{\rm{lower}}}\ge -{\delta }_{m}$, or **equivalent** if the interval lies within [ − *δ*_*m*_, + *δ*_*m*_]. Margins *δ*_*m*_ are predefined through clinical risk assessment.

Gold-standard acceptance is evaluated jointly with absolute safety thresholds. Failure of either triggers escalation per the CDM hierarchy.

### AEGIS overview

**Fig. 5:**
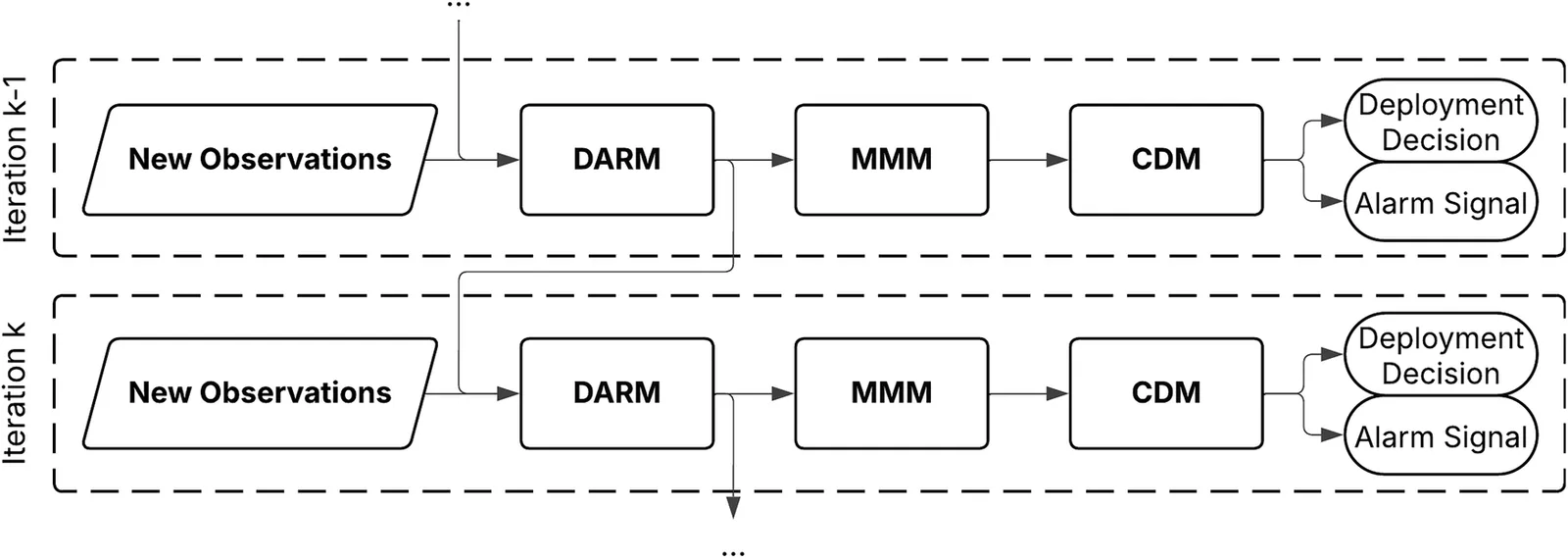
Integration of AEGIS core governance modules. The system architecture illustrates the continuous learning lifecycle across successive iterations (e.g., iteration *k*−1 and iteration *k*). In each cycle, new observational data is ingested by the DARM. The updated data and model are subsequently evaluated by the MMM. Finally, the CDM processes the monitoring metrics to independently output a deployment decision for the new candidate model and a parallel PMS ALARM signal for the currently released model.

The AEGIS infrastructure integrates the DARM, MMM, and CDM for transparent, automated, auditable model lifecycle management. Figure 5 illustrates the complete AEGIS infrastructure.

AEGIS bridges both regulatory paradigms from Table 7. The fixed performance reference comparison mechanism is designed to support FDA equivalence documentation. The MMM’s continuous monitoring is structured to align with MDR surveillance mandates. The four-category deployment taxonomy maps to both: APPROVE aligns with PCCP scope and EU Article 43(4) exemptions; REJECT triggers escalation under each jurisdiction. The parallel ALARM signal addresses PMS obligations independently of the deployment decision.

### AEGIS instantiation

AEGIS is domain-agnostic: the core modules define *what* to monitor and govern, while *instantiation parameters* define *how* they operate in specific contexts. Configuration includes: data modality, model architecture, primary metrics, MLcps weighting, drift detection method, clinical thresholds, and safety action mappings. Table 11 summarizes configuration for two proof-of-concept instantiations, and the pattern is straightforward: the same modules and the same priority-ordered hierarchy, with domain-configured decision logic. Only the module structure and the priority-ordering mechanism are invariant across domains; the imaging instantiation redefines APPROVE/REJECT via ${P}_{G}^{{\rm{rel}}}$ and deactivates two intermediate categories.

**Table 11:** AEGIS instantiation parameters

Parameter	AEGIS Definition	Sepsis Instantiation	BraTS Instantiation
Data modality	Any supervised learning data	Tabular EHR time-series (202 features)	3D multi-parametric MRI
Model architecture	Agnostic (wrapper)	RF	nnU-Net ResENC-M
Primary metrics	Domain-configurable	Sens., Spec., ROC-AUC	DSC (TC)
MLcps weighting	User-defined via risk assessment	Sens.: 1.5, AUC: 1.3, Bal. Acc.: 1.1, Spec.: 1.0 (raw; normalized internally)	DSC: 1
Drift detection	Pluggable method	Bonferroni-corrected KS drift score	Performance comparison
Clinical thresholds	Risk-assessment derived (ISO 14971:2019)	Sens. <0.65^*^	DSC < 0.676 (TC)^**^
Safety action	ALARM/REJECT triggers	Same CDM logic	Same CDM logic

*Sens. sensitivity, Spec. specificity, Bal. Acc. balanced accuracy, EHR* electronic health record.

^*^deployment safety performance floor (REJECT triggers). See Table 10 for complete hierarchy.

^**^the deployment safety performance floor (*P*^fail^ = 0.676) is derived from the performance of the model trained on the full cohort on the Golden set, defined as the fixed performance reference (*P*^ref^ = 0.726) minus a tolerance *τ*^safe^ = 0.05 (see Table 4).

All thresholds are illustrative; production values require ISO 14971:2019 hazard analysis with documented traceability and/or alignment with published clinical performance benchmarks (see Supplementary S2.3).

Configuration also includes specifying which decision categories are active for a given deployment context. Not all four deployment decision categories need to be enabled in every instantiation; which categories are appropriate depends on the clinical risk profile and regulatory pathway. For a high-risk autonomous system, CONDITIONAL APPROVAL may be clinically inappropriate, i.e., there is no safe middle ground of ‘deploy with enhanced monitoring’ when the cost of an error is severe, so the instantiation would use only REJECT, CLINICAL REVIEW, and APPROVE. For a low-risk screening tool with a large operator monitoring infrastructure, CLINICAL REVIEW might be collapsed into CONDITIONAL APPROVAL. Conversely, a system subject to strict regulatory oversight might require that any deviation from APPROVE triggers CLINICAL REVIEW rather than CONDITIONAL APPROVAL. The active categories and their trigger thresholds must be documented in the PCCP and derived through published clinical performance benchmarks and formal risk assessment per ISO 14971:2019. This configurability is a feature, not a limitation: AEGIS provides the category definitions and priority logic; the instantiation selects the subset appropriate to the clinical context.

**Fig. 6:**
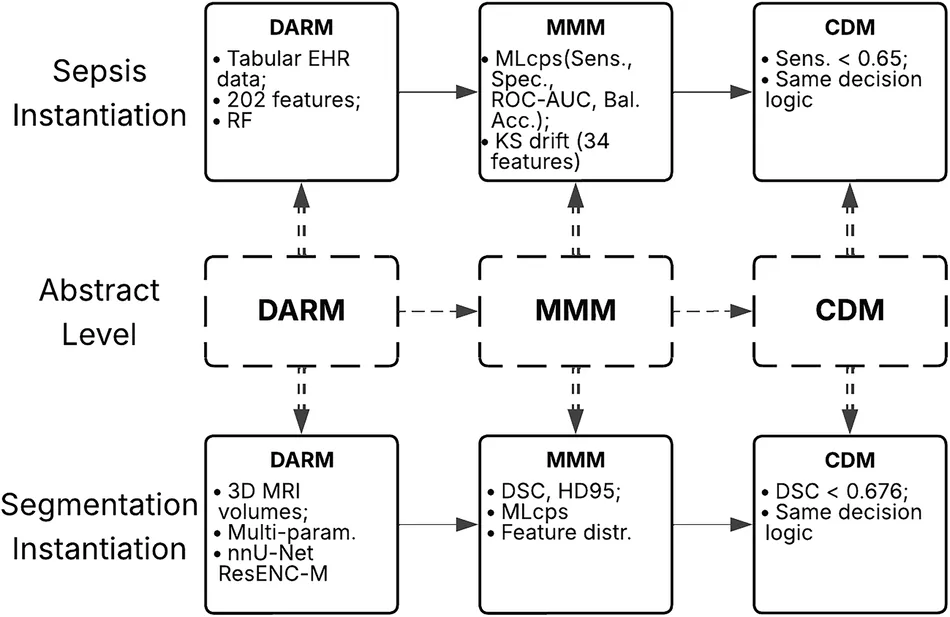
AEGIS instantiation pattern. The schematic illustrates the modular AEGIS architecture, which features domain-agnostic modules at the abstract level (middle). Each concrete instantiation—shown for sepsis prediction (top) and brain tumor segmentation (bottom)—configures the infrastructure with domain-specific data, models, metrics, and thresholds. While the specific parameters are calibrated to each clinical domain, both instantiations are built from the same modules and the same priority-ordered hierarchy; the decision logic itself is domain-configured (the imaging instantiation redefines APPROVE/REJECT via ${P}_{G}^{{\rm{rel}}}$ and deactivates two intermediate categories).

Figure 6 illustrates this relationship.

#### Proof-of-concept validation

We instantiated AEGIS for two distinct applications to demonstrate generality. *This validation is a proof-of-concept demonstration, not clinical evaluation*. The models serve as vehicles to exercise governance architecture; they have not undergone clinical validation.

The two domains were chosen for complementary characteristics: sepsis prediction (structured time-series with established thresholds) and image segmentation (high-dimensional spatial data with different metrics). *The goal is demonstrating AEGIS generalizability, not achieving SOTA performance*. Models use conventional architectures without extensive optimization.

Validation encompasses:

- Iterative development simulating continuous learning scenarios
- PCCP enforcement through automatic safety checks
- PMS and drift detection

#### Sepsis prediction instantiation

The first instantiation addresses early sepsis prediction using PhysioNet/Computing in Cardiology Challenge 2019 data^22^. We selected this dataset for accessibility: unlike MIMIC-III/IV (requiring credentialing and data use agreements), PhysioNet data are openly available, supporting reproducibility.

#### Data source and preprocessing

Raw data comprised longitudinal records of vital signs (heart rate, pulse oximetry, temperature, blood pressures, respiration rate, end-tidal CO_2_) and laboratory values (base excess, bicarbonate, pH, BUN, lactate, leukocytes, fibrinogen, platelets). Feature engineering aggregated time-series into fixed-length vectors using statistical features (mean, standard deviation, minimum, maximum, last value, slope) over each ICU stay. Demographics and length of stay were included, yielding 202 features. Missing values were median-imputed. Features were not standardized, as the RF classifier is invariant to monotonic feature transforms.

#### Prediction time horizon

Features are computed over each patient’s complete ICU stay; labels indicate whether sepsis occurred during that stay. This retrospective formulation serves the governance demonstration. Clinical deployment requires specifying:

- prediction time relative to admission,
- available measurement window, and
- label definition relative to prediction time.

*Model Architecture:* We employed a single RF classifier^23^ (300 estimators, max depth 12, min samples split 10, min samples leaf 5, balanced class weighting, Out-Of-Bag (OOB) scoring enabled). These hyperparameters were selected for inter-iteration stability rather than maximum predictive power: the governance demonstration requires that metric changes reflect data perturbations, not model instability. Threshold optimization used OOB predictions targeting 75% sensitivity, reflecting clinical priority of detecting true sepsis cases. OOB predictions provide honest, cross-validated probability estimates without requiring a separate validation fold. Features were median-imputed but not standardized, as RFs are invariant to monotonic feature transforms. The model was chosen as a conventional baseline; AEGIS is architecture-agnostic.

*DARM Implementation:* The DARM enforced patient-safe data separation, ensuring all samples from a unique patient ID remained in a single split. The dataset comprised 20,336 patients from Hospital A of the PhysioNet 2019 Challenge. The Golden dataset (20% of patients: 4067 samples) was reserved for unbiased evaluation. The remaining 80% formed the dynamic pool: half (8134 samples) for the initial model, half for iterative batches. Data accumulation (${{\mathcal{D}}}_{T,k+1}={{\mathcal{D}}}_{T,k}\cup {{\mathcal{D}}}_{D,k}$) is unconditional; it occurs at every iteration regardless of the CDM decision. The governance decision controls what happens to the model, not what happens to the data.

*MMM Implementation:* The MMM tracked three metric categories: model performance (accuracy, ROC-AUC, Area Under the PR Curve (PR-AUC), F1, sensitivity, specificity, PPV, NPV, False Negative Rate (FNR), False Positive Rate (FPR)), fairness (bias score comparing demographic groups), and distribution (drift score).

For unified assessment, we adopted the MLcps metric^12^, which projects multiple metrics onto polar coordinates and calculates enclosed area. We configured raw weights: Sensitivity 1.5, ROC-AUC 1.3, Balanced accuracy 1.1, Specificity 1.0. The official MLcps package normalizes these internally to proportional angular sectors. These weights are illustrative; production implementations should derive weights through stakeholder consensus with ISO 14971:2019 documentation.

#### Drift detection specification

Feature-level drift is assessed via two-sample KS tests across monitored features, with Bonferroni correction applied to control the family-wise error rate.

The aggregate drift score ${{\mathcal{S}}}_{D}$ represents the proportion of features exhibiting statistically significant distributional shift. Minor drift is flagged when ${{\mathcal{S}}}_{D}$ falls in [0.30, 0.70]; major drift when it exceeds 0.90. The intermediate band [0.70, 0.90] is an intentional buffer between the minor and major regimes: it avoids oscillation between CONDITIONAL APPROVAL and ALARM near the boundary and reserves the ALARM response for clearly pervasive shift. The cut points were derived from the stationary-baseline variability observed in iterations 0–5 rather than chosen arbitrarily.

The drift score quantifies covariate drift, i.e., changes in the marginal distribution of input features. Concept drift (shifts in the conditional distribution, i.e., changes in the feature-outcome relationship) is partially addressed through a *proxy* check at Priority 2 (see Table 10), which identifies cases where feature distributions appear stable but the released model degrades on new data, a pattern consistent with concept drift, though not conclusive evidence of it without outcome label monitoring. Direct concept drift detection, including explicit modeling of the feature-outcome relationship over time, is identified as a future extension (Section “Discussion”).

Clinical threshold estimates are informed by performance bounds of established standard-of-care screening tools; for instance, setting a minimum sensitivity floor of 0.65 anchors the ML model to the pooled sensitivity of standard Early Warning Scores reported in recent clinical systematic reviews^24^, ensuring the automated system does not regress below existing clinical baselines. Additional thresholds include specificity of 0.55, ROC-AUC of 0.70, MLcps of 0.65, NPV of 0.90, and a maximum FNR of 0.35. These values serve as demonstration anchors calibrated to published sepsis systematic reviews^24,25^; their purpose is to exercise the governance machinery and verify that the CDM routes correctly under each threshold configuration, not to propose universally valid operating values. All threshold values used in this proof-of-concept are illustrative; production implementations must derive each value through formal ISO 14971:2019 hazard analysis, with traceability to specific hazard identifiers documented in Supplementary Section S2.3.

The sensitivity floor of 0.65 corresponds to the aggregate sensitivity reported in refs. ^24,25^, and (for the imaging instantiation) the DSC floor of 0.676 is the fixed reference (0.726) minus a 5% non-inferiority margin. We also report a threshold-sensitivity analysis in which each key threshold is perturbed by  ± 0.05 and the resulting changes in iteration decisions are tabulated. Routing is stable except near the intended decision boundaries: across all perturbations only two iterations ever change, and only under large shifts—raising the sensitivity floor to 0.70 (or the buffer to 0.71) reclassifies iteration 8, and loosening the fixed-reference tolerance to ≥0.03 reclassifies iteration 7—while widening the minor-drift band over [0.25, 0.40] changes no decision (Supplementary Table S30).

To systematically characterize the stability and boundary sensitivities of the CDM for the imaging instantiation, we performed two post-hoc ablation studies across the 12-iteration Glioma segmentation lifecycle. The first, a Performance Floor Ablation, evaluates the structural impact of altering the safety margin *P*^fail^ used to establish the absolute performance floor baseline relative to the iteration-0 reference of *P*^ref^ = 0.726 (Supplementary Table S31). The performance floor ablation (Table S31) illustrates the framework’s parameter sensitivity and dual-criteria logic: while relaxing the absolute safety margin allows early models (iterations 1–4) to transition from REJECT to APPROVE, iterations 8 through 12 remain consistently rejected, confirming that the framework’s non-regression checks successfully enforce principled rejections for degrading models even when minimum absolute safety floors are satisfied.

The second, a Regression Tolerance Sweep, evaluates the downstream deployment impact of modifying the tolerance margin *τ*^reg^ allowed before a candidate model is rejected due to performance degradation compared to the most recently active, fully approved release version ${P}_{G}^{{\rm{rel}}}$ (Supplementary Table S32). The regression tolerance sweep (Table S32) demonstrates that increasing the non-regression tolerance margin effectively relaxes this strict condition; when the margin is sufficiently widened (*τ*^reg^ ≥ 0.019), the non-regression constraint is essentially removed, allowing iterations 8–12 to transition to APPROVE and preventing permanent-rejection locking caused by minor statistical variance (per-iteration static margins are given in Section “Brain tumor segmentation instantiation”; the reported boundary reflects the dynamic ${P}_{G}^{{\rm{rel}}}$ update rule, under which ${P}_{G}^{{\rm{rel}}}$ advances on each APPROVE so that the flip cascades through iterations 8–12 once iteration 8 is approved).

Explainability was assessed using SHAP analysis^26^, quantifying feature importance concentration in top features.

*CDM Implementation:* The CDM implemented the four deployment decision categories and parallel PMS ALARM signal, aligned with US and EU regulatory guidance (Table 12):

**Table 12:** Decision taxonomy with regulatory mapping^a^

Decision	Definition	FDA PCCP Status	EU MDR/AI Act	Trigger
APPROVE	All metrics satisfied	Within PCCP scope	Art. 43(4) exemption	All pass
CONDITIONAL APPROVAL	Enhanced monitoring	Limited PCCP scope	Enhanced PMS	Minor drift or low trust scores
CLINICAL REVIEW	Human validation required	May exceed PCCP	Pre-modification review	Near threshold
ALARM	Released model at risk in field; issued as a PMS signal independently of the new model deployment decision	Activates PMS reporting; may require vigilance report depending on severity	EU MDR Art. 83 PMS obligation; potential Art. 87 serious incident notification	PMS safety performance floor breach; released model regression; major distributional shift
REJECT	Deployment blocked	New 510(k) required	New CA	Safety violation

ALARM is a parallel PMS signal and is not part of the deployment decision hierarchy. It may be co-issued with any deployment decision. When ALARM is absent, no label is emitted. Regulatory reporting obligations triggered by ALARM depend on device classification, severity assessment, and jurisdictional requirements; manufacturers should document thresholds and escalation pathways in their PMS plan.

^a^Regulatory status mappings represent operational analogies intended to guide PCCP documentation structure; definitive regulatory classification depends on device-specific intended use, risk classification, and jurisdictional interpretation, and should be confirmed with regulatory counsel prior to any submission.

The decision hierarchy evaluates conditions in priority order (Table 10). This ensures safety violations are detected first.

**Two-tier threshold architecture**. The deployment safety performance floor defines floors below which deployment is prohibited (REJECT). Performance thresholds define acceptable bounds; falling below triggers CLINICAL REVIEW. This buffer ensures human oversight before rejection.

**CDM specification (deterministic implementation):**

To ensure complete auditability and reproducibility, the CDM is specified as a deterministic function:

**Inputs:** Metrics on ${{\mathcal{D}}}_{G}$ and ${{\mathcal{D}}}_{D,k}$ (sensitivity, specificity, ROC-AUC, NPV, MLcps), drift indicators (minor/major flags, drift score), trustworthy AI indicators (bias score, explainability score), and gold-standard acceptance results (threshold comparisons or CI-based test outcomes).

**Outputs:** {decision_label, trigger_reasons[], required_actions[], logged_artifacts[]}.

**Logging requirements:** For audit trail completeness, the CDM must record: metric values at decision time, threshold/margin values used, dataset identifiers and hashes, drift statistics with p-values, and explicit trigger reasons. This logging enables regulatory review of every governance decision.

**Priority logic:**

1. If the candidate model performance on Golden dataset at iteration *k* is less than the deployment safety performance floor (*P*_*G*,*k*_ < *P*^fail^ = 0.65)  → **REJECT** [P1] (reason: deployment safety performance floor violation)
2. Else if any metric in buffer zone (sensitivity < 0.66 OR specificity  < 0.60) OR the regression of the candidate model performance on Golden dataset at iteration *k* from the performance of the first fully approved model on the Golden dataset is sufficiently large (*τ*^buf^ < *P*^ref^ − *P*_*G*,*k*_)  → **CLINICAL REVIEW** [P2] (reason: borderline safety/performance)
3. Else if minor drift ($0.30\le {{\mathcal{S}}}_{D} < 0.70$) OR TAI concern  → **CONDITIONAL APPROVAL** [P3] (reason: enhanced monitoring required)
4. Else  → **APPROVE** [P4] (reason: all criteria satisfied)

*PMS* ALARM* signal (evaluated in parallel using released model on*${{\mathcal{D}}}_{D,k}$; *fires if any condition is true):*

- A1) The most recently fully approved released model performance on Drifting set is less than the PMS safety performance floor (${P}_{D}^{{\rm{rel}}} < {P}^{{\rm{PMS}}}$)  → **ALARM** (reason: PMS safety performance floor breached)
- A2) The most recently fully approved released model performance on Drifting set is less than the most recently fully approved released model performance on Golden set by more than a tolerance (${P}_{D}^{{\rm{rel}}}\le {P}_{G}^{{\rm{rel}}}-{\tau }^{{\rm{PMS}}}$)  → **ALARM** (reason: released model regressed from the reference on new data)
- A3) If the Bonferroni-corrected KS drift score is sufficiently large ($0.90 < {{\mathcal{S}}}_{D}$) → **ALARM** (reason: major distributional shift threatens released model validity)

The composite output is reported as (deployment decision) or (deployment decision + ALARM) when any of A1–A3 fires. Absence of ALARM requires no label.

Complete algorithmic pseudo-code and implementation guidance appear in Supplementary Section S3.3.

**Implementation note on acceptance criteria**. This proof-of-concept employs deterministic threshold comparisons (e.g., sensitivity ≥ 0.65) rather than CI-based equivalence testing. This choice reflects the pedagogical goal of demonstrating core functionality with maximum interpretability, appropriate given the large evaluation dataset (*N* = 4067 patients). For production deployments with smaller post-market samples, CI-based non-inferiority testing is recommended.

**Fixed performance reference comparison coupling**. The fixed performance reference comparison mechanism serves two distinct functions in the composite CDM output. First, it acts as a trigger for the deployment decision at P2 (CLINICAL REVIEW): if the candidate model performance on Golden dataset at iteration *k* (*P*_*G*,*k*_) has regressed from the performance of the first fully approved model on the Golden dataset (*P*^ref^) by more than a tolerance (*τ*^buf^), a CLINICAL REVIEW is triggered even when absolute metrics remain above the deployment safety performance floor. Second, it provides the reference (i.e., ${P}_{G}^{{\rm{rel}}}$, which is the most recently fully approved released model performance on Golden set) against which the PMS ALARM trigger A2 is evaluated, i.e., if the most recently fully approved released model performance on Drifting set (${P}_{D}^{{\rm{rel}}}$) has degraded by more than a tolerance (*τ*^PMS^) from its reference (${P}_{G}^{{\rm{rel}}}$), ALARM fires. Upon initial APPROVE, AEGIS stores validated metrics both as *P*^ref^ and as ${P}_{G}^{{\rm{rel}}}$ for reference checks. Subsequent APPROVE fires update ${P}_{G}^{{\rm{rel}}}$ to the newly released model’s performance on the Golden set.

*P*^ref^ *vs* ${P}_{G}^{{\rm{rel}}}$**distinction**. The performance of the first fully approved model on the Golden dataset (*P*^ref^) is fixed at the first APPROVE and never updated, while the most recently fully approved released model performance on Golden set (${P}_{G}^{{\rm{rel}}}$) is a rolling reference that advances with each subsequent APPROVE. The AEGIS’s dual-criterion approach ensures that the candidate model performance on Golden dataset at iteration *k* (*P*_*G*,*k*_) does not silently degrade below the released performance reference (${P}_{G}^{{\rm{rel}}}$) or the fixed performance reference (*P*^ref^).

**Minor drift configuration**. The minor drift range (0.30–0.70) in Table 10 represents a configurable threshold for triggering enhanced monitoring via CONDITIONAL APPROVAL. In this validation, all four deployment decision categories are enabled and exercised. The minor drift range was widened from [0.35, 0.70] to [0.30, 0.70] based on threshold calibration analysis of stationary baseline variability (iterations 0–5), consistent with ISO 14971:2019 principles of deriving thresholds from observed operational characteristics. To avoid conflating calibration data with evaluation data, we note that the stationary iterations 0–5 are used for calibration only and have a drift score of exactly 0.000; the widening therefore does not change any of their deployment decisions. We report the iteration decisions under both the original [0.35, 0.70] and the revised [0.30, 0.70] bands (and the wider [0.25, 0.70]/[0.40, 0.70] settings) so the effect of the calibration step is fully transparent: no iteration’s deployment decision changes under any of these bands, confirming that the widening does not flatter the reported results (Supplementary Table S30). The complete mapping of abstract AEGIS parameters to specific values and clinical justifications for the sepsis prediction instantiation is summarized in Table 13.

**Table 13:** Mapping of abstract AEGIS parameters to sepsis prediction parameters

AEGIS Parameter	Concrete Value	Clinical Justification	CDM Decision
*P*^ref^	Sens.: 0.723, Spec.: 0.933, AUC: 0.922	performance of the first fully approved model on the Golden dataset	fixed performance reference
*P*^fail^	Sens.: 0.65	derived from published systematic reviews of sepsis prediction performance^24,25^	REJECT
*R*_*G*_	0.66≤ Sens. 0.60≤ Spec.	Performance thresholds (buffer zone)	CLINICAL REVIEW
Major drift threshold	$0.90 \, < \, {{\mathcal{S}}}_{D}$	> 90% of 34 key features shifted (KS test)	ALARM (A3)
Minor drift range	$0.30\le {{\mathcal{S}}}_{D}\le 0.70$	Moderate distributional change detected	CONDITIONAL APPROVAL
${{P}^{\mathrm{PMS}}}^{{\rm{a}}}$	Sens.: 0.65	= *P*^fail^	ALARM (A1)
*τ*^PMS^	0.020–0.030 sens. points	Detects gradual decline before PMS safety performance floor is breached	ALARM (A2)
*P*_*G*,*k*_, *P*_*D*,*k*_	MLcps (Sens.: 1.5, AUC: 1.3, Bal. Acc.: 1.1, Spec.: 1.0; raw weights, normalized internally)	Sensitivity-weighted per clinical priority	Composite tracking
NPV floor	≥0.90	Safe negative triage in screening context	Safety constraint
FNR ceiling	≤0.35	Maximum tolerable missed sepsis cases	Safety constraint
Model architecture	Single RF (300 trees, depth 12, OOB threshold)	—	Architecture specification
*τ*^buf^	0.015 (sensitivity)	Detects subtle regression (>*τ*^buf^ = 0.015, i.e., 0.015 in absolute sensitivity units, decline from fixed performance reference)	CLINICAL REVIEW
$| {{\mathcal{D}}}_{G}|$	*N* = 4,067 (20% of 20,336)	Sufficient for deterministic thresholds	Evaluation dataset

This table provides the sepsis-specific instantiation of the infrastructure-level CDM priority hierarchy defined in Table 10.

*Sens. sensitivity, Spec. specificity, Bal. Acc.* balanced accuracy.

Notations: *P*^ref^ = fixed performance reference; *τ*^buf^ = threshold defining the performance buffer zone; *τ*^PMS^= regression tolerance for the already released model; *P*^fail^/*P*^PMS^ = deployment/PMS safety performance floor below which REJECT/ALARM is triggered; *R*_*G*_ = acceptable performance ranges for Golden dataset; *P*_*G*,*k*_/*P*_*D*,*k*_ = candidate model performance on Golden/Drifting dataset at iteration $k;{{\mathcal{S}}}_{D}$ = Bonferroni-corrected KS drift score; $| {{\mathcal{D}}}_{G}|$ = ﻿Golden set size.

^a^In this proof-of-concept, *P*^PMS^ is set equal to *P*^fail^ for simplicity. Production implementations may set *P*^PMS^ > *P*^fail^ to provide an early field warning before the deployment rejection threshold is reached; the two thresholds serve different governance functions and are evaluated on different models against different datasets.

Note: *All thresholds are illustrative; production values require ISO 14971:2019 hazard analysis with documented traceability and/or alignment with published clinical performance benchmarks (see Supplementary S2.3)*.

*Iterative Simulation:* The simulation ran 11 iteration cycles, systematically exercising the governance decision space. The model at iteration 0 trained on 8134 samples (714 sepsis cases, 8.8%). Iterations 1–5 used real data batches (~1626 samples each) from the iterative pool. Iterations 6–10 used mixed and synthetic batches designed to exercise each of the four deployment decision categories and both ALARM composite states:

- **Cross-site minor drift** (iteration 6): 50% Hospital A + 50% Hospital B patients (cross-hospital distributional difference); targets CONDITIONAL APPROVAL via minor drift detection
- **Regression from fixed performance reference***P*^ref^ (iteration 7): Clean Hospital A batch (*n*=3000). Cross-site contamination from iteration 6 causes subtle sensitivity regression detected by fixed performance reference comparison (>*τ*^buf^ = 0.015 in absolute sensitivity units, decline from fixed performance reference); targets CLINICAL REVIEW
- **Extreme distributional shift** (iteration 8): 5 × scaling + 5 × *σ* offset on all 34 drift features (*n*=100, small batch to avoid model corruption); targets APPROVE + ALARM (new model unaffected by small batch; PMS ALARM fires via A3 major drift)
- **Recovery** (iteration 9): Clean Hospital A resample (*n*=3000); demonstrates system recovery after drift/regression; targets APPROVE
- **Catastrophic corruption** (iteration 10): 80% positive label flip, 30% negative label flip, *σ* = 4 × std Gaussian noise on all features (*n*=25,000); targets REJECT + ALARM (new model sensitivity collapses below 0.65; released model simultaneously fails on same corrupt batch)

The specific perturbation magnitudes are illustrative rather than calibrated to real-world drift characteristics. While synthetic scenarios enable controlled evaluation, they may not fully capture real-world complexity from demographic changes, equipment variations, or clinical practice evolution^27^. Future validation should incorporate documented real-world drift events.

#### Brain tumor segmentation instantiation

The second instantiation demonstrates the AEGIS’s utility in the domain of medical imaging, specifically targeting the automated segmentation of brain tumors in MRI scans. Radiology remains a cornerstone for clinical AI applications, representing one of the most established fields for ML integration in healthcare. To ensure a robust evaluation, we utilized data and task definitions from the Medical Image Computing and Computer Assisted Intervention (MICCAI) BraTS challenge^28,29^. This benchmark was selected due to its long-standing maturity and the availability of extensively validated methods, providing a standardized environment to test AEGIS’s ability to govern high-dimensional spatial data.

*Dataset and the studied task:* This study utilizes data from the BraTS 2023 and 2024 challenges, focusing on the pre-operative and post-treatment GLI cohorts. The dataset comprises multi-parametric MRI (mpMRI) scans, including Native T1-weighted (T1n), Contrast-enhanced T1-weighted (T1c), T2-Weighted (T2w), and T2-weighted Fluid-attenuated inversion recovery (T2f) sequences. These scans are paired with expert-validated segmentation masks that delineate distinct tumor subregions. In pre-operative cases, these masks identify the Enhancing Tumor (ET), NETC, and peritumoral Edema (ED). For post-treatment scans, the segmentation task is extended to include the Resection Cavity (RC).

**Fig. 7:**
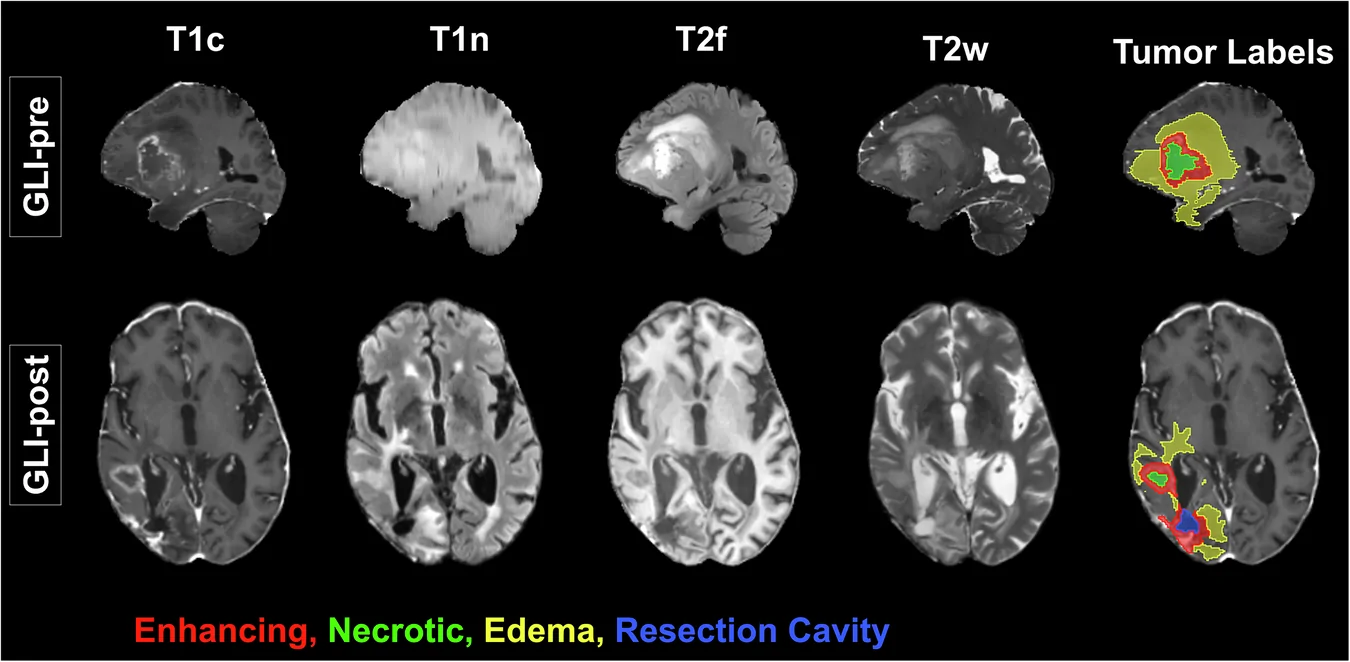
GLI tumors in pre-operative and post-treatment mpMRI. Each row shows one representative case: the top row is a pre-operative case (GLI-pre) and the bottom row a post-treatment case (GLI-post). The first four columns show the mpMRI sequences in greyscale: T1-weighted contrast-enhanced (T1c), T1-weighted native (T1n), T2-weighted fluid-attenuated inversion recovery (T2f), and T2-weighted (T2w); the fifth column overlays the reference segmentation on the anatomical image. Segmentation colors: red, enhancing tumor; green, necrotic core; yellow, peritumoral edema; blue, resection cavity.

Figure 7 illustrates the complexity of this task by visualizing the heterogeneous appearance of these tumor subregions across both pre-operative and post-treatment mpMRI modalities.

*Tumor segmentation workflow.* To operationalize AEGIS for volumetric data, we utilized the nnU-Net ResENC-M architecture^30^, version 2 (nnU-Net v2)—a highly robust solution that has consistently secured winning positions in several BraTS challenges. The model was implemented using its default configurations to ensure a standardized and reproducible environment for evaluating the AEGIS governance modules. The network accepted the four mpMRI sequences as multi-channel inputs and underwent training for 1500 epochs for direct label-wise segmentations. While various quantitative metrics are common to evaluate segmentation performance, we chose to use the DSC due to its prevalence and conventional use in the medical image segmentation community.

To demonstrate the modular flexibility of the MMM, the underlying architecture is engineered to dynamically support a variety of distinct segmentation metrics across heterogeneous target tumor subregions. For presentation simplicity and focus in the main text, Table 3 and Table 4 restrict their evaluation to the composite TC using the volumetric DSC. However, the AEGIS infrastructure natively accommodates plug-and-play reconfiguration for alternative tumoral regions such as the WT and NETC, as well as alternative surface-overlap criteria including the NSD. To illustrate how changing these region-metric configurations shifts downstream change-control decisions, a cross-modality parameter tracking analysis is detailed in Supplementary Tables S34 and S35.

Although the model was trained to segment all constituent subregions, AEGIS MMM focused its governance logic specifically on the TC, which is the composite region of the ET and NETC. This subregion was prioritized for two primary reasons. First, the TC holds important clinical significance, as it is the primary target for radiotherapy and surgical planning; therefore, its accuracy is the most critical metric for safety-centric governance. Second, focusing on a consolidated clinical target streamlines the statistical analysis for drift detection and the subsequent decision-making process, including automated acceptance or rejection, ensuring AEGIS remains computationally efficient and clinically interpretable.

*Iterative simulations.* The evaluation began by establishing a fixed performance reference using a held-out *Golden dataset* (${{\mathcal{D}}}_{G}$) consisting of 200 subjects from the BraTS post-treatment GLI cohort. The corresponding model was trained on the remaining 1150 subjects, and its performance on the ${{\mathcal{D}}}_{G}$ was recorded as the fixed performance reference.

To simulate a continuous learning lifecycle, we implemented an iterative training scheme. The process was initialized by training a model on a small subset of 200 subjects from the post-treatment training pool. In each subsequent iteration, a new Drifting Dataset was introduced to the MMM to assess real-time stability. While metrics from the drifting data did not dictate the final release status, significant deviations from the Golden Dataset performance served as early warnings for potential catastrophic forgetting or distribution shifts. Following each evaluation, the new data was assimilated into the training pool for the next iteration, reflecting a continuous data-acquisition and retraining pipeline.

This iterative process continued until the entire post-treatment cohort was integrated. To evaluate the AEGIS’s resilience against significant distribution shifts, we then introduced 1251 pre-operative datasets. We hypothesized that the model, originally trained to segment the RC, would maintain its robustness despite the absence of this feature in pre-operative scans. The full experimental sequence comprised 12 iterations, as summarized in Table 3.

To operationalize the CDM, we established a deterministic performance threshold based on the model trained on the full cohort. Following regulatory logic for non-inferiority, the threshold for model approval was set at a 5% margin below the fixed performance reference (*P*^ref^ = 0.726), yielding *P*^fail^ = 0.726 − 0.05 = 0.676. A model iteration is only eligible for deployment if its performance on the static Golden Dataset remains above this predefined deployment safety performance floor. Parallel to this, the Drifting Dataset was monitored to detect shifts in the distribution of incoming data batches. A decrease in DSC on the drifting set relative to the previous iteration serves as a proxy for identifying “hard” samples, such as rare tumor morphologies, noisy acquisitions, or expert labeling variability. This dual-track monitoring, static (Golden) and dynamic (Drifting), ensures that the model maintains core safety while the system remains sensitive to environmental changes.

The resulting regulatory decision logic for each iteration (*k*) is summarized as follows:

- **Reject**: if *P*_*G*,*k*_ < *P*^fail^ = 0.676 (Safety violation/Performance failure); OR if ${P}_{G,k} < {P}_{G}^{{\rm{rel}}}$ (Performance degradation below the last *released* version, rather than merely the immediately preceding iteration)
- **Approve**: if ${P}_{G}^{{\rm{rel}}} < {P}_{G,k}$ AND *P*_*D*,*k*−1_ < *P*_*D*,*k*_ (Performance improvement over released performance reference${P}_{G}^{{\rm{rel}}}$ with data stability).
- **Approve** + **Alarm**: if ${P}_{G}^{{\rm{rel}}} < {P}_{G,k}$ AND *P*_*D*,*k*_ < *P*_*D*,*k*−1_ (Golden set exceeds the released performance reference, triggering approval, but a potential distribution shift was detected in the new data, triggering an alarm).

To rigorously verify structural consistency in the underlying decision logic across heterogeneous medical domains, we conducted a Framework Compatibility Simulation by running the complete four-level deployment taxonomy directly on the volumetric Glioma dataset (Supplementary Table S33). In the primary neuro-oncology protocol, the intermediate deployment states of CLINICAL REVIEW and CONDITIONAL APPROVAL were intentionally deactivated due to the high-stakes nature of surgical and radiotherapy planning, where intermediate or ambiguous deployment configurations carry severe clinical risk and strict binary APPROVE or REJECT gates are the appropriate risk-calibrated choice. Forcing the execution of the four-tier taxonomy over the imaging data confirms that the AEGIS technical skeleton remains fully invariant: the infrastructure accommodates multi-tiered classification logic across 3D medical images, and manufacturers can seamlessly suppress active category layers to mirror a device’s specific clinical risk profile without altering the architectural integrity of the system. The comprehensive mapping of the abstract AEGIS parameters to the specific configurations used for the brain tumor segmentation instantiation is detailed in Table 4.

#### Note on active category configuration

The CONDITIONAL APPROVAL and CLINICAL REVIEW categories were intentionally not activated in this brain tumor instantiation. This reflects a deliberate configuration choice for the specific clinical context: brain tumor segmentation for treatment planning is a high-consequence application in which intermediate deployment states carry limited clinical utility, i.e., a model either meets the non-inferiority threshold for treatment planning use or it does not. Activating only REJECT, APPROVE, and ALARM for this instantiation is therefore appropriate, not a validation limitation. This asymmetry between instantiations illustrates the configurability principle described in Section “AEGIS instantiation”: the sepsis instantiation activates all four deployment decision categories to demonstrate the complete decision space, while the brain tumor instantiation activates a clinically appropriate subset. Taken together, the two instantiations establish both that all categories are reachable through configuration and that selective activation is a legitimate design choice. Production implementations of either domain should document the active category set and its clinical justification in the PCCP.

#### Note on strict non-regression condition

Similar to the deactivation of intermediate deployment states, the non-regression condition for the imaging instantiation was deliberately configured with zero tolerance (${P}_{G}^{{\rm{rel}}} < {P}_{G,k}$). This strict condition reflects the high-stakes nature of surgical and radiotherapy planning, where any spatial degradation carries severe clinical risk and buffer zones are clinically inappropriate. Notably, while a zero-tolerance rule theoretically risks permanent-rejection locking due to minor statistical variance, the supplementary regression tolerance sweep (Table S32) demonstrates the robust stability of these rejection decisions. Although sufficiently widening the tolerance margin (*τ*^reg^≥0.019) eventually allows the degrading models to pass, the REJECT decisions for iterations 8–12 remain completely stable across all tighter tolerances up to *τ*^reg^ = 0.018. Because the performance deficit substantially exceeded standard margins, meaning these iterations would still have been rejected even under the *τ*^reg^ = 0.015 configuration used in the sepsis instantiation, this confirms that the framework successfully enforced a principled rejection for genuine model degradation, rather than locking due to a rigid zero-tolerance parameterization.

#### Per-iteration flip-point clarification

Because iterations 8–12 sit at different absolute distances below ${P}_{G}^{{\rm{rel}}}=0.742$ (iteration 7’s Golden-set DSC), a naive *static* reading of the tolerance sweep (holding ${P}_{G}^{{\rm{rel}}}$ fixed at 0.742) would predict that only iteration 8 flips at *τ*^reg^ = 0.019, with iterations 9–12 individually requiring *τ*^reg^≥0.028, 0.036, 0.035 and 0.044 respectively (Table 14). The deployed CDM rule, however, *updates*${P}_{G}^{{\rm{rel}}}$ on each APPROVE (the “${P}_{G}^{{\rm{rel}}}$ update rule” in Section “AEGIS architecture”): once iteration 8 satisfies the tolerant check at *τ*^reg^ = 0.019 and is approved, ${P}_{G}^{{\rm{rel}}}$ rolls forward to 0.723, so iteration 9 (*P*_*G*,9_ = 0.714) is only 0.009 below the new reference (${P}_{G}^{{\rm{rel}}}=0.723$) and also passes at the *same* tolerance (*τ*^reg^ = 0.019); the update then chains through iterations 10–12, each clearing its own falling reference with non-negative slack. Table S32 therefore correctly shows all of iterations 8–12 transitioning to APPROVE at *τ*^reg^≥0.019 (the dynamic, rolling-reference behavior). Table 14 sets the two readings side by side so that both are verifiable per iteration: the static margins in isolation, and the rolling reference and slack that produce the cascade at a single tolerance.

**Table 14:** Per-iteration flip points for the brain-tumor regression tolerance sweep, under both readings at *τ* ^reg^ = 0.019

*τ* ^reg^ = 0.019						
		Static reading (${P}_{G}^{{\rm{rel}}}={P}_{G,7}=0.742$)		Dynamic reading		
Iter. *k*	*P*_*G*,*k*_	${P}_{G,k}-({P}_{G}^{{\rm{rel}}}-{\tau }^{\,{\rm{reg}}})$	Deploy. Decision	${P}_{G}^{{\rm{rel}}}$	${P}_{G,k}-({P}_{G}^{{\rm{rel}}}-{\tau }^{\,{\rm{reg}}})$	Deploy. Decision
8	0.723	+ 0.000	APPR.	0.742	+ 0.000	APPR.
9	0.714	− 0.009	REJ.	0.723	+ 0.010	APPR.
10	0.706	− 0.017	REJ.	0.714	+ 0.011	APPR.
11	0.707	− 0.016	REJ.	0.706	+ 0.020	APPR.
12	0.698	− 0.025	REJ.	0.707	+ 0.010	APPR.

The tolerant non-regression check is ${P}_{G,k}\ge {P}_{G}^{{\rm{rel}}}-{\tau }^{\,{\rm{reg}}}$, so the smallest *τ* ^reg^ at which iteration *k* would individually pass equals its margin below the reference. *Static reading* (columns 3–4): the reference is held fixed at ${P}_{G}^{{\rm{rel}}}=0.742$, iteration 7’s approved Golden-set DSC; only iteration 8 flips at *τ* ^reg^ = 0.019, and iterations 9–12 individually require progressively larger tolerances. *Dynamic reading* (columns 5–7): the deployed CDM advances ${P}_{G}^{{\rm{rel}}}$ to the newly approved value on each APPROVE, so at a single tolerance of *τ* ^reg^ = 0.019 every iteration clears its own (falling) reference, with non-negative slack throughout; iteration 8 passes exactly at equality. This is why Table S32 reports all of iterations 8–12 transitioning to APPROVE at *τ* ^reg^≥0.019.

### Robustness, sensitivity, and confidence analyses

To assess the governance machinery beyond the headline iteration outcomes, we report several analyses on the sepsis instantiation. All are derived from a single instrumented re-run of the iterative loop (identical seed, reproducing Table 1 exactly): the ablation and threshold sweep are pure post-hoc re-evaluations of the recorded per-iteration metrics and require no retraining, while the bootstrap and the fairness/explainability analyses reuse each iteration’s already-trained model for inference only. Complete per-iteration values are tabulated in the Supplementary Tables S27–S30.

*Bootstrap procedure*. For each iteration *k*, we draw *B* = 1000 *patient-level* resamples (with replacement) of the Golden evaluation set ${{\mathcal{D}}}_{G}$ and, where the decision depends on it, of the incoming Drifting batch ${{\mathcal{D}}}_{D,k}$. Because the engineered features are one row per patient, resampling rows is resampling patients, with no pseudo-replication. Within each replicate we recompute the performance metrics from the candidate model’s cached predictions (no model is retrained), recompute the Bonferroni-corrected KS drift score on the resampled batch, and re-evaluate the complete CDM decision. We report 95% percentile confidence intervals for the primary metrics, and define *decision confidence* as the fraction of replicates returning the same decision as the point estimate. A fixed random seed is used throughout for reproducibility.

*Decision confidence*. As defined above, decision confidence is the bootstrap probability that an iteration reproduces its point-estimate decision; it is reported for completeness and, in this deterministic proof-of-concept, is not used as a deployment gate.

*Component ablation*. We re-evaluate every iteration under four nested configurations, so that each element of the decision logic can be shown to change at least one decision a simpler rule would not produce: (A0) safety floor only; (A1) plus the performance buffer and the fixed-reference (gold-standard) comparison; (A1b) plus the minor-drift band; and (A2) the full system, additionally including the parallel PMS ALARM signal with its gold-standard deferral.

*Threshold-sensitivity sweep*. We perturb each governance threshold and tabulate the resulting changes in iteration decisions: ±0.05 around the sensitivity floor, the performance buffer, specificity, ROC-AUC, and the minor- and major-drift bounds, together with a finer grid {0,  0.005,  0.010,  0.015,  0.020,  0.030,  0.050} for the small fixed-reference tolerance.

A complementary, non-engineered evaluation of the drift detector on real cross-site shift (Hospital A vs. Hospital B) is reported with the sepsis results (Table 2).

#### Ethics

This study analyzed only publicly available, fully de-identified (anonymized) data from the PhysioNet/Computing in Cardiology Challenge 2019 (sepsis) and the Brain Tumor Segmentation (BraTS) Challenge (brain-tumor MRI). As secondary use of anonymized public data with no identifiable participants, the work did not constitute human-subjects research and required no additional ethics committee/institutional review board approval or new informed consent; the source datasets were collected under their providers’ own approvals and consent procedures. All analyses complied with the Declaration of Helsinki.

## Supplementary information

Supplementary Information

## Data Availability

All data used in this study are publicly available through the cited sources.

## Code availability

All code is available through the GitHub repository: https://github.com/ki-smile/aegis. The repository is public and includes a runnable reference implementation of the sepsis example—the Boolean decision engine, the DARM split logic, the MMM KPI calculations, and the CDM priority hierarchy—together with the software environment, key library versions, model hyperparameters, and the random seeds used to produce the reported results; runnable pseudocode is also provided in Supplementary Section S3.3. The implementation has been executed end-to-end on the PhysioNet data at the configuration reported here and reproduces the iteration decisions of Table 1. A Jupyter Notebook implementing the complete Glioma segmentation governance pipeline, together with a Docker image containing the checkpoints of all 12 model iterations and instructions for pulling and running the container, is now available in the public repository.
