# Supplementary material for: AEGIS: an operational infrastructure for post-market governance of adaptive medical AI under US and EU regulatory frameworks
Source: NPJ Digit Med. 2026 Oct 13;9:789. doi: 10.1038/s41746-026-03290-1 (PMC13662203; doi:10.1038/s41746-026-03290-1)
Supplement: Supplementary file 1 [file 41746_2026_3290_MOESM1_ESM.pdf]

Revised Supplement (Clean)

Manuscript: AEGIS: An Operational Infrastructure for Post-Market Governance of Adaptive Medical AI Under US and EU Regulatory Frameworks
Submission ID: 52387a85-c3a1-41ec-aeb3-be171877540e v3.0
Journal: npj Digital Medicine
Decision: Major Revision
Submission Round: 4
August 12, 2026

Supplementary Materials

AEGIS: An Operational Infrastructure for Post-Market Governance of Adaptive Medical AI Under US and EU Regulations

Fardin Afdideh[1], Mehdi Astaraki[1,2,3], Fernando Seoane[1,4,5,6], and Farhad Abtahi *† [1,4,7]

[1]Department of Clinical Science, Intervention and Technology, Karolinska Institutet, 17177 Stockholm, Sweden
[2]Department of Medical Radiation Physics, Stockholm University, Solna, Sweden
[3]Department of Oncology-Pathology, Karolinska Institutet, Solna, Sweden
[4]Department of Clinical Physiology, Karolinska University Hospital, 17176 Stockholm, Sweden
[5]Department of Textile Technology, University of Borås, Borås 50190, Sweden
[6]Department of Medical Technologies, Karolinska University Hospital, 141 57 Huddinge, Sweden
[7]Department of Biomedical Engineering and Health System, KTH Royal Institute of Technology, 14157 Huddinge, Sweden

*Corresponding author: Farhad Abtahi (farhad.abtahi@ki.se).

†E-mail addresses: Fardin Afdideh (fardin.afdideh@ki.se); Mehdi Astaraki (mehdi.astaraki@ki.se); Fernando Seoane (fernando.seoane@ki.se); Farhad Abtahi (farhad.abtahi@ki.se)

**Document Version:**	1.0
**Date:**	January 2026
**Status:**	For Journal Submission

Purpose: This supplement provides practical implementation resources for organizations responsible for developing and maintaining adaptive medical AI systems, including the clinical, risk management, regulatory, and quality professionals who operationalize the AI/ML Evaluation and Governance Infrastructure for Safety (AEGIS) infrastructure within those organizations. It accompanies the main manuscript describing the infrastructure architecture and validation.

Disclaimer: Completing this checklist and the accompanying templates supports good governance practice but does *not* guarantee regulatory acceptance, and is not a substitute for formal conformity assessment or regulatory review. Regulatory determinations depend on device-specific intended use, risk classification, and jurisdictional requirements and should be confirmed with regulatory counsel.

Contents

Document Overview 3

S1 AEGIS Implementation Checklist 5
- S1.1 Pre-Implementation Requirements . . . 5
- S1.2 Dataset Assimilation and Retraining Module (DARM) Configuration Checklist . 6
- S1.3 Model Monitoring Module (MMM) Configuration Checklist . . . 7
- S1.4 Conditional Decision Module (CDM) Configuration Checklist . . . 8
- S1.5 Regulatory Documentation Checklist . . . 9
- S1.6 Corrective and Preventive Action (CAPA) and Vigilance Integration Checklist . . 10

S2 AEGIS Configuration Template 12
- S2.1 Device/Application Identification . . . 12
- S2.2 Infrastructure Instantiation Parameters . . . 13
- S2.3 Threshold Specification Table . . . 13
- S2.4 ML Cumulative Performance Score (MLcps) Weight Configuration . . . 14
- S2.5 Gold Standard Baseline Documentation . . . 15
- S2.6 Decision Logic Specification . . . 16
- S2.7 Regulatory Pathway Mapping . . . 17
- S2.8 Risk Acceptability Matrix . . . 18

S3 AEGIS Implementation Guide 20
- S3.1 Infrastructure Adoption Workflow . . . 20
- S3.2 Deriving Thresholds from Risk Assessment . . . 20
  - S3.2.1 Step-by-Step Process . . . 20
  - S3.2.2 Worked Example: Sepsis Prediction . . . 21
- S3.3 Decision Logic Configuration . . . 21
  - S3.3.1 Decision Hierarchy . . . 21
  - S3.3.2 Constructing the Boolean Function . . . 21
- S3.4 Trustworthy AI (TAI) Integration . . . 23
  - S3.4.1 Explainability Configuration (SHapley Additive exPlanations (SHAP)) . . 23
  - S3.4.2 Fairness Metrics Configuration . . . 23
- S3.5 Regulatory Submission Guidance . . . 24
  - S3.5.1 Food and Drug Administration (FDA) Predetermined Change Control Plan (PCCP) Documentation Structure . . . 24
  - S3.5.2 European Union (EU) Technical Documentation Mapping . . . 24
- S3.6 Post-Deployment Operations . . . 25
  - S3.6.1 Monitoring Cadence Recommendations . . . 25
  - S3.6.2 Escalation Protocols by Decision Category . . . 25
  - S3.6.3 Audit Trail Requirements . . . 25
- S3.7 Post-Market Surveillance (PMS)/Periodic Safety Update Report (PSUR) Reporting Integration . . . 26
  - S3.7.1 AEGIS-to-PMS Report Mapping . . . 26
  - S3.7.2 PSUR Executive Summary Template . . . 26

S4 Robustness Analyses 27
- S4.1 Sepsis Prediction Instantiation . . . 27
- S4.2 Brain Tumor Segmentation Instantiation . . . 29

A Example Configuration — Sepsis Prediction **32**

B Example Configuration — Brain Tumor Segmentation **34**

C Decision Category Quick Reference **35**

D AEGIS CAPA Form Template **36**

Document Overview

This supplementary material is organized into three supplements:

- **S1: Implementation Checklist** — A verification tool ensuring all required infrastructure components are configured before deployment (Sections S1.1–S1.6)
- **S2: Configuration Template** — A fillable template for documenting domain-specific instantiation parameters (Sections S2.1–S2.8)
- **S3: Implementation Guide** — Step-by-step guidance for deploying the infrastructure (Sections S3.1–S3.7)
- **S4: Robustness Analyses** — Per-iteration quantitative data for both clinical domains. Section S4.1 (Sepsis Prediction Instantiation): decision confidence, component ablation, fairness subgroup gaps, and threshold-sensitivity sweeps. Section S4.2 (Brain Tumor Segmentation Instantiation): performance floor ablation, regression tolerance sweep, framework compatibility simulation, and MMM extensibility analyses.

Appendices:

- **A:** Example Configuration — Sepsis Prediction
- **B:** Example Configuration — Brain Tumor Segmentation
- **C:** Decision Category Quick Reference
- **D:** AEGIS CAPA Form Template

How to Use These Materials

1. **New Implementations:** Start with S3 (Guide) to understand the workflow, use S2 (Template) to document your configuration, and verify completeness with S1 (Checklist).
2. **Regulatory Submissions:** Use S2 (Template) as the basis for PCCP documentation; S1 (Checklist) provides an audit-ready verification record. Use S2.8 (Risk Acceptability Matrix) for ISO 14971 traceability.
3. **Existing Systems:** Use S1 (Checklist) to assess gaps; S3 (Guide) provides remediation guidance.
4. **Post-Market Operations:** Use S3.7 (PMS/PSUR Reporting) to structure surveillance reports; Appendix D (CAPA Form) for event documentation.

Acronyms

AEGIS	AI/ML Evaluation and Governance Infrastructure for Safety 1, 2, 3, 5, 9, 10, 11, 12, 13, 17, 18, 19, 20, 24, 26, 27, 30, 32, 34, 36, 37, 38, 40
AI	Artificial Intelligence 5, 9, 12, 17, 18
ALARA	As Low As Reasonably Achievable 19
BH	Benjamini-Hochberg 7
BraTS	Brain Tumor Segmentation 29
CA	Conformity Assessment 35
CAPA	Corrective and Preventive Action 1, 2, 3, 10, 26, 36, 37, 43

CDM	Conditional Decision Module 1, 5, 6, 7, 8, 9, 10, 13, 14, 15, 16, 17, 18, 20, 21, 22, 23, 24, 25, 26, 29, 34, 35, 36, 40, 42
CI	Confidence Interval 8, 9, 16, 21, 25
DARM	Dataset Assimilation and Retraining Module 1, 5, 6, 9, 13, 17, 24, 26, 40, 42
DSC	Dice Similarity Coefficient 31, 34
ECE	Expected Calibration Error 8
EHR	Electronic Health Record 32
EU	European Union 1, 4, 5, 9, 12, 17, 24, 35
FDA	Food and Drug Administration 1, 5, 9, 12, 13, 17, 23, 24, 35
FSCA	Field Safety Corrective Action 10
KPI	Key Performance Indicator 7, 8, 26
KS	Kolmogorov-Smirnov 7, 32
LIME	Local Interpretable Model-Agnostic Explanations 7
MDR	Medical Devices Regulation 4, 5, 9, 10, 11, 12, 13, 17, 24, 26, 35, 40
ML	Machine Learning 18
MLcps	ML Cumulative Performance Score 1, 7, 13, 14, 15, 16, 22, 26, 27, 32, 33, 34, 37
MMD	Maximum Mean Discrepancy 7
MMM	Model Monitoring Module 1, 3, 5, 7, 9, 13, 17, 18, 23, 24, 25, 26, 29, 40, 42
mpMRI	multi-parametric MRI 34
NB	Notified Body 5, 11, 12, 35
NETC	Non-Enhancing Tumor Core 31
NPV	Negative Predictive Value 26
NSD	Normalized Surface Distance 31
OOB	Out-Of-Bag 32
PCCP	Predetermined Change Control Plan 1, 3, 5, 9, 12, 13, 15, 17, 21, 24, 34, 35, 38, 40, 42
PMCF	Post-Market Clinical Follow-up 18, 24
PMS	Post-Market Surveillance 1, 3, 8, 9, 11, 17, 18, 19, 22, 24, 25, 26, 27, 28, 30, 31, 32, 33, 34, 35, 38, 39, 41, 42, 43
PPV	Positive Predictive Value 26
PR-AUC	Area Under the PR Curve 26
PRRC	Person Responsible for Regulatory Compliance 10, 37
PSI	Population Stability Index 7
PSUR	Periodic Safety Update Report 1, 3, 11, 20, 26, 27, 36
QMS	Quality Management System 5, 10, 12, 25, 36
RF	Random Forest 32
ROC-AUC	Area Under the ROC Curve 7, 8, 14, 15, 16, 26, 27, 28, 32, 33
SHAP	SHapley Additive exPlanations 1, 7, 23, 28
SOP	Standard Operating Procedure 10, 25, 26
TAI	Trustworthy AI 1, 7, 8, 9, 14, 17, 20, 21, 22, 23, 25, 33, 35, 41
WT	Whole Tumor 31

Acknowledgments

Template structures in Sections S1.6, S2.8, S3.7, and Appendix D are adapted from OpenRegulatory (openregulatory.com), which provides open-source templates for ISO 13485, ISO 14971, and EU Medical Devices Regulation (MDR) compliance.

S1 AEGIS Implementation Checklist

Successful deployment of AEGIS requires that each of its three modules—the DARM, the MMM, and the CDM—be configured correctly and that the surrounding organizational, regulatory, and quality-management prerequisites are in place before the first governance cycle begins. This checklist operationalizes the pre-deployment verification process: it translates the architectural requirements described in the main manuscript into a structured, itemized record that can be completed, signed, and retained as evidence of readiness. Organizations should treat completion of this checklist as a mandatory gate before activating AEGIS in any clinical setting, and should retain the signed document as part of the Quality Management System (QMS) file and, where applicable, as supporting evidence for a PCCP submission or EU MDR technical file.

S1.1 Pre-Implementation Requirements

Before any module-level configuration can begin, an organization must establish the regulatory, risk-management, and clinical foundations upon which AEGIS thresholds and decision logic will rest. This subsection verifies that the governing legal pathway (FDA PCCP, EU MDR classification, EU AI Act risk category) has been identified, that an ISO 14971:2019 risk management file has been opened, and that the clinical domain has been sufficiently defined to permit meaningful threshold derivation. Completing this checklist confirms that the organization possesses the regulatory and clinical context necessary to configure AEGIS in a manner that is both clinically justified and defensible under applicable law; no module configuration should proceed until all items in this section are satisfied.

Table S1: Pre-Implementation Requirements Checklist

☐	Requirement	Evidence/Documentation
**Regulatory Pathway Identification**		
☐	Target regulatory jurisdiction(s) identified	________________________
☐	FDA PCCP pathway confirmed (if US market)	510(k) number or pre-submission ref: ____________
☐	EU MDR classification determined (if EU market)	Class: ______ Rule: ______
☐	EU Artificial Intelligence (AI) Act risk category confirmed	☐ High-risk ☐ Limited risk
☐	Notified Body (NB) identified (if applicable)	NB name: ____________
**Risk Management Foundation**		
☐	ISO 14971:2019 risk management file established	File reference: ____________
☐	Hazard analysis completed	Document ref: ____________
☐	Risk-benefit analysis documented	Document ref: ____________
☐	Severity classifications defined	S1–S5 scale documented: ☐ Yes
☐	Threshold traceability to risk assessment	Mapping table completed: ☐ Yes
**Clinical Domain Definition**		
☐	Intended use statement finalized	Document ref: ____________
☐	Target patient population defined	Population: ________________________
☐	Clinical workflow integration documented	Workflow diagram ref: ____________
☐	Primary clinical endpoints identified	Endpoints: ________________________
**Stakeholder Identification**		
☐	Clinical expert(s) assigned	Name(s): ____________
☐	Risk manager assigned	Name: ____________
☐	Regulatory specialist assigned	Name: ____________

☐	Requirement	Evidence/Documentation
☐	Quality management representative assigned	Name: ________________
☐	Escalation contacts documented	Contact list ref: ________________

S1.2 DARM Configuration Checklist

The DARM governs the controlled ingestion of new observations and the structured maintenance of three dataset splits—the static Golden dataset ($\mathcal{D}_G$), the dynamic Training dataset ($\mathcal{D}_{T,k}$), and the Drifting dataset ($\mathcal{D}_{D,k}$)—ensuring that every data point has a documented, auditable status before it can influence model retraining or governance decisions. This checklist verifies that patient-safe splitting is implemented (preventing data leakage across splits at the patient level), that data quality gates are defined (missing-data thresholds, outlier detection, label verification), and that provenance metadata is captured for every incoming batch. Organizations should complete this section in conjunction with their data governance and informatics teams, as any deficiency in DARM configuration—particularly around dataset separation or label provenance—will compromise the integrity of the Golden dataset and therefore invalidate the fixed performance reference comparisons on which CDM decisions depend.

Table S2: DARM Configuration Checklist

☐	Requirement	Specification
**Data Modality Specification**		
☐	Data type defined	☐ Tabular ☐ Imaging ☐ Time-series ☐ Text
☐	Feature dimensionality documented	Number of features: ________
☐	Data format standardized	Format: ________________
☐	Preprocessing pipeline documented	Pipeline ref: ________________
**Patient-Safe Splitting**		
☐	Patient ID field identified	Field name: ________________
☐	Patient-safe split function implemented	Function ensures all samples from one patient in same split: ☐ Yes
☐	Split ratios defined	Training: ________% Golden: ________% Drifting: ________%
☐	Stratification variables identified	Variables: ________________________________
**Dataset Definitions**		
☐	Golden dataset ($\mathcal{D}_G$) specification	Size: ________ Static: ☐ Yes
☐	Training dataset ($\mathcal{D}_{T,k}$) specification	Initial size: ________ Dynamic: ☐ Yes
☐	Drifting dataset ($\mathcal{D}_{D,k}$) specification	Batch size: ________ Cadence: ________________
☐	Dataset versioning implemented	Version control: ☐ Yes
**Data Quality Gates**		
☐	Missing data threshold defined	Max missing: ________%
☐	Outlier detection implemented	Method: ________________
☐	Label quality verification process	Process ref: ________________
☐	Data exclusion criteria documented	Criteria ref: ________________
**Provenance Metadata Schema**		
☐	Source system tracked	Field: ________________
☐	Collection window tracked	Field: ________________
☐	Labeling method tracked	Field: ________________
☐	Reviewer identifiers tracked	Field: ________________
☐	Timestamps tracked	Field: ________________
☐	Dataset hash/fingerprint implemented	Algorithm: ________________

S1.3 MMM Configuration Checklist

The MMM is responsible for calculating performance metrics on both the static Golden dataset and the dynamic Drifting dataset at each iteration, maintaining a historical Key Performance Indicator (KPI) database, and providing the quantitative inputs on which the CDM bases its deployment decisions. This checklist verifies that all required performance metrics (sensitivity, specificity, Area Under the ROC Curve (ROC-AUC), MLcps), drift detection parameters (Kolmogorov-Smirnov (KS) test with appropriate multiple-testing correction, minor- and major-drift thresholds), and TAI dimensions (explainability via SHAP, fairness, calibration) have been specified with documented clinical rationale. Organizations should ensure that the MMM configuration directly maps to the threshold values established in Section S2.3: any metric monitored by the MMM but not included in the threshold specification table represents an unverified safety gap.

Table S3: MMM Configuration Checklist

☐	Requirement	Specification
**Primary Performance Metrics**		
☐	Primary metric 1 selected	Metric: ________ Threshold: ________
☐	Primary metric 2 selected	Metric: ________ Threshold: ________
☐	Primary metric 3 selected	Metric: ________ Threshold: ________
☐	Composite metric (MLcps) configured	Enabled: ☐ Yes Threshold: ________
☐	Clinical rationale documented for each	Document ref: ________
**MLcps Weight Configuration**		
☐	Sensitivity weight	Weight: ________ (raw)
☐	Specificity weight	Weight: ________ (raw)
☐	ROC-AUC weight	Weight: ________ (raw)
☐	Balanced accuracy weight	Weight: ________ (raw)
☐	Other metric weights (if applicable)	Metric: ________ Weight: ________
☐	Weight convention documented	Raw weights (MLcps package normalizes internally): ☐ Yes
☐	Weight rationale documented	Risk file ref: ________
**Fairness Metrics**		
☐	Protected attributes identified	Attributes: ________
☐	Fairness metric(s) selected	☐ Demographic parity ☐ Equalized odds ☐ Other
☐	Fairness threshold defined	Max disparity: ________
☐	Subgroup minimum sample size	Min N per group: ________
**Drift Detection Configuration**		
☐	Drift detection method selected	☐ KS test ☐ Population Stability Index (PSI) ☐ Maximum Mean Discrepancy (MMD) ☐ Other: ________
☐	Reference distribution defined	Reference: ☐ Golden ☐ Last approved ☐ Other
☐	Features for drift monitoring identified	Number: ________ Selection method: ________
☐	Minor drift threshold	Threshold: ________
☐	Major drift threshold	Threshold: ________
☐	Multiple testing correction	Method: ☐ Benjamini-Hochberg (BH) ☐ Bonferroni ☐ None
☐	Significance level (α)	$\alpha =$ ________
**TAI Dimensions**		
☐	Explainability metric configured	Method: ☐ SHAP ☐ Local Interpretable Model-Agnostic Explanations (LIME) ☐ Other
☐	Explainability threshold defined	Threshold: ________

☐	Requirement	Specification
☐	Robustness metric configured	Method: __________________
☐	Robustness threshold defined	Threshold: _________
☐	Calibration metric configured	☐ Brier score ☐ Expected Calibration Error (ECE) ☐ Other
☐	Calibration threshold defined	Threshold: _________
**Historical KPI Database**		
☐	KPI storage mechanism implemented	Storage: ☐ Database ☐ File ☐ Other
☐	Retention policy defined	Retention: __________________
☐	Query/retrieval functions implemented	Functions tested: ☐ Yes

S1.4 CDM Configuration Checklist

The CDM produces the composite governance output at each iteration: a deployment decision (APPROVE, CONDITIONAL APPROVAL, CLINICAL REVIEW, or REJECT) for the new candidate model, evaluated against the Golden dataset, together with an independent PMS ALARM signal assessing the continued safety of the currently released model on the incoming Drifting batch. This checklist verifies that all four deployment decision categories and all three ALARM conditions (A1: safety floor breach; A2: regression from release baseline; A3: major distributional shift) have been defined, that the priority-ordered evaluation hierarchy is correctly implemented (P1 Safety → P2 Performance/Gold Standard → P3 Drift/TAI → P4 APPROVE; ALARM evaluated in parallel), and that each threshold carries documented traceability to the ISO 14971:2019 hazard analysis. Special attention should be given to the safety boundary documentation items: the CDM's ability to prevent silent model degradation depends critically on the alignment between the deployment safety performance floor (P^{fail}) and the hazards identified in the risk management file.

Table S4: CDM Configuration Checklist

☐	Requirement	Specification
**Four Deployment Decision Thresholds + PMS ALARM Signal**		
☐	REJECT threshold(s) defined	Critical safety threshold: __________________
☐	CLINICAL REVIEW threshold(s) defined	Buffer zone bounds: __________________
☐	CONDITIONAL APPROVAL threshold(s) defined	Minor drift/TAI threshold: __________________
☐	APPROVE criteria defined	All-pass criteria documented: ☐ Yes
☐	PMS ALARM A1 threshold defined (P_{PMS})	Released model safety floor: __________________
☐	PMS ALARM A2 margin defined (τ^{PMS})	Released model regression margin: __________________
☐	PMS ALARM A3 threshold defined	Major drift threshold (e.g., >0.90): __________________
☐	ALARM triggers documented as parallel PMS signal	Fires independently of primary decision: ☐ Yes
**Gold Standard Comparison Parameters**		
☐	Gold standard baseline storage implemented	Storage mechanism: __________________
☐	Acceptance margin (τ^{buf}) for Sensitivity	τ^{buf} = _________
☐	Acceptance margin (τ^{buf}) for Specificity	τ^{buf} = _________
☐	Acceptance margin (τ^{buf}) for ROC-AUC	τ^{buf} = _________
☐	Margin derivation documented	Risk file ref: __________________
**Acceptance Criteria Approach**		
☐	Approach selected	☐ Deterministic thresholds ☐ Confidence Interval (CI)-based testing
☐	Confidence level (if CI-based)	CI level: _________%
☐	Bootstrap iterations (if CI-based)	B = _________
☐	Non-inferiority margin (if CI-based)	δ_m = _________

☐	Requirement	Specification
☐	Sample size justification documented	Document ref: ________________
**Decision Logic Implementation**		
☐	Boolean function $f(C_1, C_2, \ldots, C_N)$ defined	Function documented: ☐ Yes
☐	Priority order implemented	New model: Safety (P1) → Performance/Gold std (P2) → Minor Drift/TAI (P3) → Approve (P4); PMS ALARM evaluated in parallel (A1–A3): ☐ Yes
☐	Decision logging implemented	Log fields: ________________________________
☐	Trigger reason captured	Reason field: ☐ Yes
☐	Decision confidence calculated (if CI-based)	Confidence field: ☐ Yes
**Safety Boundary Documentation**		
☐	Safety boundaries linked to ISO 14971 hazards	Traceability matrix: ☐ Yes
☐	Severity level for each threshold documented	Mapping complete: ☐ Yes
☐	Clinical justification for safety thresholds	Document ref: ________________

S1.5 Regulatory Documentation Checklist

AEGIS is an internal governance and post-market monitoring infrastructure that can support—but does not constitute—regulatory change control; its outputs gain regulatory relevance only when systematically documented and mapped to the applicable legal requirements. This checklist verifies that the three components of an FDA PCCP (Description of Modifications, Modification Protocol, and Impact Assessment) are each addressed by the corresponding AEGIS modules (DARM protocols and CDM decision logic; MMM metric procedures; fixed performance reference comparison mechanism), and that EU MDR/AI Act obligations—including PMS under Article 83, predetermined change criteria under Article 43(4), and vigilance under Article 89—are covered by the configured infrastructure. Organizations preparing regulatory submissions should complete this section together with their regulatory affairs team to confirm that the AEGIS configuration record constitutes an audit-ready artifact; jurisdictions may impose additional requirements not captured here, and regulatory counsel should be consulted before submission.

Table S5: Regulatory Documentation Checklist

☐	Requirement	Evidence/Documentation
**FDA PCCP Three-Component Mapping**		
☐	Description of Modifications documented	DARM protocols + CDM decision logic mapped: ☐ Yes
☐	Modification Protocol documented	MMM metric calculation procedures mapped: ☐ Yes
☐	Impact Assessment documented	Gold standard comparison mechanism mapped: ☐ Yes
☐	PCCP scope boundaries clearly defined	Boundaries document ref: ________________
**EU MDR/AI Act Alignment**		
☐	Article 83 (PMS) requirements addressed	PMS plan ref: ________________
☐	Article 43(4) predetermined change criteria met	Criteria documentation: ________________
☐	Article 89 vigilance procedures defined	ALARM escalation mapped: ☐ Yes
☐	AI Act Article 9 (Risk Management) addressed	Risk system ref: ________________
☐	AI Act Article 13 (Transparency) addressed	Transparency documentation: ________________

☐	Requirement	Evidence/Documentation
**Audit Trail Specifications**		
☐	Audit trail fields defined	Fields documented: ☐ Yes
☐	Timestamp format standardized	Format: ____________
☐	Immutability mechanism implemented	Mechanism: ____________
☐	Retention period defined	Period: ____________
☐	Access controls implemented	Role-based access: ☐ Yes
**Escalation Pathway Documentation**		
☐	ALARM response protocol documented	Protocol ref: ____________
☐	REJECT response protocol documented	Protocol ref: ____________
☐	CLINICAL REVIEW workflow documented	Workflow ref: ____________
☐	Regulatory notification triggers defined	Triggers: ________________________
☐	Escalation contacts documented	Contact list: ____________

S1.6 CAPA and Vigilance Integration Checklist

Note: This checklist ensures AEGIS decision outputs integrate with existing QMS processes per ISO 13485 and MDR vigilance requirements. Templates adapted from OpenRegulatory (openregulatory.com).

AEGIS governance decisions—particularly ALARM and REJECT outputs—do not resolve safety concerns in isolation; they are signals that must be routed into the organization's existing quality management and vigilance processes to produce corrective action. This checklist verifies that ALARM and REJECT events are connected to CAPA initiation procedures per ISO 13485, that the escalation pathway from an ALARM signal to the Person Responsible for Regulatory Compliance (PRRC) is documented, and that MDR vigilance obligations are operationally linked to the CDM's output stream. Completing this section confirms that AEGIS is integrated into—rather than operating in parallel to—the organization's QMS, which is a prerequisite for the infrastructure to function as a genuine post-market surveillance instrument rather than a standalone analytics tool.

Table S6: CAPA and Vigilance Integration Checklist

☐	Requirement	Evidence/Documentation
**CAPA Process Integration (ISO 13485 Section 8.5.2/8.5.3)**		
☐	CAPA initiation triggers defined for ALARM	CAPA opened automatically: ☐ Yes ☐ Manual
☐	CAPA initiation triggers defined for REJECT	CAPA opened automatically: ☐ Yes ☐ Manual
☐	Root cause analysis method specified	☐ Five Whys ☐ Fishbone ☐ Other: ____________
☐	CAPA list/tracking system identified	System: ____________
☐	CAPA closure criteria defined	Criteria: ____________
☐	CAPA effectiveness verification process	Process ref: ____________
**Vigilance Process Integration (MDR Art. 87-92)**		
☐	Serious incident criteria defined	Definition ref: ____________
☐	ALARM-to-Vigilance escalation pathway	☐ Automatic referral to PRRC ☐ Manual assessment
☐	Reporting timescales documented	Death/serious: ______hrs Other: ______days
☐	Competent authority contacts documented	Authority: ____________
☐	Field Safety Corrective Action (FSCA) process	Standard Operating Procedure (SOP) ref: ____________

☐	Requirement	Evidence/Documentation
☐	NB notification triggers	Triggers documented: ☐ Yes
**Trend Analysis (MDR Art. 88)**		
☐	Trend analysis method for non-serious incidents	Method: ____________________
☐	Statistical significance criteria	Criteria: ____________________
☐	Baseline comparison (from risk management file)	Baseline documented: ☐ Yes
☐	Trend reporting triggers	Threshold: ____________________
☐	AEGIS drift detection integrated with trend analysis	Integration documented: ☐ Yes
**PMS Report/PSUR Structure (MDR Art. 85-86)**		
☐	Report type determined	☐ PMS Report (Class I) ☐ PSUR (Class IIa+)
☐	Update interval defined	Interval: ____________________
☐	AEGIS outputs mapped to report sections	Mapping documented: ☐ Yes
☐	Benefit-risk conclusion process	Process ref: ____________________

S2 AEGIS Configuration Template

Instructions: Complete all sections of this template to document your AEGIS infrastructure instantiation. This template serves as both a configuration record and regulatory submission artifact. Replace bracketed placeholder text with your specific values.

This template provides a structured, fillable record for documenting all domain-specific parameter choices made when instantiating AEGIS for a particular clinical application. Where the main manuscript describes the AEGIS architecture in domain-agnostic terms, this template captures the specific values—thresholds, weights, baselines, and Boolean conditions—that make the infrastructure operational for a given device, dataset, and regulatory context. Each completed template serves simultaneously as an internal configuration record and as the primary artifact for inclusion in a PCCP submission, EU MDR technical file, or internal QMS change-control record; Appendices A and B provide fully worked examples for the sepsis prediction and brain tumor segmentation instantiations described in the main manuscript.

S2.1 Device/Application Identification

This section anchors the AEGIS configuration to a specific, legally identified medical device or software system, establishing the scope boundary within which all subsequent governance parameters apply. Regulatory frameworks are device-specific: the applicable FDA classification pathway, EU MDR risk class, and EU AI Act category determine which conformity assessment procedures and post-market obligations govern the device, and therefore which PCCP components or MDR technical documentation requirements the AEGIS configuration must satisfy. Organizations should complete this section first, before deriving any thresholds or configuring any module, because the device's intended use and risk classification directly constrain the clinical performance standards to which the deployment safety performance floor (P^{fail}) must be anchored.

Table S7: Device and Application Identification Details

**Field**	**Value**
Device/System Name	____________________
Version	__________
Intended Use	____________________
	____________________
Target Population	____________________
FDA Classification	☐ Class I ☐ Class II ☐ Class III
510(k) Reference (if applicable)	__________
EU MDR Classification	☐ Class I ☐ Class IIa ☐ Class IIb ☐ Class III
EU AI Act Category	☐ High-risk ☐ Limited risk ☐ Minimal risk
NB (if applicable)	__________

S2.2 Infrastructure Instantiation Parameters

AEGIS is designed as a domain-agnostic wrapper: the three-module architecture (DARM, MMM, CDM) and the priority-ordered evaluation mechanism are invariant across clinical applications, while the data modality, model architecture, primary performance metrics, MLcps weighting, drift detection method, *the set of active decision categories*, and *the Boolean conditions that define each category* are configured per instantiation. The decision logic is therefore domain-configured rather than invariant: the brain tumor instantiation, for example, redefines APPROVE/REJECT against P_G^{rel} and deactivates CONDITIONAL APPROVAL and CLINICAL REVIEW (Section S4.2). This section documents those instantiation-level choices in a structured format that makes explicit both the domain-specific configuration values and the generic infrastructure definitions they override or specialize, providing a clear record of what has been adapted from the reference architecture described in the main manuscript. A complete entry in this table, alongside the threshold specification in Section S2.3, constitutes the core technical record for demonstrating that AEGIS has been rigorously instantiated for the intended clinical use.

Table S8: Infrastructure Instantiation Parameters Template

**Parameter**	**Infrastructure Definition**	**Your Configuration**
Data Modality	Any supervised learning data	________________
Model Architecture	Agnostic (wrapper)	________________
Primary Metrics	Domain-configurable	________________
MLcps Weighting	User-defined via risk assessment	See Section S2.4
Drift Detection	Pluggable method	________________
Clinical Thresholds	Risk-assessment derived	See Table S10
Safety Actions	ALARM/REJECT triggers	Same CDM logic

S2.3 Threshold Specification Table

Critical: All thresholds must be traceable to documented risk assessment per ISO 14971:2019. Complete the “Hazard Reference” column to establish this traceability.

The deployment decisions produced by the CDM are entirely determined by the thresholds entered in this table; a poorly justified threshold is therefore a governance risk and a potential regulatory liability, not merely a configuration parameter. Every threshold must be traceable to a documented ISO 14971:2019 hazard, as indicated by the mandatory "Hazard Reference" column: this traceability is what converts a numerical value into a defensible clinical safety boundary and satisfies the risk-management documentation requirements of both the FDA PCCP framework and MDR Annex I. Organizations should complete this table in collaboration with their risk manager and clinical expert(s), working directly from the hazard analysis outputs produced during Section S1.1, and should treat any row with an empty "Hazard Reference" field as a compliance gap requiring resolution before deployment.

Table S9: Threshold Specification with Regulatory Traceability

Metric	Threshold	Clinical Rationale	ISO 14971 Hazard Ref	Decision if Failed
**Safety Boundaries (REJECT triggers)**				
Sensitivity	≥ ______	__________	H-______	REJECT
__________	≥ ______	__________	H-______	REJECT
__________	≥ ______	__________	H-______	REJECT
**Performance Boundaries (CLINICAL REVIEW)**				
Sensitivity	≥ ______	__________	H-______	CLIN. REV.
Specificity	≥ ______	__________	H-______	CLIN. REV.
ROC-AUC	≥ ______	__________	H-______	CLIN. REV.
MLcps	≥ ______	__________	H-______	CLIN. REV.
__________	≥ ______	__________	H-______	CLIN. REV.
**Drift Boundaries**				
Minor Drift	> ______	Enhanced monitoring	—	COND. APPR.
Major Drift	> ______	Distribution shift	—	ALARM
**TAI Boundaries**				
Fairness	≥ ______	__________	—	COND. APPR.
Explain.	≥ ______	__________	—	COND. APPR.
Robustness	≥ ______	__________	—	COND. APPR.
**APPROVE Criteria (all must pass)**				
All safety	Pass	—	—	—
All perf.	Pass	—	—	—
Drift	Not detected	—	—	—
All TAI	Pass	—	—	—
Gold std.	Pass	—	—	—

S2.4 MLcps Weight Configuration

The MLcps integrates multiple domain-specific performance metrics into a single composite indicator that the CDM can evaluate against a unified governance threshold, enabling risk-weighted

multi-metric assessment without requiring separate threshold conditions for each individual metric. The raw weights assigned in this section reflect the relative clinical importance of each metric as derived from the ISO 14971:2019 hazard analysis: a metric whose failure is linked to a high-severity hazard (e.g., sensitivity for missed sepsis cases) should receive proportionally higher weight than a metric linked to a lower-severity hazard. Organizations should document the weight derivation method (clinical expert consensus, risk-weighted analysis, or literature-based) and retain the supporting evidence, as MLcps weights constitute a configuration decision that may require justification in a PCCP submission or during a notified body audit.

Table S11: MLcps Weight Configuration Template

**Metric**	**Weight (Raw)**	**Clinical Justification**
Sensitivity	________	________________________________
Specificity	________	________________________________
ROC-AUC	________	________________________________
Balanced Accuracy	________	________________________________
________________	________	________________________________
________________	________	________________________________

Note: The official MLcps package normalizes weights internally. Raw weights need not sum to 1.0.

Weight Derivation Method:
☐ Clinical expert consensus ☐ Risk-weighted analysis ☐ Literature-based ☐ Other: ________________

Documentation Reference: ________________________________

S2.5 Gold Standard Baseline Documentation

The fixed performance reference (P^{ref}) recorded in this section is the invariant operational benchmark against which all subsequent model iterations are evaluated: once established from the initial approved model's performance on the static Golden dataset ($\mathcal{D}_G$), it does not change between retraining cycles unless a deliberate, documented re-baselining event occurs under human adjudication. This section captures the P^{ref} values, the acceptance margins (τ^{safe}) that define the deployment safety performance floor, and the lower bounds that trigger CDM escalation decisions, together with the dataset metadata (size, hash, and validation method) required to demonstrate the baseline's integrity. The completed table constitutes a mandatory reference document for the fixed performance reference comparison mechanism and should be retained under version control, as any change to P^{ref} represents a significant configuration event that may have regulatory implications.

Table S12: Initial Deployment Metrics (Gold Standard Baseline)

**Metric**	**Baseline Value**	**Acceptance Margin (τ^{buf})**	**Lower Bound**
Sensitivity	________	± ________	________
Specificity	________	± ________	________
ROC-AUC	________	± ________	________
MLcps	________	± ________	________
________	________	± ________	________
________	________	± ________	________

Baseline Establishment Details:

Date Established: ________________
Model Version: ________________
Golden Dataset Size: ________________
Golden Dataset Hash: ________________
Validation Method: ☐ Hold-out ☐ Cross-val. ☐ External
Regulatory Approval Ref: ________________

CI Configuration (if applicable):

Confidence Level: ________%
Bootstrap Iterations: ________
Non-inferiority Margin (δ_m): ________
Random Seed: ________

S2.6 Decision Logic Specification

This section translates the conceptual CDM priority hierarchy—described formally in the main manuscript—into an executable, institution-specific Boolean specification that can be directly implemented in code and audited by regulators. The pseudocode block in Figure S1 shows the complete two-track architecture with sepsis instantiation values pre-filled as a worked example; organizations should replace these values with their domain-specific thresholds (as specified in Section S2.3) and complete the Boolean function specification table (Table S13) to document the exact logical conditions governing each of the four deployment decision categories and the three parallel ALARM conditions. The completed specification serves as the authoritative record of the CDM's decision logic and should be version-controlled alongside the software implementation to maintain alignment between the documented governance specification and the deployed code. The CDM evaluates conditions in the following priority order. The thresholds shown are the worked values from the sepsis instantiation (Appendix A); the trustworthy-AI gate is illustrative and was not triggered in this proof-of-concept.

Table S13: Specification of Threshold Values and Boolean Function

Your Specific Threshold Values: *Critical Safety Thresholds (REJECT triggers — P1):*
`sens_critical` ________

Performance / Gold Standard Thresholds (CLINICAL REVIEW triggers — P2):
`sens_performance` ________
`spec_performance` ________
`gold_std_tolerance` (τ^{buf}) ________

Minor Drift and TAI Thresholds (CONDITIONAL APPROVAL— P3):
`minor_drift_low` ________
`minor_drift_high` ________
`TAI_threshold` ________

PMS ALARM Thresholds (Track 2 — parallel, released model):
`P_PMS` (A1: safety floor) ________
`delta` (A2: regression margin) ________
`major_drift_threshold` (A3) ________

Boolean Function Specification:
$f(C_1, C_2, \ldots, C_N) =$ ________________________________

where:

- $C_1 =$ ________________
- $C_2 =$ ________________
- $C_3 =$ ________________
- $C_N =$ ________________

S2.7 Regulatory Pathway Mapping

A completed AEGIS configuration has regulatory value only insofar as its components can be mapped to the specific documentation requirements of the applicable regulatory framework; this section makes that mapping explicit and auditable. The two tables in this section—one for the FDA PCCP three-component structure (Description of Modifications, Modification Protocol, Impact Assessment) and one for EU MDR/AI Act obligations (Articles 83, 43(4), 89; AI Act Articles 9 and 13)—identify which AEGIS module or output satisfies each regulatory requirement, and provide a "Your Documentation Reference" column for organizations to link each mapping to the corresponding section of their regulatory submission package. This section does not constitute legal advice or a guarantee of regulatory acceptance; its purpose is to assist organizations in structuring their documentation and in identifying any regulatory obligations for which the AEGIS configuration does not yet provide a documented artifact.

Table S14: FDA PCCP Alignment

**PCCP Component**	**AEGIS Implementation**	**Your Documentation Ref**
Description of Modifications	DARM protocols + CDM decision logic	________________
Modification Protocol	MMM metric procedures	________________
Impact Assessment	Gold standard comparison	________________

Table S15: EU MDR/AI Act Alignment

Requirement	AEGIS Implementation	Your Documentation Ref
Art. 83 (PMS)	MMM continuous monitoring	________________
Art. 43(4) (Predetermined)	Four-category deployment taxonomy + parallel PMS ALARM signal	________________
Art. 89 (Vigilance)	ALARM escalation	________________
AI Act Art. 9 (Risk)	ISO 14971 traceability	________________
AI Act Art. 13 (Transparency)	Explainability metrics	________________

S2.8 Risk Acceptability Matrix

Purpose: This matrix establishes risk acceptability criteria per ISO 14971:2019 and links AEGIS decision thresholds to severity/probability classifications. Adapted from OpenRegulatory templates (openregulatory.com).

The CDM's deployment decision categories (APPROVE, CONDITIONAL APPROVAL, CLINICAL REVIEW, REJECT) and the PMS ALARM signal are operationalizations of risk acceptability judgments: each category implicitly encodes a determination that the associated risk level is either acceptable, requires mitigation, or is unacceptable. This section makes those risk-acceptability judgments explicit and traceable to ISO 14971:2019 by providing a standardized severity-probability matrix (Tables S17–S19) and mapping each CDM decision category to its corresponding risk zone (Table S20). Organizations should use this section to confirm that the threshold assignments made in Section S2.3 are consistent with the risk-benefit determinations in their risk management file, and to provide auditors with evidence that the governance infrastructure's decision boundaries reflect a principled and documented risk analysis rather than arbitrary numerical choices.

Table S16: Estimated Product Usage

Estimated Product Lifetime:	________________years
Estimated Maximum Event Count:	________________predictions/classifications over lifetime
PMS/Post-Market Clinical Follow-up (PMCF) Surveillance Period:	________________months

Table S17: Severity Classifications

Level	Category	Definition	Example for AI/Machine Learning (ML) Device
S1	Negligible	No injury or impact on health	Model confidence displayed incorrectly
S2	Minor	Temporary discomfort, no intervention	Unnecessary follow-up test ordered
S3	Serious	Injury requiring medical intervention	Delayed diagnosis leading to extended treatment
S4	Critical	Permanent impairment or life-threatening	Missed critical condition (e.g., sepsis)
S5	Catastrophic	Death	Undetected condition leading to death

Table S18: Probability Classifications

Level	Category	Definition	Occurrence Rate
P1	Incredible	Cannot occur over product lifetime	$< 10^{-6}$ per use
P2	Improbable	Unlikely but possible	10^{-6} to 10^{-4}
P3	Remote	May occur rarely	10^{-4} to 10^{-2}
P4	Occasional	Expected to occur sometimes	10^{-2} to 10^{-1}
P5	Frequent	Expected to occur regularly	$> 10^{-1}$ per use

Table S19: Risk Acceptability Matrix

Prob \ Sev	S1	S2	S3	S4	S5
P5	ALARA	Unacceptable	Unacceptable	Unacceptable	Unacceptable
P4	Acceptable	ALARA	Unacceptable	Unacceptable	Unacceptable
P3	Acceptable	Acceptable	ALARA	Unacceptable	Unacceptable
P2	Acceptable	Acceptable	Acceptable	ALARA	Unacceptable
P1	Acceptable	Acceptable	Acceptable	Acceptable	ALARA

Legend:

- Acceptable — Risk acceptable without further reduction
- ALARA — As Low As Reasonably Achievable (ALARA); reduce if practical
- Unacceptable — Risk reduction required; justify if not possible
- Unacceptable — Risk reduction mandatory; consider benefit-risk only if unavoidable

Table S20: Mapping AEGIS Decisions to Risk Acceptability

AEGIS Decision	Risk Zone	Implication
REJECT	Unacceptable (Red)	Model creates unacceptable risk; cannot deploy
ALARM (PMS Signal)	Unacceptable (Orange)	PMS signal for released model; co-issued with any primary decision; investigation of released model required
CLINICAL REVIEW	ALARA boundary	Near threshold; human assessment of risk-benefit
CONDITIONAL APPROVAL	Acceptable/ALARA	Acceptable with enhanced monitoring
APPROVE	Acceptable (Green)	Risk within acceptable bounds

S3 AEGIS Implementation Guide

This guide provides step-by-step operational instructions for deploying AEGIS across the six implementation phases illustrated in the Infrastructure Adoption Workflow (Figure S2): risk assessment, threshold derivation, module configuration, validation, regulatory documentation, and deployment. Where Sections S1 and S2 provide checklists and templates for recording configuration decisions, this guide explains how to make those decisions—from conducting an ISO 14971:2019 hazard analysis and deriving thresholds from its outputs, to constructing the CDM Boolean function, integrating TAI dimensions, structuring regulatory submissions, and maintaining operationally through post-deployment monitoring and PSUR reporting. Organizations implementing for the first time should work through this guide sequentially before attempting to complete the S1 checklist or the S2 configuration template.

S3.1 Infrastructure Adoption Workflow

The workflow diagram in Figure S2 organizes the implementation process into six sequential phases that reflect the logical dependencies between risk analysis, technical configuration, and regulatory submission. Each phase must be completed before the next begins: for example, threshold derivation (Phase 2) cannot proceed without a completed hazard analysis (Phase 1), and regulatory documentation (Phase 5) cannot be finalized without validated decision logic (Phase 4). Organizations should use this workflow as a project management reference to sequence implementation activities, assign responsibilities across clinical, regulatory, and engineering teams, and track progress toward an audit-ready, deployable configuration.

S3.2 Deriving Thresholds from Risk Assessment

The numerical thresholds that govern deployment decisions are not configurable free parameters to be set by engineering convenience; they are risk-management outputs that must be derived from documented hazard analyses conducted under ISO 14971:2019. This section describes the five-step process for translating a hazard analysis into defensible, traceable threshold values—from hazard identification and severity classification through metric mapping and threshold setting to explicit traceability documentation—and provides a fully worked example using the sepsis prediction instantiation described in the main manuscript. The output of this process is the completed Section S2.3 Threshold Specification Table, which should reference the hazard analysis document produced in Phase 1 of the adoption workflow; any threshold that cannot be linked to a specific, documented hazard represents a gap in the risk management file.

S3.2.1 Step-by-Step Process

1. **Identify Hazards:** List all hazards associated with model predictions (false positives, false negatives, delayed predictions, etc.)
2. **Assess Severity:** Assign severity levels (S1–S5) per ISO 14971:2019:
   - S1: Negligible — No injury
   - S2: Minor — Temporary discomfort
   - S3: Serious — Injury requiring intervention
   - S4: Critical — Permanent impairment
   - S5: Catastrophic — Death
3. **Map to Metrics:** For each hazard, identify the metric(s) that would detect or prevent it

4. **Set Thresholds:** Based on severity and probability:
   - S5 hazards → Conservative thresholds, REJECT boundary
   - S3–S4 hazards → Performance thresholds, CLINICAL REVIEW boundary
   - S1–S2 hazards → Monitoring thresholds, CONDITIONAL APPROVAL boundary
5. **Document Traceability:** Create explicit links from each threshold to its originating hazard

S3.2.2 Worked Example: Sepsis Prediction

Hazard: Missed sepsis case (false negative)
Severity: S5 (Catastrophic) — Delayed treatment can result in patient death
Metric: Sensitivity (true positive rate)
Threshold Derivation:

- Literature review: Pooled sensitivity for early warning scores is 0.67 (95% CI: 0.54–0.77)
- Clinical consensus: Minimum acceptable sensitivity for deployment is 0.65
- Critical safety boundary: Below 0.65 is unacceptable → REJECT
- Performance boundary: Below 0.66 requires clinical review → CLINICAL REVIEW

Traceability: Sensitivity $\geq 0.65 \leftarrow$ H-001 (Missed Sepsis) ← Risk File v2.3, Section 4.2.1

S3.3 Decision Logic Configuration

The CDM's governance decisions emerge from a strictly ordered priority hierarchy of Boolean conditions, formalized in Algorithm S1: safety violations are evaluated first (P1), followed by performance buffer and fixed-reference regression checks (P2), then minor drift and TAI conditions (P3), with APPROVE returned only when all preceding conditions are satisfied (P4). This section explains how to instantiate this hierarchy for a specific clinical application by selecting the appropriate evaluation conditions, filling in the domain-specific threshold values, and encoding the resulting logic as a compound Boolean function. A correctly configured decision logic specification—completed as Table S13 and supported by Algorithm S1—is the primary technical artifact that regulatory reviewers will examine to assess whether the CDM's change-control behavior is deterministic, auditable, and aligned with the pre-specified risk thresholds in the PCCP.

S3.3.1 Decision Hierarchy

As illustrated in Figure S3, the CDM implements a strict priority hierarchy ensuring safety-critical conditions are evaluated first.

S3.3.2 Constructing the Boolean Function

As shown in Algorithm S1, the compound Boolean function $f(C_1, C_2, \ldots, C_N)$ formalizes the decision logic.

Algorithm S1: CDM Two-Track Decision Logic: Primary Deployment Decision (P1–P4) + Parallel PMS ALARM Signal (A1–A3)

Input : M: metrics on $\mathcal{D}_G$ (sens., spec., AUC, MLcps)
D: drift results (score, minor/major flags)
T: TAI indicators (fairness, explainability)
$\mathcal{S}_{\text{TAI}}^{\text{thr}}$: Threshold for TAI composite score
G: gold standard comparison result
R: released model performance on $\mathcal{D}_{D,k}$
P_G^{rel}: released model performance at time of release

Output: Primary decision label; PMS ALARM signal (if fired); trigger reasons

`// -- TRACK 1: New Model Deployment Decision (priority-ordered) --`

`// Priority 1: Safety boundary`
$C_1 \leftarrow (M.\text{sensitivity} < P^{\text{fail}})$;
if C_1 **then**
  return **REJECT**, *"Safety boundary violation"*
end

`// Priority 2: Performance buffer zone or gold standard regression`
$C_2 \leftarrow (M.\text{sensitivity} < \theta_{\text{perf,sens}})$ **or** $(M.\text{specificity} < \theta_{\text{perf,spec}})$ **or** $(G = \text{FAIL})$;
if C_2 **then**
  return **CLINICAL REVIEW**, *"Borderline performance or gold standard regression"*
end

`// Priority 3: Minor drift or TAI concerns`
$C_3 \leftarrow (D.\text{minor\_drift})$ **or** $(T.\text{fairness} < \mathcal{S}_{\text{TAI}}^{\text{thr}})$ **or** $(T.\text{explainability} < \mathcal{S}_{\text{TAI}}^{\text{thr}})$;
if C_3 **then**
  return **CONDITIONAL APPROVAL**, *"Enhanced monitoring required"*
end

`// Priority 4: All criteria satisfied`
primaryDecision $\leftarrow$ **APPROVE**;

`// -- TRACK 2: PMS ALARM Signal (parallel; released model on` $\mathcal{D}_{D,k}$`) --`
$A_1 \leftarrow (R.\text{sensitivity} < P^{\text{PMS}})$;
$A_2 \leftarrow (R.\text{performance} \leq P_G^{\text{rel}} - \tau^{\text{reg}})$;
$A_3 \leftarrow (D.\text{score} > \theta_{\text{major}})$;
if A_1 **or** A_2 **or** A_3 **then**
  alarmFired $\leftarrow$ True;
  `/* Composite output: primaryDecision + ALARM */`
end
return *primaryDecision, alarmFired (if True), trigger reasons*

S3.4 TAI Integration

governance decisions encompass not only classical performance metrics but also TAI dimensions—explainability and fairness—that are increasingly required under both FDA guidance and EU AI Act Article 13. In the CDM, TAI scores function as third-priority (P3) conditions: a model that passes the safety (P1) and performance (P2) checks but exhibits insufficient explainability or demographic fairness receives a CONDITIONAL APPROVAL rather than an APPROVE decision, triggering enhanced monitoring until the concern is resolved. This section provides the calculation procedures for the SHAP-based explainability score and the parity-based fairness score, together with worked numerical examples; organizations should configure these procedures as part of the MMM and ensure that TAI outputs are included in the audit trail alongside performance metrics and drift scores.

S3.4.1 Explainability Configuration (SHAP)

SHAP Explainability Score Calculation:

1. Calculate SHAP values for all features
2. Compute mean absolute SHAP value per feature
3. Rank features by importance
4. Calculate concentration ratio: $\frac{\sum_{i=1}^{k} |SHAP_i|}{\sum_{j=1}^{n} |SHAP_j|}$

   where k = top features (default: 5), n = total features

Interpretation:

- High concentration (>0.80): Model relies on few features (more interpretable)
- Low concentration (<0.50): Distributed importance (less interpretable)

Threshold Guidance:

- Minimum explainability score: 0.55 (CONDITIONAL APPROVAL trigger)
- Recommended target: >0.65 for high-stakes applications

S3.4.2 Fairness Metrics Configuration

Fairness Score Calculation:

1. Identify protected attribute groups (e.g., age groups, sex)
2. Calculate performance metrics per group
3. Compute disparity: $\Delta = |P_{group1} - P_{group2}|$ for each metric
4. Fairness score: $1 - \frac{1}{m} \sum_{i=1}^{m} \Delta_i$

Example (Age-based fairness):

Metric	Age <65	Age ≥65
Sensitivity	0.72	0.68
Specificity	0.85	0.82

$\Delta_{sens} = |0.72 - 0.68| = 0.04$
$\Delta_{spec} = |0.85 - 0.82| = 0.03$
Fairness Score $= 1 - \frac{0.04+0.03}{2} = 0.965$

S3.5 Regulatory Submission Guidance

The configuration artifacts produced by completing Sections S1 and S2 are designed to map directly onto the documentation structures required by the FDA PCCP framework and EU MDR/AI Act technical file, but they require additional structuring and cross-referencing before they constitute a submission-ready package. This section provides a view of the PCCP three-component submission structure (Figure S4) and a mapping table aligning outputs to the relevant Annexes and Articles of the EU MDR (Table S21), enabling organizations to assemble their regulatory documentation by identifying which artifact satisfies each requirement. This guidance reflects the regulatory frameworks as described in the main manuscript and as understood at the time of publication; organizations should verify all regulatory requirements with qualified regulatory counsel, as applicable law, guidance documents, and notified body interpretations may have changed or may vary by jurisdiction.

S3.5.1 FDA PCCP Documentation Structure

The PCCP submission structure is shown in Figure S4.

S3.5.2 EU Technical Documentation Mapping

Table S21: EU MDR Technical Documentation Mapping

MDR Annex II/III Requirement	AEGIS Component	Your Document Reference
Device description	Infrastructure instantiation parameters	________________
Design and manufacturing	DARM + MMM + CDM architecture	________________
Risk management (14971)	Threshold traceability matrix	________________
Clinical evaluation	Validation results, gold standard	________________
PMS plan	MMM monitoring procedures	________________
PMCF plan	Continuous performance tracking	________________
Predetermined change criteria	Four-category deployment taxonomy + parallel PMS ALARM	________________

S3.6 Post-Deployment Operations

Once is activated and the initial gold standard baseline is established, it operates as a continuously running governance engine: at each retraining iteration, the MMM computes metrics on the Golden and Drifting datasets, the CDM evaluates the priority-ordered conditions and produces a composite output, and the audit trail is updated with the full decision record. This section provides the operational standards for sustaining governance across its active lifecycle—defining recommended monitoring cadences for each surveillance dimension, response protocols for each CDM decision category, and the minimum audit trail fields required for regulatory defensibility. Organizations should treat the specifications in this section as the basis for SOPs within their QMS, ensuring that every output is routed to the appropriate organizational response pathway and documented in a manner that supports both internal quality management and external regulatory reporting.

S3.6.1 Monitoring Cadence Recommendations

Table S23: Monitoring Cadence Recommendations

Monitoring Type	Recommended Cadence	Rationale
Performance metrics	Per batch / Daily	Continuous safety assurance
Drift detection	Per batch / Weekly	Early warning before degradation
Fairness assessment	Monthly	Demographic shift detection
Explainability analysis	Per retraining	Model behavior verification
Full infrastructure audit	Quarterly	Regulatory compliance
Gold standard refresh	Annually (or as needed)	Baseline relevance

S3.6.2 Escalation Protocols by Decision Category

The escalation protocols are shown in Figure S5.

S3.6.3 Audit Trail Requirements

Each CDM decision must log the fields listed in Table S24.

Table S24: Audit Trail Requirements

Field	Description
`timestamp`	ISO 8601 format (e.g., 2026-01-15T14:30:00Z)
`iteration_id`	Sequential iteration number
`model_version`	Model identifier/hash
`dataset_hash`	Hash of evaluation dataset
`decision`	Primary: one of APPROVE, CONDITIONAL APPROVAL, CLINICAL REVIEW, REJECT; PMS Signal: ALARM (may co-occur with any primary decision; blank if not fired)
`trigger_reason`	Human-readable reason for decision
`metrics`	JSON object of all evaluated metrics
`thresholds`	JSON object of applied thresholds
`drift_results`	JSON object of drift detection results
`tai_scores`	JSON object of TAI scores
`gold_comparison`	PASS/FAIL with details
`confidence`	Decision confidence (if CI-based)
`reviewer`	System or human reviewer identifier

S3.7 PMS/PSUR Reporting Integration

generates, at each iteration, a machine-readable governance record that contains all the quantitative evidence required to populate the Performance Data, Safety Analysis, and Corrective Actions sections of a PMS Report (for Class I devices) or Periodic Safety Update Report (PSUR, for Class IIa and above devices) under MDR Articles 85 and 86. This section shows how data sources—audit trail timestamps, MMM historical KPIs, CDM decisions, and DARM records—map to each PMS report section, and provides an executive summary template that can be directly populated from the infrastructure's outputs. Integrating outputs into the PMS/PSUR reporting cycle replaces the need for manual data collection and narrative reconstruction, thereby reducing reporting burden while improving the completeness and quantitative rigor of post-market documentation.

Template structure adapted from OpenRegulatory (openregulatory.com).

S3.7.1 AEGIS-to-PMS Report Mapping

Table S25: AEGIS Data Mapping to PMS Report Sections

PMS Report Section	AEGIS Data Source	Content
**1. Summary of PMS Activities**		
Surveillance period	Audit trail timestamps	Start/end dates, iteration count
Data volume	DARM records	Samples processed, patients covered
Monitoring activities	MMM logs	Metrics calculated, drift checks performed
**2. Performance Data**		
Clinical performance	MMM historical KPIs	Sensitivity, Specificity, Positive Predictive Value (PPV), Negative Predictive Value (NPV) trends
Discriminative performance	MMM metrics	ROC-AUC, Area Under the PR Curve (PR-AUC), MLcps over time
Performance vs. baseline	Gold standard comparison	Degradation/improvement analysis
**3. Safety Analysis**		
Serious incidents	CDM decisions (ALARM, REJECT)	Count, root causes, resolutions
Trend analysis	MMM drift detection	Feature drift patterns, temporal trends
Risk management update	Threshold comparison	New risks identified, severity reassessment
**4. Corrective Actions**		
CAPAs initiated	ALARM/REJECT events	List of CAPAs, status, effectiveness
Model updates	CDM APPROVE decisions	Retraining events, performance changes
Process improvements	CAPA outcomes	SOP updates, threshold revisions
**5. Benefit-Risk Conclusion**		
Overall assessment	Aggregate CDM decisions	Decision distribution analysis
Benefit-risk ratio	Clinical metrics vs. incidents	Updated benefit-risk determination
Recommendations	Infrastructure analysis	Continue/modify/withdraw decision

S3.7.2 PSUR Executive Summary Template

Table S26: PSUR Executive Summary Template

PSUR — Executive Summary

**Device:**	________
**Reporting Period:**	________ to ________
**AEGIS Infrastructure Version:**	________________

Summary Statistics

Category	Count	Percentage
Total AEGIS iterations	________	—
Total samples processed	________	—
APPROVE decisions	________	________%
CONDITIONAL APPROVAL	________	________%
CLINICAL REVIEW	________	________%
REJECT decisions	________	________%
PMS ALARM co-issued	________	________% of iterations
of which: APPROVE + ALARM	________	
of which: REJECT + ALARM	________	

Performance Summary

Metric	Baseline	Current	Change
Sensitivity	________	________	________
Specificity	________	________	________
ROC-AUC	________	________	________
MLcps	________	________	________

Drift Analysis

Major drift events:	________
Minor drift events:	________
Features with significant shift:	________

Benefit-Risk Conclusion

☐ **Unchanged** — Benefit-risk remains favorable
☐ **Improved** — Enhanced performance or reduced risk
☐ **Requires attention** — Monitoring indicates potential concerns
☐ **Action required** — Benefit-risk reassessment needed

Recommendations: ________

S4 Robustness Analyses

S4.1 Sepsis Prediction Instantiation

All values below were generated on the real PhysioNet Set A/Set B features at the manuscript configuration (single Random Forest, 300 trees, seed 42; bootstrap $B = 1000$); the deployment-decision sequence reproduces main-text Table 8 exactly. The machine-readable outputs are in

the code repository (`section8_*.csv`, `section9_*.csv`).

Table S27: Per-iteration robustness summary (sepsis). Conf. = bootstrap decision confidence (B=1000); P(ALM) = bootstrap probability of an ALARM; SHAP conc. = top-5 importance concentration; Rank corr. = Spearman correlation of feature ranks vs. iteration 0; Bias = |Δsensitivity| on the Age median split.

Iter.	Decision	Conf.	P(ALM)	Brier	ECE	SHAP conc.	Rank corr.	Bias
0	APPR.	0.75	0.00	0.053	0.095	0.302	—	0.035
1	APPR.	0.94	0.00	0.053	0.096	0.300	0.953	0.027
2	APPR.	0.93	0.00	0.053	0.101	0.306	0.951	0.005
3	APPR.	0.93	0.00	0.054	0.104	0.301	0.925	0.022
4	APPR.	0.93	0.00	0.054	0.106	0.298	0.920	0.001
5	APPR.	0.97	0.00	0.054	0.108	0.303	0.905	0.010
6	COND. APPR.	1.00	0.00	0.060	0.134	0.292	0.908	0.001
7	CLIN. REV.	0.59	0.00	0.060	0.133	0.289	0.917	0.021
8	APPR. +ALARM	0.93	1.00	0.060	0.132	0.300	0.910	0.043
9	APPR.	1.00	0.00	0.071	0.171	0.285	0.898	0.019
10	REJ. +ALARM	1.00	1.00	0.066	0.148	0.336	0.587	0.040

Table S28: Component ablation (sepsis): deployment decision under progressively richer logic. A0 = safety floor only; A1 = +fixed-reference/buffer; A1b = +minor-drift; A2 = full system + PMS ALARM. Iterations 0–5 and 9 are APPROVE under all configurations and are omitted.

Iter.	A0 (floor)	A1 (+fixed ref)	A1b (+minor)	A2 (full)	Added by
6	APPR.	APPR.	COND. APPR.	COND. APPR.	minor drift
7	APPR.	CLIN. REV.	CLIN. REV.	CLIN. REV.	fixed reference
8	APPR.	CLIN. REV.	CLIN. REV.	APPR. +ALARM	PMS ALARM/ defer
10	REJ.	REJ.	REJ.	REJ. +ALARM	PMS ALARM

Table S29: Fairness subgroup gaps (sepsis): max−min sensitivity/specificity gap across Age tertiles and between sexes, per iteration. Minimum subgroup size 1354 (Age tertile), 1674 (sex). No fairness condition changed any deployment decision.

Iter.	Age sens. gap	Age spec. gap	Sex sens. gap	Sex spec. gap
0	0.105	0.025	0.077	0.006
1	0.091	0.015	0.024	0.004
2	0.114	0.020	0.057	0.006
3	0.083	0.019	0.053	0.009
4	0.114	0.018	0.119	0.006
5	0.099	0.017	0.060	0.010
6	0.085	0.012	0.043	0.010
7	0.104	0.013	0.074	0.005
8	0.097	0.009	0.098	0.004
9	0.063	0.018	0.026	0.016
10	0.117	0.002	0.001	0.002

The real cross-site drift-detector operating characteristic (Hospital A vs. Hospital B) is reported in the main text (cross-site operating-characteristic table): ROC-AUC rising from 0.82 (n=25) to 1.00 ($n \geq 100$), zero observed false alarms at the 0.30 threshold, and detection of cross-site shift increasing with batch size.

Table S30: Threshold-sensitivity (sepsis): each key threshold perturbed by ±0.05 (finer grid for the gold-standard tolerance). Only two iterations ever change, and only under large shifts; widening the minor-drift band changes no decision.

Threshold	Values tested	Decision change
critical sensitivity	0.60 / 0.65 / 0.70	it8 APPR. →REJ. (only at 0.70)
review sensitivity	0.61 / 0.66 / 0.71	it8 APPR. →CLIN. REV. (only at 0.71)
review specificity	0.55 / 0.60 / 0.65	none
minor-drift lower	0.25 / 0.30 / 0.35 / 0.40	none
minor-drift upper	0.65 / 0.70 / 0.75	none
major-drift threshold	0.85 / 0.90 / 0.95	none
fixed-ref tolerance	0.00–0.05	it7 CLIN. REV. →APPR. (at ≥0.03)

S4.2 Brain Tumor Segmentation Instantiation

The following tables present post-hoc robustness analyses for the glioma segmentation lifecycle described in Section 3.2 of the main manuscript. All analyses use the 12-iteration Brain Tumor Segmentation (BraTS)-derived sequence at the manuscript configuration (nnU-Net ResENC-M v2, $\tau^{\text{safe}} = 0.05$ safety margin, $\tau^{\text{reg}} = 0.015$ regression tolerance, $P^{\text{ref}} = 0.726$). Tables S31 and S32 characterize the sensitivity of the CDM to variations in the safety floor margin and regression tolerance respectively. Table S33 presents a framework compatibility simulation verifying that the full four-level taxonomy is reachable on volumetric imaging data. Tables S34 and S35 demonstrate MMM metric extensibility across alternative tumoral subregions and surface-distance criteria.

Table S31: **Sensitivity analysis of the Glioma performance floor across retraining iterations.** Deployment statuses are classified as APPROVE (APPR.) or REJECT (REJ.) based on the safety floor ($P^{\text{ref}} - \tau^{\text{safe}}$) derived from the iteration-0 baseline of $P^{\text{ref}} = 0.726$. Bold row ($\tau^{\text{safe}} = 0.05$, $P^{\text{fail}} = 0.676$) is the primary manuscript configuration.

		Iterations											
τ^{safe}	P^{fail}	**1**	**2**	**3**	**4**	**5**	**6**	**7**	**8**	**9**	**10**	**11**	**12**
0.01	0.716	REJ.	REJ.	REJ.	REJ.	APPR.	APPR.	APPR.	REJ.	REJ.	REJ.	REJ.	REJ.
0.02	0.706	REJ.	REJ.	REJ.	REJ.	APPR.	APPR.	APPR.	REJ.	REJ.	REJ.	REJ.	REJ.
0.03	0.696	REJ.	REJ.	REJ.	APPR.	APPR.	APPR.	APPR.	REJ.	REJ.	REJ.	REJ.	REJ.
0.04	0.686	REJ.	REJ.	APPR.	APPR.	APPR.	APPR.	APPR.	REJ.	REJ.	REJ.	REJ.	REJ.
**0.05**	**0.676**	**REJ.**	**REJ.**	**APPR.**	**APPR.**	**APPR.**	**APPR.**	**APPR.**	**REJ.**	**REJ.**	**REJ.**	**REJ.**	**REJ.**
0.06	0.666	REJ.	APPR.	APPR.	APPR.	APPR.	APPR.	APPR.	REJ.	REJ.	REJ.	REJ.	REJ.
0.07	0.656	REJ.	APPR.	APPR.	APPR.	APPR.	APPR.	APPR.	REJ.	REJ.	REJ.	REJ.	REJ.
0.08	0.646	REJ.	APPR.	APPR.	APPR.	APPR.	APPR.	APPR.	REJ.	REJ.	REJ.	REJ.	REJ.
0.09	0.636	APPR.	APPR.	APPR.	APPR.	APPR.	APPR.	APPR.	REJ.	REJ.	REJ.	REJ.	REJ.
0.10	0.626	APPR.	APPR.	APPR.	APPR.	APPR.	APPR.	APPR.	REJ.	REJ.	REJ.	REJ.	REJ.

Table S32: **Sensitivity analysis of the glioma regression tolerance (τ^{reg}) boundary across retraining iterations.** Deployment statuses are classified as APPROVE (APPR.) or REJECT (REJ.) based on the change-control logic against the rolling released reference P_G^{rel}. Bold row ($\tau^{\text{reg}} = 0.000$) is the primary manuscript configuration.

	Iterations											
τ^{reg}	**1**	**2**	**3**	**4**	**5**	**6**	**7**	**8**	**9**	**10**	**11**	**12**
**0.000**	**REJ.**	**REJ.**	**APPR.**	**APPR.**	**APPR.**	**APPR.**	**APPR.**	**REJ.**	**REJ.**	**REJ.**	**REJ.**	**REJ.**
0.010	REJ.	REJ.	APPR.	APPR.	APPR.	APPR.	APPR.	REJ.	REJ.	REJ.	REJ.	REJ.
0.011	REJ.	REJ.	APPR.	APPR.	APPR.	APPR.	APPR.	REJ.	REJ.	REJ.	REJ.	REJ.
0.012	REJ.	REJ.	APPR.	APPR.	APPR.	APPR.	APPR.	REJ.	REJ.	REJ.	REJ.	REJ.
0.013	REJ.	REJ.	APPR.	APPR.	APPR.	APPR.	APPR.	REJ.	REJ.	REJ.	REJ.	REJ.
0.014	REJ.	REJ.	APPR.	APPR.	APPR.	APPR.	APPR.	REJ.	REJ.	REJ.	REJ.	REJ.
0.015	REJ.	REJ.	APPR.	APPR.	APPR.	APPR.	APPR.	REJ.	REJ.	REJ.	REJ.	REJ.
0.016	REJ.	REJ.	APPR.	APPR.	APPR.	APPR.	APPR.	REJ.	REJ.	REJ.	REJ.	REJ.
0.017	REJ.	REJ.	APPR.	APPR.	APPR.	APPR.	APPR.	REJ.	REJ.	REJ.	REJ.	REJ.
0.018	REJ.	REJ.	APPR.	APPR.	APPR.	APPR.	APPR.	REJ.	REJ.	REJ.	REJ.	REJ.
0.019	REJ.	REJ.	APPR.	APPR.	APPR.	APPR.	APPR.	APPR.	APPR.	APPR.	APPR.	APPR.
0.020	REJ.	REJ.	APPR.	APPR.	APPR.	APPR.	APPR.	APPR.	APPR.	APPR.	APPR.	APPR.

Table S33: **Framework compatibility simulation mapping the full four-level decision taxonomy onto the glioma dataset.** This simulation confirms that the AEGIS modular engine natively supports multi-tiered classification logic on volumetric imaging data ($P^{\text{fail}} = 0.676$; $\mathcal{S}_D$= Drift score).

**Iter.** k	$P_{G,k}$	$\mathcal{S}_D$	**Trigger**	**Deploy. Decision**	**PMS Signal**
1	0.646	0.000	$P_{G,1} < P^{\text{fail}}$ (Safety Violation)	REJ.	—
2	0.673	0.000	$P_{G,2} < P^{\text{fail}}$ (Safety Violation)	REJ.	—
3	0.690	0.000	$P_{G,3}$ < review threshold 0.880; ALARM: $P_{D,3} < P_{D,2}$ (Drifting drop)	CLIN. REV.	ALARM
4	0.700	0.000	$P_{G,4}$ < review threshold 0.880	CLIN. REV.	—
5	0.725	0.000	$P_{G,5}$ < review threshold 0.880; ALARM: $P_{D,5} < P_{D,4}$ (Drifting drop)	CLIN. REV.	ALARM
6	0.726	0.450	$P_{G,6}$ < review threshold 0.880	CLIN. REV.	—
7	0.742	0.000	$P_{G,7}$ < review threshold 0.880	CLIN. REV.	—
8	0.723	0.950	$P_{G,8}$ < review threshold 0.880	CLIN. REV.	—
9	0.714	0.000	$P_{G,9}$ < review threshold 0.880; ALARM: $P_{D,9} < P_{D,8}$ (Drifting drop)	CLIN. REV.	ALARM
10	0.706	0.000	$P_{G,10}$ < review threshold 0.880	CLIN. REV.	—
11	0.707	0.000	$P_{G,11}$ < review threshold 0.880	CLIN. REV.	—
12	0.698	0.000	$P_{G,12}$ < review threshold 0.880	CLIN. REV.	—

Table S34: **Post-market change-control performance mapping across the Whole Tumor (WT) radiological region using volumetric Dice Similarity Coefficient (DSC).** The deployment safety floor is $P^{\text{fail}} = 0.844$. All 12 iterations satisfy the safety floor; PMS ALARM signals are co-issued at iterations 3, 4, and 9 due to Drifting set performance drops, without interrupting deployment.

**Iter.** k	$P_{G,k}$	$P_{D,k}$	**Trigger**	**Routing Trace**	**Deploy. Decision**	**PMS Signal**
1	0.867	0.908	All satisfied	P1✓ →P4	APPR.	–
2	0.880	0.981	All satisfied	P1✓ →P4	APPR.	–
3	0.891	0.922	$P_{D,3} < P_{D,2}$ (Drifting drop)	P1✓ →P4	APPR.	ALARM
4	0.892	0.880	$P_{D,4} < P_{D,3}$ (Drifting drop)	P1✓ →P4	APPR.	ALARM
5	0.891	0.882	All satisfied	P1✓ →P4	APPR.	–
6	0.895	0.902	All satisfied	P1✓ →P4	APPR.	–
7	0.896	0.913	All satisfied	P1✓ →P4	APPR.	–
8	0.897	0.936	All satisfied	P1✓ →P4	APPR.	–
9	0.896	0.905	$P_{D,9} < P_{D,8}$ (Drifting drop)	P1✓ →P4	APPR.	ALARM
10	0.896	0.915	All satisfied	P1✓ →P4	APPR.	–
11	0.901	0.977	All satisfied	P1✓ →P4	APPR.	–
12	0.898	N/A	All satisfied	P1✓ →P4	APPR.	–

Table S35: **Post-market change-control performance mapping across the Non-Enhancing Tumor Core (NETC) subregion using Normalized Surface Distance (NSD).** The deployment safety floor is $P^{\text{fail}} = 0.614$. Iterations 1–3 are REJECT (REJ.) due to safety floor violations. Iteration 10 is REJECT due to a non-regression gate trip: the $P_{G,k} = 0.640$ falls more than $\tau^{\text{reg}} = 0.015$ below the released performance reference $P_G^{\text{rel}} = 0.657$ (i.e., $0.657 - 0.015 = 0.642$). PMS ALARM signals are co-issued at iterations 6, 8, and 11.

**Iter.** k	$P_{G,k}$	$P_{D,k}$	**Trigger**	**Routing Trace**	**Deploy. Decision**	**PMS Signal**
1	0.589	0.483	$P_{G,1} < P^{\text{fail}}$ (Safety Violation)	→P1	REJ.	–
2	0.603	0.527	$P_{G,2} < P^{\text{fail}}$ (Safety Violation)	→P1	REJ.	–
3	0.613	0.527	$P_{G,3} < P^{\text{fail}}$ (Safety Violation)	→P1	REJ.	–
4	0.621	0.552	All satisfied	P1✓ →P4	APPR.	–
5	0.649	0.581	All satisfied	P1✓ →P4	APPR.	–
6	0.663	0.556	$P_{D,6} < P_{D,5}$ (Drifting drop)	P1✓ →P4	APPR.	ALARM
7	0.658	0.606	All satisfied	P1✓ →P4	APPR.	–
8	0.655	0.602	$P_{D,8} < P_{D,7}$ (Drifting drop)	P1✓ →P4	APPR.	ALARM
9	0.657	0.625	All satisfied	P1✓ →P4	APPR.	–
10	0.640	0.652	$P_{G,10} < P_G^{rel} - \tau = 0.642$ (Non-regression trip)	→P1	REJ.	–
11	0.656	0.590	$P_{D,11} < P_{D,10}$ (Drifting drop)	P1✓ →P4	APPR.	ALARM
12	0.649	N/A	All satisfied	P1✓ →P4	APPR.	–

A Example Configuration — Sepsis Prediction

This appendix provides a fully worked, end-to-end configuration for the sepsis prediction instantiation described in Section 3.1 of the main manuscript, demonstrating how the abstract infrastructure parameters defined in Sections S2.1–S2.8 are concretized for a specific clinical application. The completed configuration template (Table S36) covers all aspects of the instantiation—device identification, data modality and model architecture, primary metrics and MLcps weights, drift detection procedure, and the complete threshold configuration for all four decision categories and three ALARM conditions—together with the gold standard baseline metrics established from the model trained on the full PhysioNet Set A cohort. Organizations implementing for sepsis prediction, or for any tabular Electronic Health Record (EHR)-based prediction task with similar characteristics, should use this appendix as a concrete reference to validate that their own completed template is structurally complete and clinically coherent.

Table S36: Completed Configuration Template for the Sepsis Prediction Instantiation

Device Identification

Device/System Name	AEGIS-Sepsis: Sepsis Early Warning System
Version	1.0
Intended Use	Early prediction of sepsis onset in hospitalized patients
Target Population	Adult patients (≥18 years) in acute care settings
Data Source	PhysioNet/CinC Challenge 2019

Instantiation Parameters

Data Modality	Tabular EHR time-series (202 features)
Model Architecture	Single Random Forest (RF) (300 trees, depth 12, min split 10, min leaf 5, balanced weighting, Out-Of-Bag (OOB) enabled)
Primary Metrics	Sensitivity, Specificity, ROC-AUC, MLcps
Drift Detection	Bonferroni-corrected KS test on 34 key features ($\alpha' = 0.05/34$)

MLcps Weights (Raw — package normalizes internally)

Metric	Weight (Raw)	Rationale
Sensitivity	1.5	Highest priority: missed sepsis cases are life-threatening
ROC-AUC	1.3	Overall discriminative ability
Balanced Accuracy	1.1	Account for class imbalance
Specificity	1.0	Minimize false alarms (alert fatigue)

Threshold Configuration (Two-Tier Structure, per Table VI)

Critical Safety Thresholds (REJECT triggers):

Metric	Threshold	Hazard Ref	Decision
Sensitivity	<0.65	H-001	REJECT

Performance Thresholds (CLINICAL REVIEW triggers):

Metric	Threshold	Hazard Ref	Decision
Sensitivity	<0.66	H-001	CLINICAL REVIEW
Specificity	<0.60	H-002	CLINICAL REVIEW
Gold std.	>0.015 decline	H-001	CLINICAL REVIEW

PMS ALARM Thresholds (Track 2 — parallel, released model):

Trigger	Threshold	Ref	Signal
A1: P^{PMS}	Sens. on $\mathcal{D}_{D,k} < 0.65$	—	PMS ALARM
A2: τ^{PMS}	Regression > 0.020 sens. pts	—	PMS ALARM
A3: Major drift	Drift score > 0.90	—	PMS ALARM

Minor Drift / TAI Thresholds (CONDITIONAL APPROVAL— P3):

Metric	Threshold	Ref	Decision
Drift Score	0.30–0.70	—	CONDITIONAL APPROVAL *
TAI Scores	<0.55	—	CONDITIONAL APPROVAL

*Minor drift range widened from [0.35, 0.70] to [0.30, 0.70] based on threshold calibration analysis of stationary baseline variability (iterations 0–5).

Gold Standard Baseline (from Iteration 0)

Metric	Baseline	Margin (τ^{buf})	Lower Bound
Sensitivity	0.723	0.015	0.708
Specificity	0.933	0.01	0.923
ROC-AUC	0.922	0.01	0.912
MLcps	0.721	0.02	0.701

B Example Configuration — Brain Tumor Segmentation

This appendix provides a fully worked configuration for the brain tumor segmentation instantiation described in Section 3.2 of the main manuscript, illustrating how the infrastructure can be re-configured for a volumetric imaging task with a substantially different clinical risk profile and a reduced set of active CDM decision categories. Notably, this instantiation activates only the APPROVE, APPROVE + ALARM, and REJECT decision categories, deliberately omitting CONDITIONAL APPROVAL and CLINICAL REVIEW: for radiotherapy and surgical planning applications, where a model either meets the non-inferiority threshold relative to the released reference (P_G^{rel}) or it does not, intermediate deployment states carry limited clinical utility, and the strict binary structure better reflects the treatment-planning context.

Table S37: Completed Configuration Template for the Brain Tumor Segmentation Instantiation

Device Identification

Device/System Name	AEGIS-BraTS: Brain Tumor Segmentation Governance
Version	1.0
Intended Use	Automated segmentation of brain tumors (Tumor Core) in multi-parametric MRI (mpMRI) for radiotherapy and surgical planning
Target Population	Adult patients with glioma (pre-operative and post-treatment)
Data Source	BraTS 2023/2024 Challenge (GLI cohorts)

Instantiation Parameters

Data Modality	3D multi-parametric MRI (T1n, T1c, T2w, T2f)
Model Architecture	nnU-Net ResENC-M (v2), 1,500 epochs, default configuration
Primary Metric	DSC on Tumor Core (TC = ET + NETC)
MLcps Weighting	DSC: 1 (single metric)
Drift Detection	Performance comparison (Golden vs. Drifting DSC)

Threshold Configuration

Metric	Threshold	Derivation	Decision
P^{ref}	DSC: 0.726	Full-data model on the Golden set	Reference baseline
τ^{safe}	0.05	Clinically acceptable margin	Defines P^{fail}
P^{fail}	DSC: 0.676	$P^{\text{ref}} - \tau^{\text{safe}}$	REJECT (below 0.676)

Decision Logic (Reduced-Category Configuration)

REJECT	$P_{G,k} < 0.676$ (safety floor) OR $P_{G,k} < P_G^{\text{rel}}$ (non-regression)
APPROVE	$P_{G,k} > P_G^{\text{rel}}$ AND $P_{D,k} > P_{D,k-1}$
APPROVE + ALARM	$P_{G,k} > P_G^{\text{rel}}$ (primary: APPROVE) AND $P_{D,k} < P_{D,k-1}$ (PMS ALARM co-issued: drifting set declined)

Note on active category configuration: CONDITIONAL APPROVAL and CLINICAL REVIEW were intentionally not activated for this instantiation. Brain tumor segmentation for treatment planning is a high-consequence application where intermediate deployment states carry limited clinical utility — a model either meets the non-inferiority threshold or it does not. This is a deliberate configuration choice, not a limitation. The ALARM signal retains its role as a parallel PMS signal and fires when the drifting set performance declines (trigger C^{R}). Production implementations should document the active category set and its clinical justification in the PCCP.

Gold Standard Baseline (from Iteration 0)

Metric	Baseline	Margin (τ^{safe})	Lower Bound
DSC (TC)	0.726	0.05	0.676

C Decision Category Quick Reference

This appendix provides a concise reference table for the five possible output states—APPROVE, CONDITIONAL APPROVAL, CLINICAL REVIEW, REJECT, and PMS ALARM —intended for use by clinical, regulatory, and engineering staff who need rapid access to the operational meaning, FDA regulatory status, and EU regulatory status of each CDM output without consulting the full main manuscript. The table is organized to make the distinction between the four deployment decision categories (which evaluate the new candidate model) and the independent PMS ALARM signal (which evaluates the currently released model) immediately apparent; the decision hierarchy summary below the table reinforces the priority ordering that governs how conflicts among conditions are resolved.

Table S38: Decision Category Quick Reference

Decision	Definition	FDA Status	EU Status
**APPROVE**	All metrics satisfied; deploy immediately	Within PCCP scope; no new 510(k)	Art. 43(4) predetermined change exemption
**CONDITIONAL APPROVAL**	Minor concerns; deploy with enhanced monitoring	Within PCCP scope; enhanced monitoring	Art. 83(2) enhanced PMS
**CLINICAL REVIEW**	Near threshold; human validation required	Edge of PCCP scope; human oversight first	Art. 52 NB consultation possible
**ALARM**	PMS signal: released model at risk in field; co-issued with primary decision independently	Activates PMS reporting; may require vigilance report	MDR Art. 83/87 PMS obligations
**REJECT**	Safety threshold failed; cannot deploy	Outside PCCP scope; new 510(k) likely	Art. 5(5); new Conformity Assessment (CA) required

Decision Hierarchy (Evaluation Order)

Track 1 (new model): SAFETY (P1) → PERFORMANCE/GOLD STD (P2) → MINOR DRIFT/TAI (P3) → APPROVE (P4)

Track 2 (released model, parallel): PMS ALARM fires if A1 (safety floor) OR A2 (regression) OR A3 (major drift)

Composite output: (P1–P4 decision) or (P1–P4 decision + **ALARM**)

Key Principle: The hierarchy ensures safety-critical checks are always evaluated first for the new candidate model. A model that fails safety boundaries will be REJECTED regardless of excellent performance in other dimensions.
ALARM is a parallel PMS signal, not a step in the deployment hierarchy. It fires when the *currently released model* is assessed as at risk on the new data batch — independently of whether the new model is approved or rejected. This produces composite outputs: APPROVE + ALARM (new model ready, released model at risk — accelerate deployment) or REJECT + ALARM (no deployable model and released model failing — activate contingency protocols immediately).
Configuration Note: The CONDITIONAL APPROVAL and CLINICAL REVIEW categories can be selectively enabled or disabled based on the clinical risk profile. Document the active category set and its justification in the PCCP submission.

D AEGIS CAPA Form Template

This appendix provides a standardized CAPA form designed specifically for events triggered by ALARM or REJECT decisions, adapted from OpenRegulatory ISO 13485 templates with -specific fields for the CDM trigger reason, affected metrics, and threshold violations. The form structure follows the lifecycle of a CAPA, providing a single document that serves simultaneously as the QMS record required by ISO 13485 and as the narrative evidence supporting the Corrective Actions and Safety Analysis sections of the PSUR. Organizations should configure their CAPA management system to generate this form automatically upon any ALARM or REJECT event, pre-populated with the relevant fields from the audit trail.

Table S39: AEGIS CAPA Form Template

CAPA Form — AEGIS-Triggered Event

**1. CAPA Identification**	CAPA ID:	________________
	Date Opened:	________________
	Opened By:	________________
	AEGIS Iteration:	________
	AEGIS Decision:	☐ ALARM ☐ REJECT
	Related AEGIS Trigger:	________________________________

2. Event Description

Describe the event that triggered the CAPA, including AEGIS decision details:

Decision Timestamp:	________________
Trigger Reason (from CDM):	________________________________
Affected Metric(s):	________________________________
Metric Value(s):	________________________________
Threshold(s) Violated:	________________________________

Narrative description:

____

____

____

3. Immediate Actions Taken

☐ Model deployment blocked (REJECT)
☐ Previous model version maintained
☐ Enhanced monitoring activated
☐ Stakeholders notified (list): ________________
☐ Vigilance process initiated (if applicable)
☐ Other: ________________________________

4. Root Cause Analysis (Five Whys)

Why 1: ________________________________

Why 2: ________________________________

Why 3: ________________________________

Why 4: ________________________________

Why 5: ________________________________

Root Cause Summary: ________________________________

5. Impact Assessment

Patient safety impact: ☐ None ☐ Potential ☐ Actual
Patients potentially affected: ________
Regulatory notification required: ☐ Yes ☐ No ☐ Under assessment
Risk management file update needed: ☐ Yes ☐ No

6. Corrective Actions

#	Action	Responsible	Due Date	Status
1	________	________	________	☐
2	________	________	________	☐
3	________	________	________	☐
4	________	________	________	☐

7. Preventive Actions

#	Action	Responsible	Due Date	Status
1	________	________	________	☐
2	________	________	________	☐
3	________	________	________	☐

8. AEGIS Infrastructure Updates (if applicable)

Threshold adjustment required: ☐ Yes ☐ No
If yes, specify: ________________________________
MLcps weight adjustment: ☐ Yes ☐ No
Drift detection update: ☐ Yes ☐ No
Gold standard refresh: ☐ Yes ☐ No

9. Effectiveness Verification

Verification method: ________________________________
Verification date: ________________
Verified by: ________________
Verification result: ☐ Effective ☐ Not effective ☐ Partial
Evidence of effectiveness: ________________________________

10. CAPA Closure

Closure date: ________________
Closed by: ________________
PRRC review: ☐ Approved ☐ Requires further action

Closure notes: ________________________________

List of Figures

S1 Decision Priority Hierarchy . . . 39
S2 AEGIS Infrastructure Adoption Workflow . . . 40
S3 **Two-track architecture.** The left flowchart shows the new model deployment decision (P1–P4). In parallel, the PMS ALARM signal evaluates the *currently released model* on each new data batch: A1 (safety floor breach), A2 (regression from release baseline), A3 (major distributional shift). The composite output is (primary decision) or (primary decision + ALARM). Both tracks run at every iteration regardless of each other's outcome. . . . 41
S4 PCCP Submission Structure . . . 42
S5 Escalation Protocol Matrix . . . 43

```
== TRACK 1: NEW MODEL DEPLOYMENT DECISION (priority-ordered) ==

PRIORITY 1: SAFETY CHECK (new candidate model on D_G)
    IF sensitivity < 0.65 THEN --> REJECT [P1]

PRIORITY 2: PERFORMANCE BUFFER / GOLD STANDARD CHECK
    IF sensitivity < 0.66 THEN --> CLINICAL_REVIEW [P2]
    IF specificity < 0.60 THEN --> CLINICAL_REVIEW [P2]
    IF gold_standard_regression > 0.015 THEN --> CLINICAL_REVIEW [P2]

PRIORITY 3: MINOR DRIFT / TRUSTWORTHY AI CHECK
    IF drift_score >= 0.30 AND
       drift_score <  0.70 THEN --> CONDITIONAL_APPROVAL [P3]
    IF any_TAI_score < 0.55 THEN --> CONDITIONAL_APPROVAL [P3] (illustrative; not triggered)

PRIORITY 4: ALL PASS -> APPROVE
    IF all_checks_pass THEN --> APPROVE [P4]

== TRACK 2: PMS ALARM SIGNAL (released model on D_D; fires if ANY true) ==

    A1: IF released_model_sens_on_DD < 0.65 THEN --> ALARM
    A2: IF released_model_perf_on_DD <= P_rel_baseline -  0.02 THEN --> ALARM
    A3: IF drift_score > 0.90 THEN --> ALARM

Composite output = (Track 1 decision)
                OR (Track 1 decision + ALARM) when any of A1-A3 fires.
Absence of ALARM requires no label.
```

Figure S1: Decision Priority Hierarchy

```
+--------------------------------------------------------------------------+
|                     INFRASTRUCTURE ADOPTION WORKFLOW                      |
+--------------------------------------------------------------------------+

PHASE 1: RISK ASSESSMENT
+----------------------------------------+
| * Conduct ISO 14971:2019 hazard        |
|   analysis                             |
| * Define severity classifications      |
| * Document risk-benefit analysis       |
| * Identify safety-critical metrics     |
+-----------------+----------------------+
                  |
                  v
PHASE 2: THRESHOLD DERIVATION
+----------------------------------------+
| * Map hazards to metric thresholds     |
| * Define acceptance margins (tau)      |
| * Establish decision boundaries        |
| * Document clinical rationale          |
+-----------------+----------------------+
                  |
                  v
PHASE 3: MODULE CONFIGURATION
+----------------------------------------+
| * Configure DARM (data governance)     |
| * Configure MMM (monitoring metrics)   |
| * Configure CDM (decision logic)       |
| * Implement drift detection            |
+-----------------+----------------------+
                  |
                  v
PHASE 4: VALIDATION
+----------------------------------------+
| * Test with historical data            |
| * Verify decision logic correctness    |
| * Validate audit trail completeness    |
| * Conduct sensitivity analysis         |
+-----------------+----------------------+
                  |
                  v
PHASE 5: REGULATORY DOCUMENTATION
+----------------------------------------+
| * Complete PCCP documentation          |
| * Prepare EU MDR technical file        |
| * Document AI Act compliance           |
| * Prepare submission package           |
+-----------------+----------------------+
                  |
                  v
PHASE 6: DEPLOYMENT
+----------------------------------------+
| * Establish gold standard baseline     |
| * Activate monitoring                  |
| * Begin operational governance         |
| * Initiate post-market surveillance    |
+----------------------------------------+
```

Figure S2: AEGIS Infrastructure Adoption Workflow

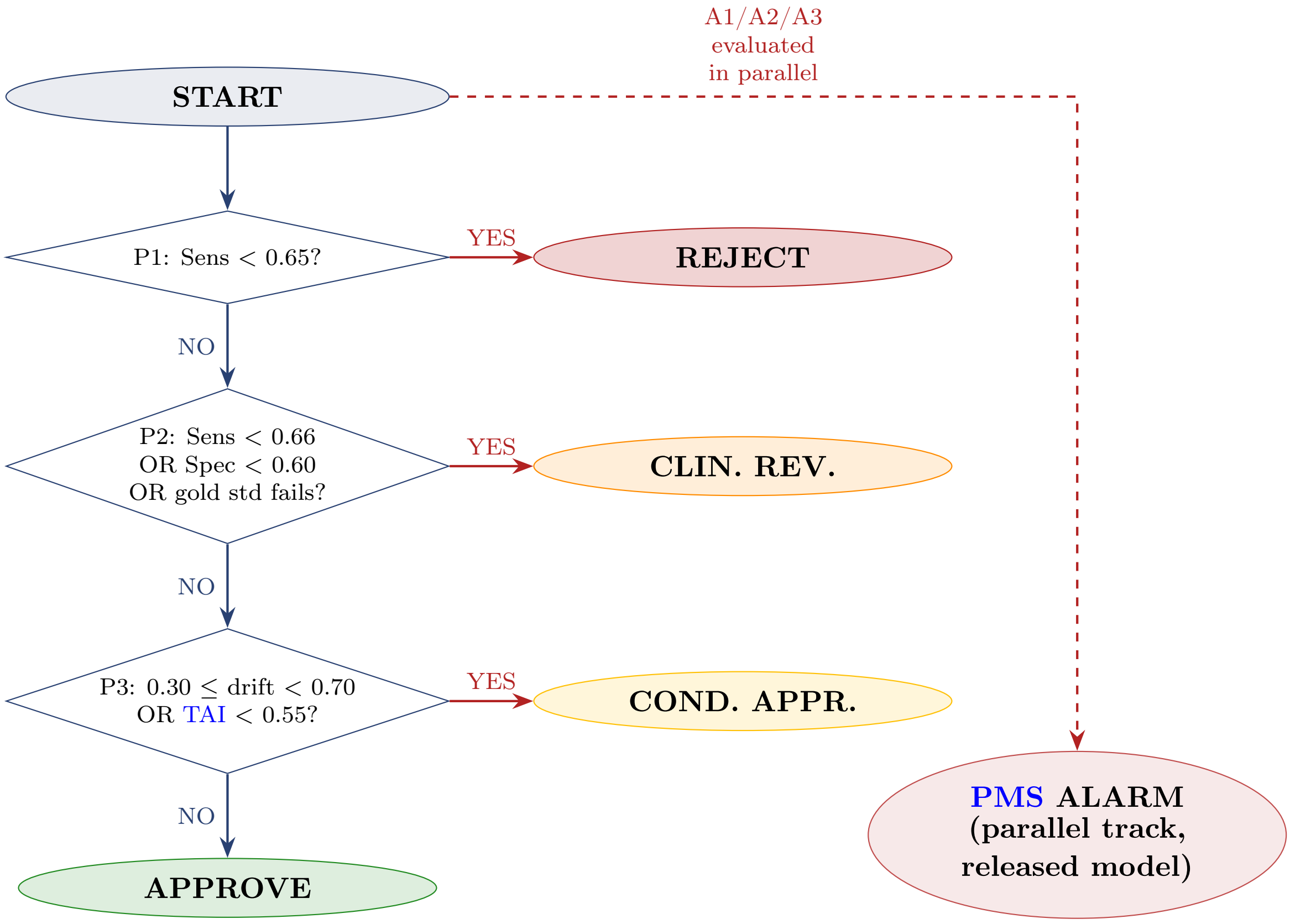

Figure S3: **Two-track architecture.** The left flowchart shows the new model deployment decision (P1–P4). In parallel, the PMS ALARM signal evaluates the *currently released model* on each new data batch: A1 (safety floor breach), A2 (regression from release baseline), A3 (major distributional shift). The composite output is (primary decision) or (primary decision + ALARM). Both tracks run at every iteration regardless of each other's outcome.

```
+--------------------------------------------------------------------+
|                      PCCP SUBMISSION STRUCTURE                     |
+--------------------------------------------------------------------+
|                                                                    |
| SECTION 1: DESCRIPTION OF MODIFICATIONS                            |
| +-- 1.1 Modification types covered by PCCP                        |
| |   +-- Reference: DARM data assimilation protocols               |
| +-- 1.2 Modification boundaries (what changes are permitted)      |
| |   +-- Reference: CDM four-category deployment taxonomy +        |
| |       parallel PMS ALARM signal                                    |
| +-- 1.3 Modifications NOT covered (require new 510(k))            |
|     +-- Reference: REJECT criteria and escalation pathways        |
|                                                                    |
| SECTION 2: MODIFICATION PROTOCOL                                   |
| +-- 2.1 Data collection and quality procedures                    |
| |   +-- Reference: DARM data quality gates                        |
| +-- 2.2 Retraining procedures                                     |
| |   +-- Reference: DARM patient-safe splitting                    |
| +-- 2.3 Performance evaluation methodology                        |
| |   +-- Reference: MMM metric calculation procedures              |
| +-- 2.4 Verification and validation procedures                    |
|     +-- Reference: Gold standard comparison mechanism             |
|                                                                    |
| SECTION 3: IMPACT ASSESSMENT                                       |
| +-- 3.1 Performance acceptance criteria                           |
| |   +-- Reference: Threshold table with ISO 14971 traceability    |
| +-- 3.2 Safety impact analysis                                    |
| |   +-- Reference: Risk management file, hazard analysis          |
| +-- 3.3 Effectiveness impact analysis                             |
|     +-- Reference: Gold standard baseline, acceptance margins     |
|                                                                    |
+--------------------------------------------------------------------+
```

Figure S4: PCCP Submission Structure

```
+------------------------------------------------------------------+
|                    ESCALATION PROTOCOL MATRIX                    |
+------------------------------------------------------------------+
|                                                                  |
| APPROVE                                                          |
| +-- Action: Deploy model update                                  |
| +-- Documentation: Audit log entry                               |
| +-- Notification: None required                                  |
| +-- Timeline: Immediate                                          |
|                                                                  |
| CONDITIONAL APPROVAL                                             |
| +-- Action: Deploy with enhanced monitoring                      |
| +-- Documentation: Monitoring plan update                        |
| +-- Notification: Quality team                                   |
| +-- Timeline: Immediate, review in 30 days                       |
|                                                                  |
| CLINICAL REVIEW                                          |
| +-- Action: Hold deployment pending review                       |
| +-- Documentation: Clinical review request                       |
| +-- Notification: Clinical lead, Risk manager                    |
| +-- Timeline: Review within 5 business days                      |
|                                                                  |
| PMS ALARM SIGNAL (co-issued with primary decision)               |
| +-- Action: Investigate released model safety in field           |
| |   If co-issued with APPROVE: accelerate deployment of new model |
| |   If co-issued with REJECT: activate contingency protocols     |
| +-- Documentation: PMS incident report, root cause analysis      |
| +-- Notification: All stakeholders; Regulatory if A1/A2 breach   |
| +-- Timeline: Initial assessment within 24 hours                 |
|                                                                  |
| REJECT                                                           |
| +-- Action: Block deployment, maintain previous version          |
| +-- Documentation: Rejection report, CAPA initiation             |
| +-- Notification: All stakeholders, Regulatory                   |
| +-- Timeline: Immediate block, RCA within 48 hours               |
|                                                                  |
+------------------------------------------------------------------+
```

Figure S5: Escalation Protocol Matrix
